# Rationale and design of the THIRST Alert feasibility study: a pragmatic, single-centre, parallel-group randomised controlled trial of an interruptive alert for oral fluid restriction in patients treated with intravenous furosemide

**DOI:** 10.1136/bmjopen-2023-080410

**Published:** 2024-01-12

**Authors:** Yang Chen, Anoop Shah, Yogini Jani, Daniel Higgins, Nausheen Saleem, Kris Chafer, Matthew Robert Sydes, Folkert W Asselbergs, R Thomas Lumbers

**Affiliations:** 1 Institute of Health Informatics, University College London, London, UK; 2 Clinical and Research Informatics Unit, NIHR UCLH Biomedical Research Centre, University College London Hospitals NHS Foundation Trust, London, UK; 3 Centre for Medicines Optimisation Research & Education - CMORE, University College London Hospitals NHS Foundation Trust, London, UK; 4 Institute of Clinical Trials and Methodology, Medical Research Council Clinical Trials Unit at University College London, London, UK; 5 Health Data Research UK, London, UK; 6 Department of Cardiology, Amsterdam Cardiovascular Sciences, Amsterdam University Medical Centre, University of Amsterdam, Amsterdam, Netherlands

**Keywords:** Heart failure, Randomized Controlled Trial, Feasibility Studies, Electronic Health Records

## Abstract

**Introduction:**

Acute heart failure (HF) is a major cause of unplanned hospitalisation characterised by excess body water. A restriction in oral fluid intake is commonly imposed on patients as an adjunct to pharmacological therapy with loop diuretics, but there is a lack of evidence from traditional randomised controlled trials (RCTs) to support the safety and effectiveness of this intervention in the acute setting.

This study aims to explore the feasibility of using computer alerts within the electronic health record (EHR) system to invite clinical care teams to enrol patients into a pragmatic RCT at the time of clinical decision-making. It will additionally assess the effectiveness of using an alert to help address the clinical research question of whether oral fluid restriction is a safe and effective adjunct to pharmacological therapy for patients admitted with fluid overload.

**Methods and analysis:**

THIRST (Randomised Controlled **T**rial within the electronic **H**ealth record of an **I**nterruptive alert displaying a fluid **R**estriction **S**uggestion in patients with the treatable **T**rait of congestion) Alert is a single-centre, parallel-group, open-label pragmatic RCT embedded in the EHR system that will be conducted as a feasibility study at an National Health Service (NHS) hospital in London. The clinical care team will be invited to enrol suitable patients in the study using a point-of-care alert with a target sample size of 50 patients. Enrolled patients will then be randomised to either restricted or unrestricted oral fluid intake. Two primary outcomes will be explored (1) the proportion of eligible patients enrolled in the study and (2) the mean difference in oral fluid intake between randomised groups. A series of secondary outcomes are specified to evaluate the effectiveness of the alert, adherence to the randomised treatment allocation and the quality of data generated from routine care, relevant to the outcomes of interest.

**Ethics and dissemination:**

This study was approved by Riverside Research Ethics Committee (Ref: 22/LO/0889) and will be published on completion.

**Trial registration number:**

NCT05869656.

**STRENGTHS AND LIMITATIONS OF THIS STUDY**

- THIRST Alert is a pragmatic randomised controlled trial where all elements of trial conduct are embedded within the routine care clinical pathway.
- The intervention and programme theory has been codesigned with a multidisciplinary team including physicians, nurses, clinical informatics officers and patient representatives.
- A proportionate consent model has been approved for use to help with the practicality of trial conduct.
- Uncertainty remains regarding data quality in relation to the ascertainment of study outcomes from routine hospital care records.

## Introduction

Acute heart failure (HF) is an important cause of unplanned hospitalisation and is characterised by excess body water, also known as congestion.^1 2^ Despite being a common clinical problem, there is a lack of high-quality evidence to guide drug treatment and non-pharmacological measures.^3^ Restriction of oral fluid intake is commonly used as an adjunct to pharmacological therapy but its effectiveness is uncertain and may exacerbate symptoms of thirst, as suggested by the limited randomised controlled trials (RCTs), which have been conducted to date.^4–6^ These previous trials achieved only small differences in oral fluid intake, which may reflect the Hawthorne effect whereby participation in a clinical trial influences fluid intake in both groups.^7^

Patients and clinicians identify the need for better treatment of fluid overload as a key priority, and the use of oral fluid restriction also highlighted a gap in evidence in clinical practice guidelines.^2^ ^8^ ^9^ To address this evidence gap and some of the challenges encountered in previous studies, we designed a pragmatic RCT embedded in the electronic health record (EHR) system to evaluate the safety and effectiveness of oral fluid restriction in acute HF.

### Patient population

Fluid overload may be caused by liver or kidney dysfunction,^10 11^ as well as by HF, with similar evidence gaps for congestion management in such circumstances.^12–14^ However, a reliance on evaluating interventions for the immediate management of fluid overload only where the underlying cause is established may impact the generalisability of any findings. Given that standardised clinical scores for congestion are not well validated or adopted in practice,^15 16^ we used the prescription of two consecutive doses of intravenous furosemide within the first 48 hours of admission as evidence of a physician-assessed ‘treatable trait’ of fluid congestion, to define the study population.^17^

At our proposed study site, more than one consecutive dose of intravenous furosemide was administered during the first 48 hours of admission in 1537 unplanned admissions between April 2019 and October 2022. In 56% of these admissions with available supporting data, oral fluid restriction was recommended (online supplemental appendix 1). The use of prescribing intention to identify the study population enabled digital enrolment into the study based on a discrete triggering event. To our knowledge, there has been no previous RCT conducted in all patients treated for congestion with intravenous furosemide in the acute care setting.

10.1136/bmjopen-2023-080410.supp1 Supplementary data

### Pragmatic research

There are many evidence gaps in routine clinical practice that have not been addressed by conventional RCTs, which may be prohibitively expensive. Pragmatic clinical trials (PCTs) offer a potential solution, particularly relevant to the evaluation of low-risk interventions where clinical equipoise may exist. By integrating a randomisation procedure into routine care pathways, automating case identification through the EHR, engaging the clinical care team for patient recruitment and using routinely collected data for trial outcomes, there is the potential to perform large-scale trials at low-cost and low burden to patients and their caregivers. Many questions remain, however, about how best to deliver PCTs embedded in EHR systems, including the design of physician-facing alerts and different models of consent. For our study, we employ an interruptive alert to provide the routine care team with an invitation to enrol eligible patients, and use a proportionate process for consent that can be delivered by the same team.

PCTs have the potential to enable evidence generation during routine care, helping to conduct more efficient and representative trials.^18^ Through examining the comparative effectiveness of existing interventions that have demonstrable variation in practice, many evidence gaps may be addressed. The use of oral fluid restriction in patients who are treated pharmacologically for fluid overload in the setting of acute unplanned care represents one such gap.

The THIRST (Randomised Controlled **T**rial within the electronic **H**ealth record of an **I**nterruptive alert displaying a fluid **R**estriction **S**uggestion in patients with the treatable **T**rait of congestion) Alert trial, therefore aims to examine (1) whether interruptive alerts are an effective means to engage the usual care team to enrol patients into a pragmatic RCT and (2) for enrolled patients, whether the randomised assignment to either oral fluid restriction to 1 L per day or no oral fluid restriction leads to a difference in documented oral fluid intake. The evidence generated will be used to inform the design of subsequent multicentre outcomes-driven PCTs embedded in EHR systems.

## Methods and analysis

### Study design

THIRST Alert is a single-centre, parallel-group, open-label pragmatic RCT embedded in the EHR system that will be conducted as a feasibility study.^19^ The study will be delivered entirely at University College Hospital, UCLH, a digitally mature National Health Service (NHS) hospital.^20^ Enrolment started in May 2023 and the estimated trial completion date is December 2023. Given the low-risk nature of the intervention, a verbal, opt-out consent model is used. The study protocol has been prepared with reference to the Standard Protocol Items: Recommendations for Interventional Trials (SPIRIT) statement.^21^ The trial is registered at ClinicalTrials.gov (NCT05869656).

### Study procedures

**Figure 1:**
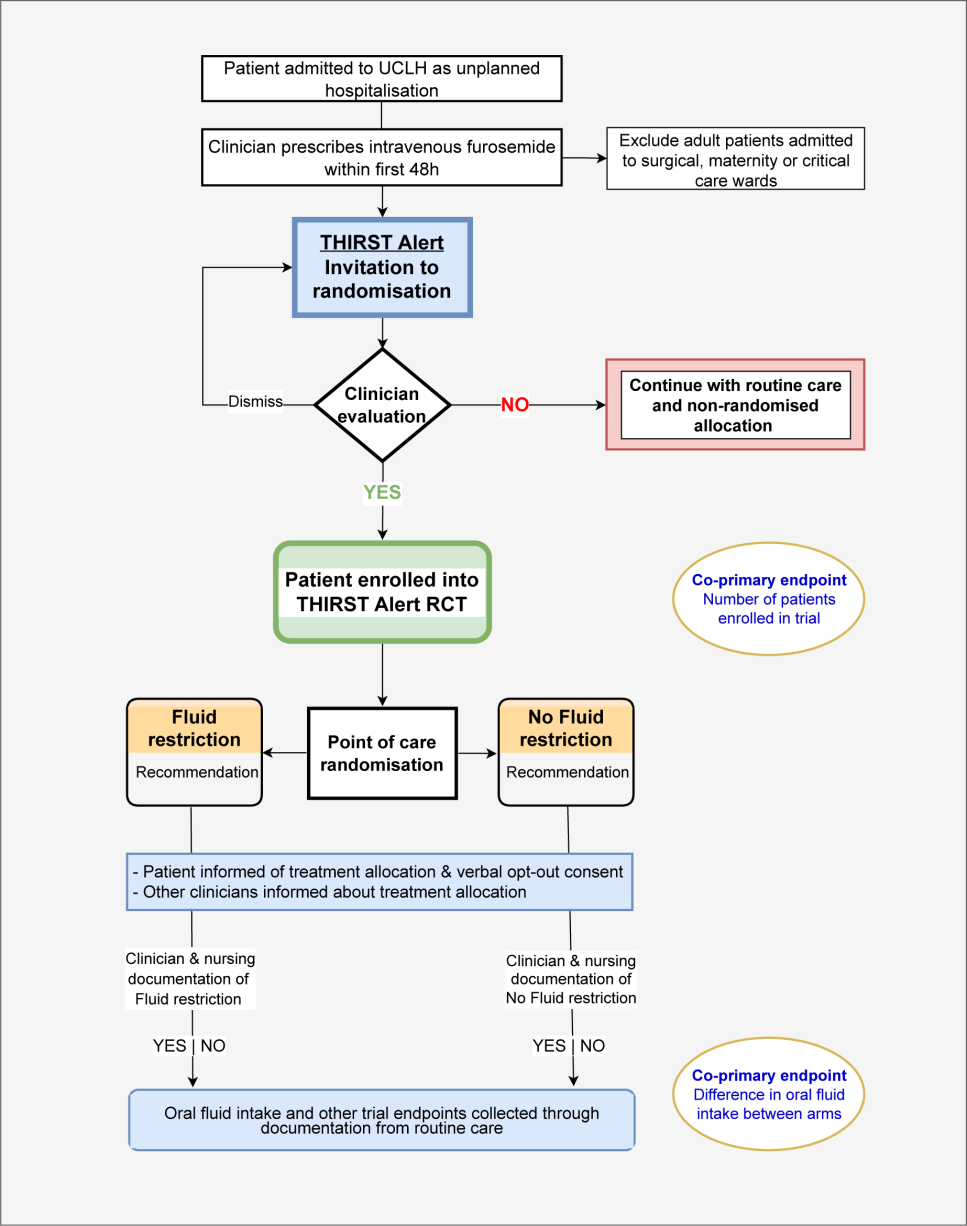
THIRST Alert trial patient flow. RCT, randomised controlled trial; THIRST, Randomised Controlled Trial within the electronic Health record of an Interruptive alert displaying a fluid Restriction Suggestion in patients with the treatable Trait of congestion; UCLH, University College London Hospital.

An overview of the study procedures is given in figure 1. The trial was designed to be integrated into the EHR system EPIC (Epic, Epic Systems, Verona, Wisconsin, USA). The screening and identification of eligible patients, display of interruptive alerts to physicians, randomisation and outcome ascertainment are all provisioned within EPIC.^22^ Education events, internal communication and email correspondence were delivered to members of the routine care team to raise awareness of the study but no formal cointerventions were undertaken. The conduct and analysis of the trial are overseen by a trial management group that conceived a programme theory for the alert with relevant multidisciplinary input (online supplemental appendix 2,3). The enrolment invitation and randomised treatment recommendations are classified as complex interventions according to the Medical Research Council framework.^23^

**Figure 2:**
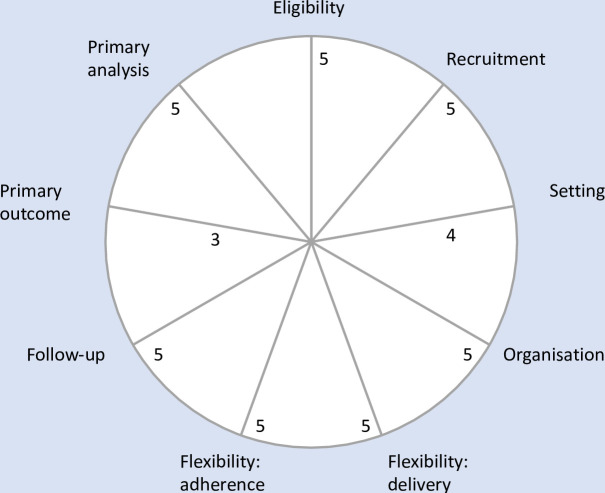
PRECIS-2 score for the THIRST Alert trial. Each of the nine domains are scored on a 5-point Likert continuum (from 1=very explanatory ‘ideal conditions’ to 5=very pragmatic ‘usual care conditions’). PRECIS-2 score, The PRagmatic Explanatory Continuum Indicator Summary-2 score; THIRST, Randomised Controlled Trial within the electronic Health record of an Interruptive alert displaying a fluid Restriction Suggestion in patients with the treatable Trait of congestion.

The trial is designed to maximise the generalisability and applicability of the evidence generated and is highly pragmatic according to the PRagmatic Explanatory Continuum Indicator Summary-2 (PRECIS-2) framework (figure 2).^24^

### Study participants

Adults of 18 years or older who were administered more than one dose of intravenous furosemide within the first 48 hours of an unplanned admission were eligible for the study. Patients admitted to a surgical, maternity or critical care ward were excluded (table 1).

**Table 1:** Eligibility criteria for thirst alert study participants

Study participants	Eligibility criteria
Patients	**Inclusion** Aged 18 years and over Prescribed intravenous furosemide during the first 48 hours of their admission as a regular prescription rather than a one-off dose. Assessed as being suitable for inclusion in the trial by the responsible clinical team, that is, fluid restriction deemed to be in equipoise. **Exclusion** Surgical or obstetric wards Critical care wards
Prescribing clinicians: physicians (consultants and junior doctors)	Any clinician with prescribing rights and who prescribes more than one dose of intravenous furosemide within 48 hours of admission, during the trial recruitment period.
Nursing staff	A separate order is triggered for enrolled patients and only presented to nursing staff who access their patient record to help facilitate fluid balance documentation.

### The interruptive alert inviting usual care team to enrol eligible patients

Any member of the clinical team may receive an interruptive alert (online supplemental appendix 4 figure 1A) during their interaction with EPIC for an eligible patient, informing them of the study and inviting them to enrol the patient. The clinician can click the appropriate button on the alert to enrol, decline to enrol or defer the decision regarding enrolment. The alert is non-coercive, and the decision about patient enrolment is at the discretion of the treating clinician. We anticipate that physicians will enrol patients in cases where they judge that there is clinical equipoise as to whether oral fluid restriction is likely to benefit the patient. The alert was designed and tested with EPIC application specialists in an iterative manner, integrating feedback from end-users on the wording of the design.

### The randomised treatment intervention

After enrolment of patients in the study, the routine care team receives a recommendation regarding oral fluid intake and assignment is determined by point of care randomisation. The randomised treatment allocation is only presented to the clinical team after they click ‘yes’ to the THIRST alert, rather than displayed immediately, to minimise the possibility of differential recruitment to the treatment arms of the trial. For patients allocated to the intervention group, the routine care team received a recommendation for a target oral fluid restriction of 1 L per day; for patients allocated to the control group, the care team received a recommendation for unrestricted oral fluid intake (online supplemental appendix 4 figure 1B,C). The oral fluid restriction target was chosen based on previous RCTs and acceptability in clinical practice.^4–6^ The randomised treatment recommendation for both groups additionally advises the clinician to: (1) complete an EPIC order for fluid balance monitoring; (2) document the treatment allocation in the clinical notes; (3) inform the patient and the nursing team and (4) provide the patient with a participant information sheet (PIS).

### Randomisation and blinding

Subjects will be randomised 1:1 using an internal random number rule implemented using a standard EPIC randomisation tool used in other PCTs.^25^ No block randomisation or additional covariate balancing will be undertaken. The study is open label with patients and their care teams being made aware of the treatment allocation.

### Study outcomes

#### Primary outcomes

1. Number of eligible patients randomised.
2. Difference in oral fluid intake between intervention and control arms.

#### Secondary outcomes

1. Adherence to randomised treatment recommendation.
2. Proportion of alerts resulting in clinical orders for nursing care.
3. Oral fluid intake.
4. Net fluid balance.
5. Weight change after randomisation.
6. Length of stay.
7. Frequency of blood test measurements of renal function.
8. Prescription of diuretic medications.
9. Daily change in creatinine.
10. Patient-reported outcome measures.

These outcomes were chosen based on clinical relevance and the ability to detect the possibility of attributable harm during an inpatient admission.

### Sample size and power calculation

No formal sample size calculation was performed given the primary outcomes pertain to the feasibility of using an EHR alert aimed at the routine care team to (1) enrol eligible patients into an RCT of oral fluid restriction and (2) cause and sustain the allocated clinical treatment effect, as measured by documented oral fluid intake. This is in keeping with the role of feasibility studies used by other groups.^26^ Our target sample size was 50 patients, based on the predicted number of eligible patients at the study site during the recruitment period.

### Data collection, curation and storage

Baseline characteristics and study outcomes will be extracted from the routine care record. Information on the frequency of the interruptive alert and staff responses to alerts will be recorded in EPIC. We will also extract primary and secondary International Classification of Diseases (ICD)-10 diagnosis codes assigned to the clinical episode by the clinical coding team. Study data will be transferred to a secure research environment within UCLH NHS Foundation Trust as pseudoanonymised electronic case report forms (eCRFs). No data will leave the UCLH NHS system. Patients who opt out or withdraw from the trial will have their decision documented on the eCRF and their data will not be included in the main analysis.

### Statistical analysis plan

Baseline characteristics of study participants will be summarised using percentages or means and SD as appropriate and compared using t tests for continuous variables (expressed as mean±SD) and the χ^2^ tests or the Fisher’s exact tests for categorical variables (expressed as count and percentage). Secondary endpoints for intervention and control groups will be compared using χ^2^ test for binary outcomes, and t-test for continuous outcomes. For subgroup analyses, linear or logistic regression will be used to determine the effects of treatment allocation according to HF status. No adjustment or imputation will be used for missing data. For this feasibility study, we considered a sample of 20 participants enrolled as a minimum number acceptable for feasibility and a daily mean difference of 250 mL of fluid intake between treatment arms as clinically relevant. Progression to a multicentre clinical outcomes trial will be contingent on a series of stop/go criteria based on achieving the primary outcome targets and on secondary outcomes relating to data quality.

## Monitoring and evaluation

The trial will report serious adverse events (SAEs) that are attributable to the study through the EHR, in accordance with the safety reporting processes and sponsor policy.^27^ The Trial Management Group will review recruitment rates, SAEs and any substantial amendments to the protocol.

### Patient and public involvement

Patients were integrally involved in the design of the study as previously reported.^28^ In brief this involved a patient participation event where options for consent and outcome measures were explored which subsequently informed the study design. In accordance with the National Institute for Health Research guidance on coproduction,^29^ one participant (KC) served as a patient advisor to the trial and reviewed all trial materials.

### Ethics and dissemination

This study protocol was approved by the London Riverside Research Ethics Committee (Ref: 22/LO/0889) and sponsored by University College London (Ref: 151938). A verbal opt-out model of consent was adopted based on the negligible risk associated with the intervention. Patients are informed of their study participation and of their treatment allocation and can opt-out of the study or decline to follow the recommendation for oral fluid restriction at any time. A copy of the sample PIS is provided to patients (online supplemental appendix 5). The data management for the trial conforms to NHS Information standards DCB0129 and DCB0160 and was approved by University College London Hospitals NHS Foundation Trust (UCLH) Digital Services Safety board.^30^ The alert is not classified as a separate piece of software but is incorporated within the Epic EHR, which is registered as a class I medical device by the UK Medicines and Healthcare products Regulatory Agency.^31 32^ The study findings will be disseminated through publication in open-access peer-reviewed journals and through the provision of reports for patients and clinicians involved in the study. As a feasibility study, the findings shared will include trial evaluation, and whether alerts which ‘nudge’ clinicians to recruit patients into pragmatic, low-risk comparative effectiveness trials,^33^ are a scalable way to conduct efficient RCTs.^18^ The THIRST Alert feasibility study will share insights that inform the design and execution of future integrated approaches to evidence generation from routine care.

## Supplementary Material

Reviewer comments

Author's manuscript

## References

[1] Savarese G, Becher PM, Lund LH, et al. Global burden of heart failure: A comprehensive and updated review of epidemiology. Cardiovasc Res 2023;118:3272–87. 10.1093/cvr/cvac013 35150240

[2] McDonagh TA, Metra M, Adamo M, et al. ESC guidelines for the diagnosis and treatment of acute and chronic heart failure. Eur Heart J 2021;42:3599–726. 10.1093/eurheartj/ehab368 34447992

[3] Koratala A, Ronco C, Kazory A. Diagnosis of fluid overload: from conventional to contemporary concepts. Cardiorenal Med 2022;12:141–54. 10.1159/000526902 36096121

[4] Aliti GB, Rabelo ER, Clausell N, et al. Aggressive fluid and sodium restriction in acute decompensated heart failure: a randomized clinical trial. JAMA Intern Med 2013;173:1058–64. 10.1001/jamainternmed.2013.552 23689381

[5] Machado d’Almeida KS, Rabelo-Silva ER, Souza GC, et al. Aggressive fluid and sodium restriction in decompensated heart failure with preserved ejection fraction: results from a randomized clinical trial. Nutrition 2018;54:111–7. 10.1016/j.nut.2018.02.007 29793053

[6] Travers B, O’Loughlin C, Murphy NF, et al. Fluid restriction in the management of decompensated heart failure: no impact on time to clinical stability. J Card Fail 2007;13:128–32. 10.1016/j.cardfail.2006.10.012 17395053

[7] Sedgwick P, Greenwood N. Understanding the Hawthorne effect. BMJ 2015:h4672. 10.1136/bmj.h4672 26341898

[8] Heidenreich PA, Bozkurt B, Aguilar D, et al. ACC/AHA/HFSA guideline for the management of heart failure. Journal of Cardiac Failure 2022;28:e1–167. 10.1016/j.cardfail.2022.02.010 35378257

[9] Taylor CJ, Huntley AL, Burden J, et al. Research priorities in advanced heart failure: James LIND alliance priority setting partnership. Open Heart 2020;7:e001258. 10.1136/openhrt-2020-001258 32606070 PMC7328807

[10] Damman K, Voors AA, Hillege HL, et al. Congestion in chronic systolic heart failure is related to renal dysfunction and increased mortality. Eur J Heart Fail 2010;12:974–82. 10.1093/eurjhf/hfq118 20685688

[11] Naschitz JE, Slobodin G, Lewis RJ, et al. Heart diseases affecting the liver and liver diseases affecting the heart. Am Heart J 2000;140:111–20. 10.1067/mhj.2000.107177 10874271

[12] Aithal GP, Palaniyappan N, China L, et al. Guidelines on the management of Ascites in cirrhosis. Gut 2021;70:9–29. 10.1136/gutjnl-2020-321790 33067334 PMC7788190

[13] NICE guideline NG107. RRT and conservative management evidence review for dietary management and fluid restriction. n.d. Available: https://www.nice.org.uk/guidance/ng107/evidence/i-dietary-management-and-fluid-restriction-pdf-235524385620

[14] Nice guideline NG203. Chronic kidney disease: assessment and management. n.d. Available: https://www.nice.org.uk/guidance/ng203/resources/chronic-kidney-disease-assessment-and-management-pdf-66143713055173

[15] Boorsma EM, Ter Maaten JM, Damman K, et al. Congestion in heart failure: a contemporary look at physiology, diagnosis and treatment. Nat Rev Cardiol 2020;17:641–55. 10.1038/s41569-020-0379-7 32415147

[16] Gheorghiade M, Follath F, Ponikowski P, et al. Assessing and grading congestion in acute heart failure: a scientific statement from the acute heart failure committee of the heart failure Association of the European society of cardiology and endorsed by the European society of intensive care medicine. Eur J Heart Fail 2010;12:423–33. 10.1093/eurjhf/hfq045 20354029

[17] Agusti A, Bel E, Thomas M, et al. Treatable traits: toward precision medicine of chronic airway diseases. Eur Respir J 2016;47:410–9. 10.1183/13993003.01359-2015 26828055

[18] Usman MS, Van Spall HGC, Greene SJ, et al. The need for increased pragmatism in cardiovascular clinical trials. Nat Rev Cardiol 2022;19:737–50. 10.1038/s41569-022-00705-w 35581337 PMC9112643

[19] Eldridge SM, Lancaster GA, Campbell MJ, et al. Defining feasibility and pilot studies in preparation for randomised controlled trials: development of a conceptual framework. PLoS ONE 2016;11:e0150205. 10.1371/journal.pone.0150205 26978655 PMC4792418

[20] NHS England. Acute global Digital Exemplars. 2022. Available: https://www.england.nhs.uk/digitaltechnology/connecteddigitalsystems/exemplars/acute-global-digital-exemplars/ [Accessed 17 May 2022].

[21] Chan A-W, Tetzlaff JM, Altman DG, et al. SPIRIT 2013 statement: defining standard protocol items for clinical trials. Ann Intern Med 2013;158:200–7. 10.7326/0003-4819-158-3-201302050-00583 23295957 PMC5114123

[22] Bellary S, Krishnankutty B, Latha MS. Basics of case report form designing in clinical research. Perspect Clin Res 2014;5:159–66. 10.4103/2229-3485.140555 25276625 PMC4170533

[23] Skivington K, Matthews L, Simpson SA, et al. A new framework for developing and evaluating complex interventions: update of medical research Council guidance. BMJ 2021;374:n2061. 10.1136/bmj.n2061 34593508 PMC8482308

[24] Loudon K, Treweek S, Sullivan F, et al. The PRECIS-2 tool: designing trials that are fit for purpose. BMJ 2015;350:h2147. 10.1136/bmj.h2147 25956159

[25] Wilson FP, Martin M, Yamamoto Y, et al. Electronic health record alerts for acute kidney injury: multicenter, randomized clinical trial. BMJ 2021;372:m4786. 10.1136/bmj.m4786 33461986 PMC8034420

[26] Blatch-Jones AJ, Pek W, Kirkpatrick E, et al. Role of feasibility and pilot studies in randomised controlled trials: a cross-sectional study. BMJ Open 2018;8:e022233. 10.1136/bmjopen-2018-022233 PMC6169762 30257847

[27] NHS Health Research Authority. Safety and progress reports (other research) procedural table. Available: https://www.hra.nhs.uk/approvals-amendments/managing-your-approval/safety-reporting/safety-and-progress-reports-other-research-procedural-table/ [Accessed 10 May 2023].

[28] Chen Y, Hosin AA, George MJ, et al. Digital technology and patient and public involvement (PPI) in routine care and clinical research—A pilot study. PLoS ONE 2023;18:e0278260. 10.1371/journal.pone.0278260 36735724 PMC9897511

[29] Staniszewska S, Denegri S, Matthews R, et al. Reviewing progress in public involvement in NIHR research: developing and implementing a new vision for the future. BMJ Open 2018;8:e017124. 10.1136/bmjopen-2017-017124 PMC6067369 30061427

[30] NHS Digital. Dcb0160: clinical risk management: its application in the deployment and use of health IT systems. 2023. Available: https://digital.nhs.uk/data-and-information/information-standards/information-standards-and-data-collections-including-extractions/publications-and-notifications/standards-and-collections/dcb0160-clinical-risk-management-its-application-in-the-deployment-and-use-of-health-it-systems [Accessed 3 Feb 2023].

[31] Medicines and Healthcare products Regulatory Agency. Public access registration database. 2023. Available: https://pard.mhra.gov.uk/manufacturer-details/33914 [Accessed 2 Sep 2023].

[32] Medicines and Healthcare products Regulatory Agency. Guidance:medical Devicee stand-alone including Apps (including Ivdmds). 2023. Available: https://assets.publishing.service.gov.uk/government/uploads/system/uploads/attachment_data/file/1105233/Medical_device_stand-alone_software_including_apps.pdf [Accessed 3 Feb 2023].

[33] Chen Y, Harris S, Rogers Y, et al. Nudging within learning health systems: next generation decision support to improve cardiovascular care. Eur Heart J 2022;43:1296–306. 10.1093/eurheartj/ehac030 35139182 PMC8971005

