# Supplementary material for: Rationale and design of the THIRST Alert feasibility study: a pragmatic, single-centre, parallel-group randomised controlled trial of an interruptive alert for oral fluid restriction in patients treated with intravenous furosemide
Source: BMJ Open. 2024 Jan 12;14(1):e080410. doi: 10.1136/bmjopen-2023-080410 (PMC10806795; doi:10.1136/bmjopen-2023-080410)
Supplement: Supplementary data [file bmjopen-2023-080410supp001.pdf]

Appendix 1 – Fluid restriction at study site

Fluid restriction status	Total (%)	Primary diagnosis of Heart failure (%)	Heart failure as co-morbidity (%)	No diagnosis of Heart failure (%)
**Received fluid restriction at least once**	826 (56)	373 (71)	294 (55)	159 (38)
**Did not receive fluid restriction**	661 (44)	152 (29)	244 (45)	265 (62)

Table S1. Fluid restriction treatment strategy at the study site prior to trial implementation. Data shown is from unplanned admissions at University College Hospital between April 2019 – October 2022 where patients were prescribed IV furosemide within 48 hours of admission. The total number of patients was 1537 of data was available on heart failure and fluid restriction status in 1487.

Appendix 2 - Trial management group

Dr. Yang Chen (Principal Investigator) – Clinical Research Fellow & Cardiology Registrar

Dr. Tom Lumbers (Chief Investigator) – Associate Professor & Consultant Cardiologist

Dr. Anoop Shah – Associate Professor & Consultant Clinical Pharmacologist

Prof. Matthew Sydes m.sydes@ucl.ac.uk – Professor of Clinical Trials & Methodology

Prof. Folkert W. Asselbergs, f.asselbergs@ucl.ac.uk - Professor of Precision Medicine

Appendix 3 – Programme Theory for THIRST Alert

Event	Trigger	Action
**1.** Patient identification	Based on a *regular or continuous IV* furosemide prescription within 48 hours of unplanned admission	First alert is presented to prescribing clinician after triggering action is performed, most likely in emergency department or acute assessment unit
**2.** Alert Presentation	Based on trigger of 'Open Patient Chart' and 'Open Orders Sidebar'	Clinician accepts randomisation – patient enrolled and status on EHR updated to 'research participant' visible to all staff
		Clinician rejects randomisation – patient receives a non-randomised treatment recommendation for fluid restriction or no restriction, in keeping with usual care.
		Clinician defers – alert represented after another trigger action performed by any prescribing clinician within 48 hours
**3.** Alert recommendation	Based on clicking 'Yes' to first alert	Fluid restriction alert
		No Fluid restriction alert
**4.** Documented evidence of treatment allocation and communication to nursing staff and patient	Alert prompts user to link to document in patient notes.	Documentation of treatment allocation occurs in same manner as routine care. Enrolled patients have an additional workflow within the EHR for nursing staff to document fluid balance
	Alert prompts staff to provide additional participant information sheet	A paper PIS is available on all anticipated ward locations and a link supplied to print off a digital copy
**5.** Documentation of fluid intake, and other secondary trial outcomes: e.g., fluid balance, weight and patient experience	Prompted by nursing workflow, medical workflow and usual care processes	No additional changes to existing routine care processes
**6.** Patient is no longer fluid overloaded	Patient's clinical status has changed and their medical therapy has been de-escalated	No change to usual care processes with regards to continuation or cessation of fluid restriction arm. Trial ends at the end of the admission episode

Table S2. Programme theory for THIRST Alert trial. Steps 1-6 represent a summary of the logic model considered for the trial intervention and its downstream effects

Chen Y, *et al. BMJ Open* 2024; 14:e080410. doi: 10.1136/bmjopen-2023-080410

Appendix 4 – Alert Design

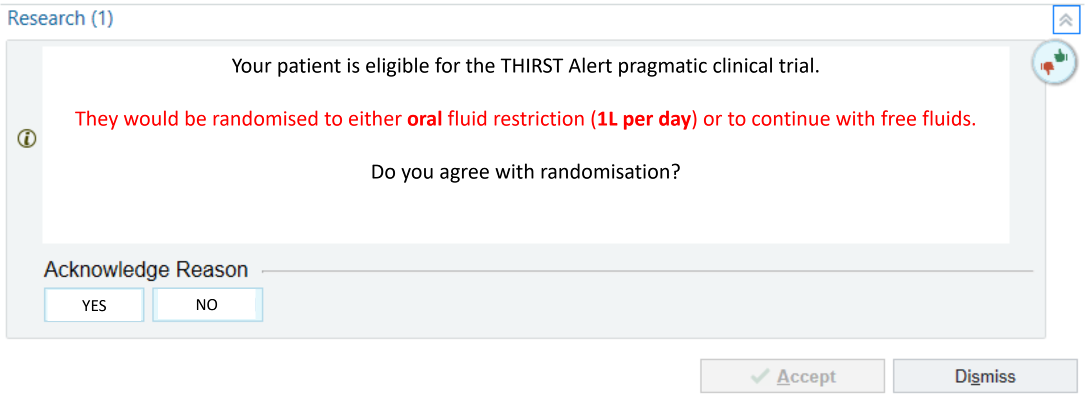

Supplemental Figure 1a. THIRST Alert invitation to enrol

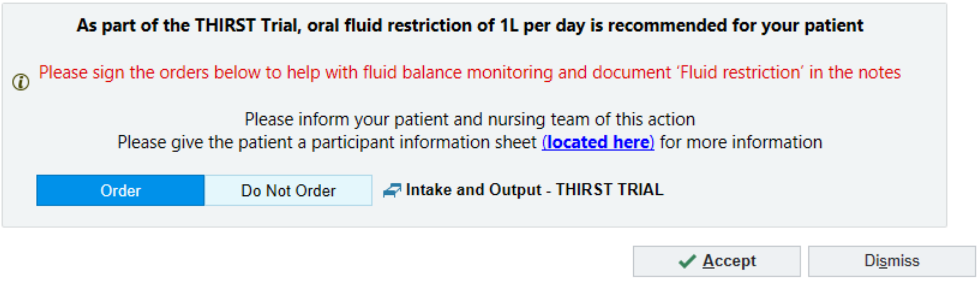

Supplemental Figure 1b. Oral fluid restriction recommendation

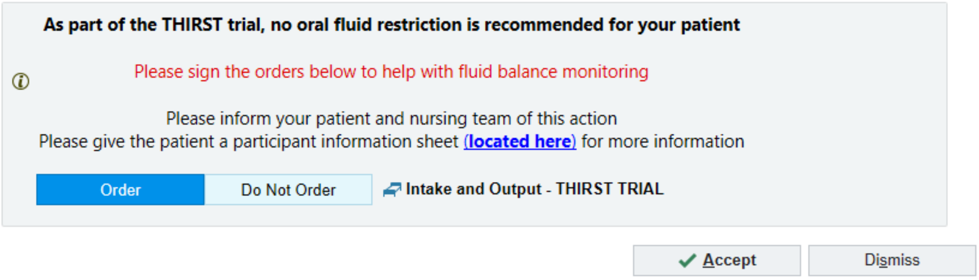

Supplemental Figure 1c. No oral fluid restriction recommendation

Chen Y, *et al. BMJ Open* 2024; 14:e080410. doi: 10.1136/bmjopen-2023-080410

Appendix 5 – Patient material

University College London Hospitals NHS
NHS Foundation Trust

THIRST Alert participant information sheet

THIRST Alert

We are inviting you to take part in a research project called the THIRST trial
We wish to find out if restricting the total daily oral fluid intake in patients treated for fluid overload has any additional benefit to prescribing medications for patients with your condition of fluid overload. The THIRST trial will help to answer this question by providing randomised recommendations on oral fluid intake or no oral fluid intake using the electronic health care record system that the doctors and nurses use to manage your care.

Why am I being asked to take part in this research?
Your medical team have initiated medication for the treatment of fluid overload in the body but they are uncertain as to whether or not oral fluid restriction would be of additional benefit. After agreeing to include you in the study, your team will have received a randomised recommendation to either: fluid restriction of no more than 1 litre per day or continuing with your usual amount of oral fluid intake.

Although fluid restriction is a commonly used treatment in your condition, its effectiveness in helping to relieve symptoms is unclear. The mainstay of treatment remains medications to help remove excess water and this is unaffected by whether you restrict fluid or not.

Although you are unlikely to receive any direct benefit from taking part, the results of the study may improve the management of patients with a similar condition in the future.

Do I have to take part?
You do not have to take part in the study; you have the right to withdraw at any time. Your decision will not affect the quality of care you receive.

What will I need to do if I take part?
You do not need to do anything as a result of taking part. There are no additional tests or appointments. As part of your usual care, you will be asked how you are feeling and how thirsty you are. There are no extra visits to your doctor over and above those needed for your normal care. The study will last only for the time you are in hospital. The normal processes for your discharge and follow up are unaffected by the study.

How do I opt out?
If you would not like to be included in this study, please let the doctors and nurses looking after you know or alternatively contact the study team directly.

What are the disadvantages/risks?
There are no extra risks involved in taking part in this research. If at any moment you feel like you would like to drink more fluids, you can ask your usual care team for more.

What will happen to information collected about me during the study?
Your medical information will be kept strictly confidential by your doctor. The researchers will only be given as much information from your medical records as is needed for this research and that information will be anonymised. They will not be given your name, where you live or anything that could identify you. The results of this research will be made available to all those taking part who would like to receive it.

Study Poster Version No. 1.0 ………… Date…03 May 2023…………
IRAS 313551

Who is organising and funding the research?
This study is being carried out by Dr Yang Chen, Dr Anoop Shah, Professor Folkert Asselbergs and Dr Tom Lumbers. It is sponsored by University College London and has support from the Clinical Research Informatics Unit of University College London Hospital (UCLH) and the Biomedical Research Centre at UCLH.

Further Information
You can ask your medical team any questions you may have about the study. You may also obtain more detailed information about this research, including how your medical information will be used, your privacy protected, and the compensation arrangements in the unlikely event that anything goes wrong from the following contacts:

UCLH Patient Advice & Liaison Service (PALS)
Address: PALS
Ground Floor Atrium
University College Hospital
235 Euston Road
London NW1 2BU
Telephone (main hospital): 02034473042
Email: Uclh.pals@nhs.net

Study team contact details:
Dr Yang Chen [Principal investigator: yang.chen@nhs.net]
Dr Tom Lumbers [Chief Investigator: tom.lumbers@nhs.net]
Cardiology Department at UCLH: 020 3447 8066

A description of the study is also available on the following UCLH website, and we plan to publish the study results there too: https://www.uclhospitals.brc.nihr.ac.uk/current-work

Thank you for reading this information.

Study Poster Version No. 1.0 ………… Date…03 May 2023…………
IRAS 313551

Pragmatic research during routine care at UCLH - THIRST Alert trial

A study of patients with fluid overload who may be suitable for fluid restriction

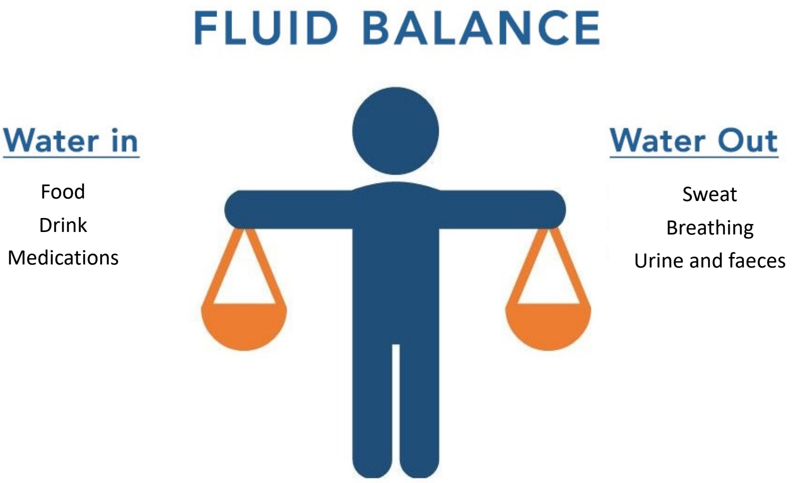

- Your medical team will inform you whether you are suitable for the study and if you will receive either (1) oral fluid restriction of up to 1 litre per day or (2) no restriction of oral fluids
- No other aspect of your care will be affected
- If you do not wish to take part, please let your clinical team know or contact the study team. Please see here for more information:

Study team and contact details:
Yang.chen@nhs.net - Clinical research fellow
Tom.lumbers@nhs.net - Consultant cardiologist
Cardiology Department at UCLH: 020 3447 8066

Study Poster Version No. 1.0 ………… Date…03 May 2023…………
IRAS 313551
