# Supplementary material for: Rationale and design of the THIRST Alert feasibility study: a pragmatic, single-centre, parallel-group randomised controlled trial of an interruptive alert for oral fluid restriction in patients treated with intravenous furosemide
Source: BMJ Open. 2024 Jan 12;14(1):e080410. doi: 10.1136/bmjopen-2023-080410 (PMC10806795; doi:10.1136/bmjopen-2023-080410)
Supplement: Author's manuscript [file bmjopen-2023-080410.draft_revisions.pdf]

BMJ Open is committed to open peer review. As part of this commitment we make the peer review history of every article we publish publicly available.

When an article is published we post the peer reviewers' comments and the authors' responses online. We also post the versions of the paper that were used during peer review. These are the versions that the peer review comments apply to.

The versions of the paper that follow are the versions that were submitted during the peer review process. They are not the versions of record or the final published versions. They should not be cited or distributed as the published version of this manuscript.

BMJ Open is an open access journal and the full, final, typeset and author-corrected version of record of the manuscript is available on our site with no access controls, subscription charges or pay-per-view fees (http://bmjopen.bmj.com).

If you have any questions on BMJ Open's open peer review process please email info.bmjopen@bmj.com

BMJ Open

# Rationale and design of THIRST Alert: a pragmatic, parallel group, randomised controlled trial of an interruptive alert for oral fluid restriction in patients treated with intravenous furosemide

| | |
|---|---|
| Journal: | *BMJ Open* |
| Manuscript ID | bmjopen-2023-080410 |
| Article Type: | Protocol |
| Date Submitted by the Author: | 29-Sep-2023 |
| Complete List of Authors: | Chen, Yang ; University College London, Institute of Health Informatics; University College London Hospitals NHS Foundation Trust, Clinical and Research Informatics Unit<br>Shah, Anoop; University College London, Institute of Health Informatics<br>Jani, Yogini; University College London Hospitals NHS Foundation Trust, Centre for Medicines Optimisation Research & Education - CMORE<br>Higgins, Daniel; University College London Hospitals NHS Foundation Trust, Clinical and Research Informatics Unit<br>Saleem, Nausheen; University College London Hospitals NHS Foundation Trust, Clinical and Research Informatics Unit<br>Chafer, Kris; University College London Hospitals NHS Foundation Trust, Clinical and Research Informatics Unit<br>Sydes, Matthew; Medical Research Council Clinical Trials Unit at University College London, MRC Clinical Trials Unit, Institute of Clinical Trials and Methodology; Health Data Research UK,<br>Asselbergs, Folkert; University College London, Institute of Health Informatics; University of Amsterdam, Department of Cardiology, Amsterdam University Medical Center<br>Lumbers, R Thomas; University College London, Institute of Health Informatics |
| Keywords: | Heart failure < CARDIOLOGY, Randomized Controlled Trial, Feasibility Studies, Electronic Health Records |
| | |

SCHOLARONE™
Manuscripts

# Rationale and design of THIRST Alert: a pragmatic, parallel group, randomised controlled trial of an interruptive alert for oral fluid restriction in patients treated with intravenous furosemide

Yang Chen[1,2]
Anoop D. Shah[1,2,]
Yogini Jani[4]
Daniel Higgins[2]
Nausheen Saleem[2]
Kris Chafer[2]
Matt Sydes[5,6]
Folkert W Asselbergs[1,4]
Tom Lumbers[1]

1. Institute of Health Informatics, Faculty of Population Health Sciences, University College London, UK
2. Clinical and Research Informatics Unit, NIHR Biomedical Research Centre at University College London Hospitals, UK
3. Centre for Medicines Optimisation Research and Education, University College London Hospitals NHS Foundation Trust, London, UK
4. Department of Cardiology, Amsterdam University Medical Centre, University of Amsterdam, Amsterdam, The Netherlands
5. MRC Clinical Trials Unit, University College London, UK
6. Health Data Research UK, London, UK NW1 2BE

**Corresponding author:** Tom Lumbers

**E-mail:** t.lumbers@ucl.ac.uk

**Word count (**exc table/ figures/ legends/ acknowledgements**): 2189**

For peer review only

# Abstract

**Introduction**

Acute heart failure (HF) is a major cause of unplanned hospitalisation characterised by excess body water. A restriction in oral fluid intake is commonly imposed on patients as an adjunct to pharmacological therapy with loop diuretics, but there is a lack of evidence from traditional randomised controlled trials (RCTs) to support the safety and effectiveness of this intervention in the acute setting.

This study aims to explore the feasibility of using computer alerts within the electronic health record (EHR) system to invite clinical care teams to enrol patients into a pragmatic RCT at the time of clinical decision-making. It will additionally assess the effectiveness of using an alert to help address the clinical research question of whether oral fluid restriction is a safe and effective adjunct to pharmacological therapy for patients admitted with fluid overload.

**Methods and analysis**

THIRST Alert is a single centre, parallel group, open-label pragmatic RCT embedded in the EHR system that will be conducted as a feasibility study. The clinical care team will be invited to enrol suitable patients in the study using a point-of-care alert. Enrolled patients will then be randomised to either restricted or unrestricted oral fluid intake. Two primary outcomes will be explored: (1) the proportion of eligible patients enrolled in the study and (2) the mean difference in oral fluid intake between randomised groups. A series of secondary outcomes are specified to evaluate the effectiveness of the alert, adherence to

For peer review only

the randomised treatment allocation, and the quality of data generated from routine care, relevant to the outcomes of interest.

**Ethics and dissemination**

This study was approved by Riverside Research Ethics Committee (Ref: 22/LO/0889) and will be published on completion. Trial registration number NCT05869656.

**Keywords**

*Embedded trials; point-of-care randomisation; fluid overload; fluid restriction; heart failure*

**Strengths and Limitations of this study**

- THIRST Alert is a pragmatic randomised controlled trial where all elements of trial conduct are embedded within the routine care clinical pathway
- THIRST Alert has been co-designed with a multidisciplinary team to facilitate its implementation at the study site
- THIRST Alert is evaluating the feasibility of using an interruptive alert to recruit patients into a trial which aims to generate evidence in an area lacking robust trial data
- A proportionate consent model has been used to help with the practicality of trial conduct
- Uncertainty remains regarding EHR data quality and the suitability of deploying a pragmatic RCT to conduct comparative effectiveness research of existing interventions used in acute care

For peer review only

# Introduction

Acute heart failure (HF) is an important cause of unplanned hospitalisation and is characterised by excess body water, also known as congestion. (1, 2) Despite being a common clinical problem, there is a lack of high-quality evidence to guide drug treatment and non-pharmacological measures. (3) Restriction of oral fluid intake is commonly used as an adjunct to pharmacological therapy but its effectiveness is uncertain and may exacerbate symptoms of thirst, as suggested by the limited randomised controlled trials which have been conducted to date (4–6). These previous trials achieved only small differences in oral fluid intake, which may reflect the Hawthorne effect whereby participation in a clinical trial influences fluid intake in both groups. (7)

Patients and clinicians identify the need for better treatment of fluid overload as a key priority, and the use of oral fluid restriction is also highlighted a gap in evidence in clinical practice guidelines (2, 8) (9). To address this evidence gap and some of the challenges encountered in previous studies, we designed a pragmatic RCT embedded in the electronic health record (EHR) system to evaluate the safety and effectiveness of oral fluid restriction in acute HF.

## Patient population

Fluid overload may be caused by liver or kidney dysfunction (10, 11), as well as by heart failure, with similar evidence gaps for congestion management in such circumstances. (12) (13) (14). However, a reliance on evaluating interventions for the immediate management of fluid overload only where the underlying cause is established may impact the

For peer review only - http://bmjopen.bmj.com/site/about/guidelines.xhtml

generalisability of any findings. Given that standardised clinical scores for congestion are not well validated or adopted in practice(15, 16), we used the prescription of two consecutive doses of intravenous furosemide within the first 48 hours of admission as evidence of a physician-assessed ‘treatable trait’ of fluid congestion, to define the study population. (17) At our proposed study site, more than one consecutive dose of intravenous furosemide was administered during the first 48 hours of admission in 1 in 25 of unplanned admissions between April 2019 to April 2022. 84% of these treatment episodes were subsequently coded for HF in Hospital Event Statistics and in 56%, oral fluid restriction was recommended (supplemental material). The use of prescribing intention to identify the study population enabled digital enrolment into the study based on a discrete triggering event. To our knowledge, there has been no previous RCT conducted in all patients treated for congestion with intravenous furosemide in the acute care setting.

### Pragmatic research

There are many evidence gaps in routine clinical practice that have not been addressed by conventional randomised controlled trials which may be prohibitively expensive. Pragmatic clinical trials (PCT) embedded in EHRs offer a potential solution, particularly relevant to the evaluation of low-risk interventions where clinical equipoise may exist. By integrating a randomisation procedure into routine care pathways, automating case identification through the EHR, engaging the clinical care team for patient recruitment, and using routinely collected data for trial outcomes, there is the potential to perform large-scale trials at low-cost and low burden to patients and their caregivers. Many questions remain however, about how best to deliver PCTs embedded in EHR systems, including the design of physician-facing alerts and different models of consent. For our study we employ an

For peer review only

interruptive alert to provide the regular care team with an invitation to enrol eligible patients, and use a proportionate process for consent that can be delivered by the same team.

The THIRST Alert trial aims to examine (1) whether interruptive alerts are an effective means to engage the usual care team in enrolling patients in an interventional pragmatic RCT; and (2), for enrolled patients, whether the randomised assignment to either oral fluid restriction to 1-litre per day or no oral fluid restriction leads to a difference in documented oral fluid intake. The evidence generated will be used to inform the design of subsequent multi-centre outcomes driven pragmatic clinical trials embedded in EHR systems.

For peer review only

# Methods and analysis

## Study design

THIRST Alert is a single centre, parallel group, open-label pragmatic RCT embedded in the EHR system that will be conducted as a feasibility study.(18) The study will be delivered entirely at University College Hospital, UCLH, a digitally mature hospital. (19) Given the low risk of the intervention, a model of verbal, opt-out consent is used. The trial is registered at ClinicalTrials.gov [NCT05869656]. Enrolment started in May 2023 and the estimated trial completion date is December 2023. The study protocol has been prepared with reference to the SPIRIT statement (20). An overview of the study procedures is given in **Figure 1**.

## Study procedures

The trial was design to be integrated into the electronic health record system EPIC (Epic, Epic Systems, Verona, WI). The screening and identification of eligible patients, display of interruptive alerts to physicians, randomisation and outcome ascertainment are all provisioned within EPIC. (22) Education events, internal communication and email correspondence were delivered to members of the routine care team to raise awareness of the study but no formal co-interventions were undertaken. The conduct and analysis of the trial is overseen by a trial management group who conceived a programme theory for the alert with relevant multidisciplinary input (see supplemental materials). The enrolment invitation and randomised treatment recommendations are classified as complex interventions according to the Medical Research Council framework. (23)

For peer review only

## Study participants

Adults of 18 years or older who were administered more than one dose of intravenous (IV) furosemide within the first 48 hours of an unplanned admission were eligible for the study. Patients admitted to a surgical, maternity or critical care ward were excluded **(Table 1)**.

## The interruptive alert inviting usual care team to enrol eligible patients

Any member of the clinical team may receive an interruptive alert (**supplemental figure S1**) during their interaction with EPIC for an eligible patient, informing them of the study and inviting them to enrol the patient. The clinician can click the appropriate button on the alert to enrol, decline to enrol, or defer the decision regarding enrolment. The alert is non-coercive, and the decision about patient enrolment is at the discretion of the treating clinician. We anticipate that physicians will enrol patients in cases where they judge that there is clinical equipoise as to whether oral fluid restriction is likely to benefit the patient. The alert was designed and tested with EPIC application specialists in an iterative manner, integrating feedback from end-users on the wording of the design.

## The randomised treatment intervention

After enrolment of patients in the study, the routine care team receives a recommendation regarding oral fluid intake and assignment is determined by point of care randomisation. For patients allocated to the intervention group, the routine care team receive a recommendation for a target oral fluid restriction of 1 litre per day; for patients allocated to the control group, the care team receive a recommendation for unrestricted oral fluid intake (supplemental figure S2). The oral fluid restriction target was chosen based on previous RCTs and acceptability in clinical practice (4–6). The randomised treatment

For peer review only

recommendation for both groups additionally advises the clinician to: (1) complete an EPIC order for fluid balance monitoring; (2) document the treatment allocation in the clinical notes (3) inform the patient and the nursing team and (4) provide the patient with a participant information sheet.

## Randomisation and blinding

Subjects will be randomised 1:1 using an internal random number rule implemented using a standard EPIC randomisation tool used in other PCTs.(24) No block randomisation or additional covariate balancing will be undertaken. The study is open label with patients and their care teams being made aware of the treatment allocation.

## Study Outcomes

### Primary outcomes

1. Number of eligible patients randomised
2. Mean difference in oral fluid intake for 48 hours post randomisation

### Secondary outcomes

1. Proportion of alerts actioned by a corresponding clinical order
2. Proportion of clinical orders actioned by nursing staff
3. Oral fluid intake per day
4. Net fluid balance per day
5. Daily weight difference between groups during the hospital admission
6. Weight change at 48 hours after randomisation
7. Length of inpatient stay
8. Frequency of blood test measurement
9. Dose and duration of diuretic medications
10. Daily change in Creatinine 48 hours after randomisation
11. Patient Reported Outcome Measures – subjective breathlessness, global wellbeing and thirst perception on a visual analogue scale.

These outcomes were chosen based on clinical relevance and the ability to detect the possibility of attributable harm during an inpatient admission.

For peer review only

## Sample size and power calculation

No formal sample size calculation was performed given the primary outcomes pertain to feasibility.

## Data collection, curation and storage

Baseline characteristics and study outcomes will be extracted from the routine care record. Information on the frequency of the interruptive alert and staff responses to alerts will be recorded in EPIC. We will also extract primary and secondary ICD-10 diagnosis codes assigned to the clinical episode by the clinical coding team. Study data will be transferred to a secure research environment within UCLH NHS Foundation Trust as pseudo-anonymised electronic Case Report Forms (eCRFs). No data will leave the UCLH NHS system. Patients who opt out or withdraw from the trial will have their decision documented on the eCRF and their data will not be included in the main analysis.

## Statistical analysis plan

Baseline characteristics of study participants will be summarised using percentages or means and standard deviations as appropriate and compared using t tests for continuous variables (expressed as mean ± standard deviation) and the $\chi^2$ tests or the Fisher's exact tests for categorical variables (expressed as count and percentage). Secondary endpoints for intervention and control groups will be compared using $\chi^2$ test for binary outcomes, and T-test for continuous outcomes. For subgroup analyses, linear or logistic regression will be used to determine the effects of treatment allocation according to heart failure status. No adjustment or imputation will be used for missing data. For this feasibility study, we aim to

For peer review only - http://bmjopen.bmj.com/site/about/guidelines.xhtml

recruit 20 participants over a period of 3 months. We consider a minimum daily mean difference of 250ml of fluid intake to be clinically relevant. Progression to a multi-centre clinical outcomes trial will be contingent on a series of stop/ go criteria based on achieving the primary outcome targets and on secondary outcomes relating to data quality.

### Monitoring and Evaluation

The trial will report serious adverse events (SAEs) that are attributable to the study through the EHR, in accordance with the non-CTIMP safety report process and sponsor policy. (25) The Trial Management Group will review recruitment rates, SAEs and any substantial amendments to the protocol..

### Patient and public involvement (PPI)

Patients were integrally involved in the design of the study as previously reported (26). In brief this involved a patient participation event where options for consent and outcome measures were explored which subsequently informed the study design. In accordance with the National Institute for Health Research guidance on co-production(27), one participant (KC) served as a patient advisor to the trial and reviewed all trial materials.

## Ethics and Dissemination

This study protocol was approved by the London Riverside Research Ethics Committee (Ref: 22/LO/0889) and sponsored by University College London (Ref: 151938). A verbal opt-out model of consent was adopted based on the negligible risk associated with the intervention.

For peer review only - http://bmjopen.bmj.com/site/about/guidelines.xhtml

Patients are informed of their study participation and of their treatment allocation and can opt-out of the study or decline to follow the recommendation for oral fluid restriction at any time. A copy of the sample participant information sheet (PIS) is provided to patients (**supplemental material**). The data management for the trial conforms to NHS Information standards DCB0129 and DCB0160 and was approved by the UCLH Digital Services Safety board. (28) The alert is not classified as a separate piece of software but is incorporated within the Epic electronic health record, which is registered as a Class I medical device by the UK Medicines and Healthcare products Regulatory Agency. (29)(30) The study findings will be disseminated through publication in open-access peer-reviewed journals and through the provision of reports for patients and clinicians involved in the study.

For peer review only

# Discussion

Pragmatic RCTs embedded in EHR systems have the potential to enable evidence generation during routine care, helping to meet the demand to conduct more efficient and representative trials in cardiovascular research.(31) Through examining the comparative effectiveness of existing interventions that have demonstrable variation in practice, many evidence gaps may be addressed. One such gap is the use of oral fluid restriction in patients who are treated pharmacologically for fluid overload in the setting of acute unplanned care. This protocol for THIRST Alert, a feasibility study, explores whether a new model of patient enrolment based on interruptive alerts to the routine care team is a suitable method to conduct a pragmatic RCT. The trial is designed to maximise the generalisability and applicability of the evidence generated and is highly pragmatic according to the PRECIS-2

framework (**Figure 2**). (32) Electronic health record system alerts which 'nudge' clinicians to recruit patients into pragmatic comparative effectiveness trials (33), could become a potentially scalable way to conduct efficient RCTs of low-risk existing therapies.(31) The THIRST Alert feasibility study will provide insight that inform the design and execution of this innovative approach to evidence generation, to inform better treatments for patients.

For peer review only

## Author contributions

YC, AS, MS, FA, and TL conceived the study idea. YC and TL wrote the first draft of the protocol. YJ, DH and NS helped to design, test and review the study alert. KC reviewed and edited the patient-facing study materials. All authors reviewed the manuscript and contributed to revisions.

## Acknowledgements

We are grateful to clinical colleagues, Inaki Elizondo, Pamela Stephenson, Sian Roberts-Walsh, Alexander Tranter and Ali Hosin for assistance with the study design. We thank Steve Harris and Pier Lambiase for comments on the study protocol and to members of the digital safety committee with the safe implementation of this study.

## Funding statement

This work was supported by the National Institute for Health and Care Research (NIHR) UCLH Biomedical Research Centre (BRC), award number NIHR-INF-1873. The study funder did not play any role in the design of the study.

## Conflict of interests

None declared

For peer review only

# References

1. Savarese G, Becher PM, Lund LH, Seferovic P, Rosano GMC, Coats A. Global burden of heart failure: A comprehensive and updated review of epidemiology. Cardiovasc Res. 2022; cvac013.

2. McDonagh TA, Metra M, Adamo M, Gardner RS, Baumbach A, Böhm M, et al. 2021 ESC Guidelines for the diagnosis and treatment of acute and chronic heart failure. European Heart Journal. 2021; 42(36):3599–726.

3. Koratala A, Ronco C, Kazory A. Diagnosis of Fluid Overload: From Conventional to Contemporary Concepts. Cardiorenal Med. 2022; 12(4):141–54.

4. Aliti GB, Rabelo ER, Clausell N, Rohde LE, Biolo A, Beck-da-Silva L. Aggressive fluid and sodium restriction in acute decompensated heart failure: a randomized clinical trial. JAMA Intern Med. 2013; 173(12):1058–64.

5. Machado d'Almeida KS, Rabelo-Silva ER, Souza GC, Trojahn MM, Santin Barilli SL, Aliti G, et al. Aggressive fluid and sodium restriction in decompensated heart failure with preserved ejection fraction: Results from a randomized clinical trial. Nutrition. 2018; 54:111–7.

6. Travers B, O'Loughlin C, Murphy NF, Ryder M, Conlon C, Ledwidge M, et al. Fluid restriction in the management of decompensated heart failure: no impact on time to clinical stability. J Card Fail. 2007; 13(2):128–32.

7. Sedgwick P, Greenwood N. Understanding the Hawthorne effect. BMJ. 2015; h4672.

8. Heidenreich PA, Bozkurt B, Aguilar D, Allen LA, Byun JJ, Colvin MM, et al. 2022 ACC/AHA/HFSA Guideline for the Management of Heart Failure. Journal of Cardiac Failure. 2022; 28(5):e1–167.

9. Taylor CJ, Huntley AL, Burden J, Gadoud A, Gronlund T, Jones NR, et al. Research priorities in advanced heart failure: James Lind alliance priority setting partnership. Open Heart. 2020; 7(1):e001258.

10. Damman K, Voors AA, Hillege HL, Navis G, Lechat P, Van Veldhuisen DJ, et al. Congestion in chronic systolic heart failure is related to renal dysfunction and increased mortality. European Journal of Heart Failure. 2010; 12(9):974–82.

11. Naschitz JE, Slobodin G, Lewis RJ, Zuckerman E, Yeshurun D. Heart diseases affecting the liver and liver diseases affecting the heart. American Heart Journal. 2000; 140(1):111–20.

12. Aithal GP, Palaniyappan N, China L, Härmälä S, Macken L, Ryan JM, et al. Guidelines on the management of ascites in cirrhosis. Gut. 2021; 70(1):9–29.

13. NICE guideline NG107. RRT and conservative management Evidence review for dietary management and fluid restriction [Internet]. [date unknown]. Available from:

For peer review only

https://www.nice.org.uk/guidance/ng107/evidence/i-dietary-management-and-fluid-restriction-pdf-235524385620.

14. Nice guideline NG203. Chronic kidney disease: assessment and management [Internet]. [date unknown]. Available from: https://www.nice.org.uk/guidance/ng203/resources/chronic-kidney-disease-assessment-and-management-pdf-66143713055173.

15. Boorsma EM, Maaten JM ter, Damman K, Dinh W, Gustafsson F, Goldsmith S, et al. Congestion in heart failure: a contemporary look at physiology, diagnosis and treatment. Nat Rev Cardiol. 2020; 17(10):641–55.

16. Gheorghiade M, Follath F, Ponikowski P, Barsuk JH, Blair JEA, Cleland JG, et al. Assessing and grading congestion in acute heart failure: a scientific statement from the acute heart failure committee of the heart failure association of the European Society of Cardiology and endorsed by the European Society of Intensive Care Medicine. Eur J Heart Fail. 2010; 12(5):423–33.

17. Agusti A, Bel E, Thomas M, Vogelmeier C, Brusselle G, Holgate S, et al. Treatable traits: toward precision medicine of chronic airway diseases. Eur Respir J. 2016; 47(2):410–9.

18. Eldridge SM, Lancaster GA, Campbell MJ, Thabane L, Hopewell S, Coleman CL, et al. Defining Feasibility and Pilot Studies in Preparation for Randomised Controlled Trials: Development of a Conceptual Framework. PLoS ONE. 2016; 11(3):e0150205.

19. NHS England. Acute global digital exemplars. (online) [Internet]. 2022 [cited 2022 May 17]. Available from: https://www.england.nhs.uk/digitaltechnology/connecteddigitalsystems/exemplars/acute-global-digital-exemplars/.

20. Chan A-W, Tetzlaff JM, Altman DG, Laupacis A, Gøtzsche PC, Krleža-Jerić K, et al. SPIRIT 2013 statement: defining standard protocol items for clinical trials. Ann Intern Med. 2013; 158(3):200–7.

21. Kotecha D, Asselbergs FW, Achenbach S, Anker SD, Atar D, Baigent C, et al. CODE-EHR best practice framework for the use of structured electronic healthcare records in clinical research. BMJ. 2022; e069048.

22. Latha M, Bellary S, Krishnankutty B. Basics of case report form designing in clinical research. Perspect Clin Res. 2014; 5(4):159.

23. Skivington K, Matthews L, Simpson SA, Craig P, Baird J, Blazeby JM, et al. A new framework for developing and evaluating complex interventions: update of Medical Research Council guidance. BMJ. 2021; n2061.

24. Wilson FP, Martin M, Yamamoto Y, Partridge C, Moreira E, Arora T, et al. Electronic health record alerts for acute kidney injury: multicenter, randomized clinical trial. BMJ. England; 2021; 372(8900488, bmj, 101090866):m4786.

25. NHS Health Research Authority. Safety and progress reports (other research) procedural table. (Online). [Internet]. [cited 2023 May 10]. Available from:

For peer review only

https://www.hra.nhs.uk/approvals-amendments/managing-your-approval/safety-reporting/safety-and-progress-reports-other-research-procedural-table/.

26. Chen Y, Hosin AA, George MJ, Asselbergs FW, Shah AD. Digital technology and patient and public involvement (PPI) in routine care and clinical research—A pilot study. PLoS ONE. 2023; 18(2):e0278260.

27. Staniszewska S, Denegri S, Matthews R, Minogue V. Reviewing progress in public involvement in NIHR research: developing and implementing a new vision for the future. BMJ Open. 2018; 8(7):e017124.

28. NHS Digital. DCB0160: Clinical Risk Management: its Application in the Deployment and Use of Health IT Systems [Internet]. 2023 [cited 2023 Feb 3]. Available from: https://digital.nhs.uk/data-and-information/information-standards/information-standards-and-data-collections-including-extractions/publications-and-notifications/standards-and-collections/dcb0160-clinical-risk-management-its-application-in-the-deployment-and-use-of-health-it-systems.

29. Medicines and Healthcare products Regulatory Agency. Guidance:Medical devicee stand-alone including apps (including IVDMDs) [Internet]. 2023 [cited 2023 Feb 3]. Available from: https://assets.publishing.service.gov.uk/government/uploads/system/uploads/attachment_data/file/1105233/Medical_device_stand-alone_software_including_apps.pdf.

30. Medicines and Healthcare products Regulatory Agency. Public Access Registration Database [Internet]. 2023 [cited 2023 Sep 27]. Available from: (https://pard.mhra.gov.uk/manufacturer-details/33914.

31. Usman MS, Van Spall HGC, Greene SJ, Pandey A, McGuire DK, Ali ZA, et al. The need for increased pragmatism in cardiovascular clinical trials. Nat Rev Cardiol [Internet]. 2022 [cited 2022 Sep 1]. Available from: https://www.nature.com/articles/s41569-022-00705-w.

32. Loudon K, Treweek S, Sullivan F, Donnan P, Thorpe KE, Zwarenstein M. The PRECIS-2 tool: designing trials that are fit for purpose. BMJ. 2015; 350:h2147.

33. Chen Y, Harris S, Rogers Y, Ahmad T, Asselbergs FW. Nudging within learning health systems: next generation decision support to improve cardiovascular care. Eur Heart J. 2022; 43(13):1296–306.

For peer review only - http://bmjopen.bmj.com/site/about/guidelines.xhtml

## DISPLAYS

| Study participants | Eligibility Criteria |
|---|---|
| Patients | **Inclusion**<br>- Aged 18 years and over<br>- Prescribed IV furosemide during the first 48h of their admission as a regular prescription rather than a one-off dose.<br>- Assessed as being suitable for inclusion in the trial by the responsible clinical team i.e., fluid restriction deemed to be in equipoise.<br>**Exclusion**<br>- Surgical or obstetric wards<br>- Critical care wards |
| Prescribing clinicians: Physicians (Consultants and Junior Doctors) | Any clinician with prescribing rights and who prescribes more than one dose of IV furosemide within 48 hours of admission, during the trial recruitment period. |
| Nursing staff | A separate order is triggered for enrolled patients and only presented to nursing staff who access their patient record to help facilitate fluid balance documentation |

**Table 1.** Eligibility criteria for THIRST Alert study participants

For peer review only

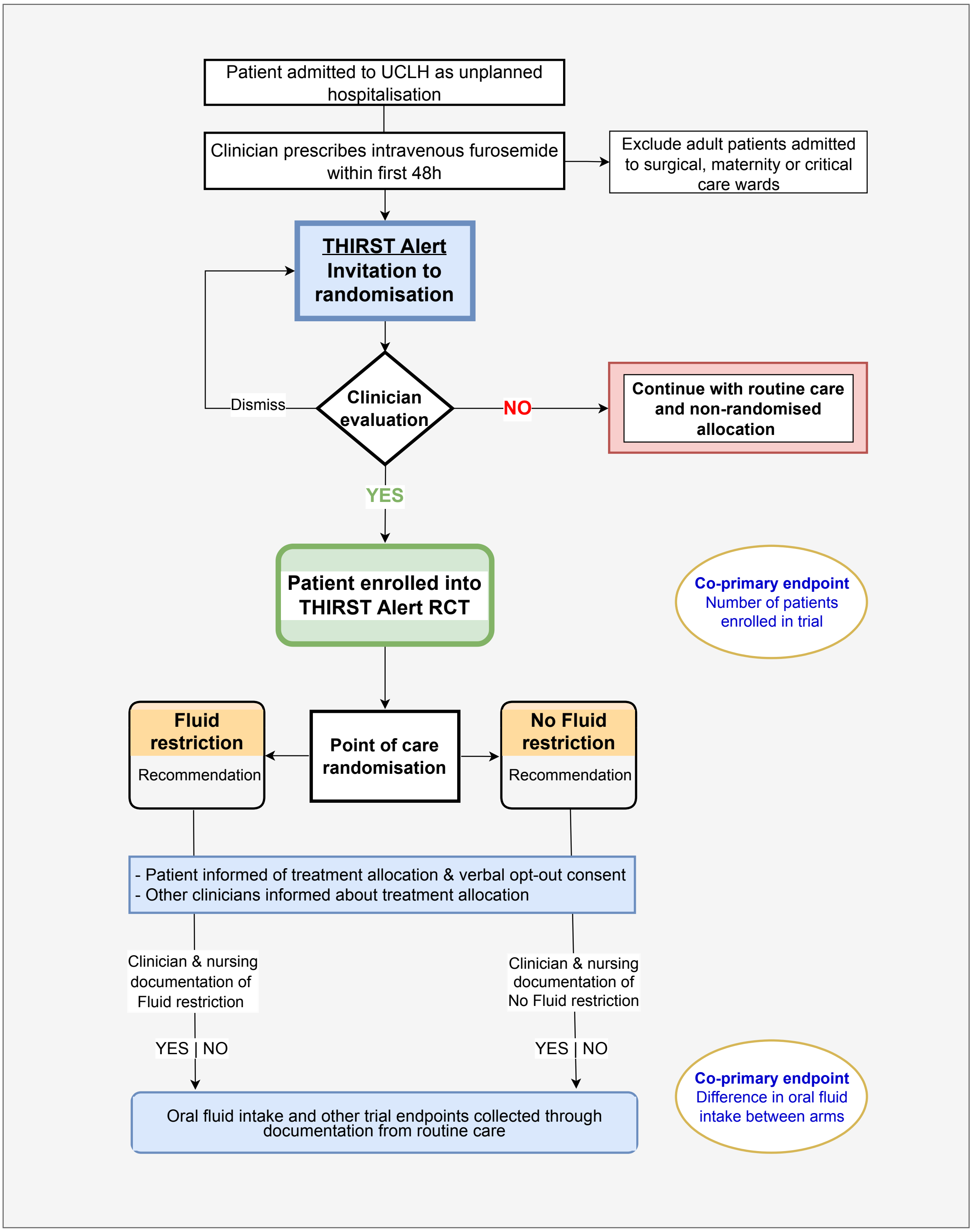


**Figure 1.** THIRST Alert trial patient flow

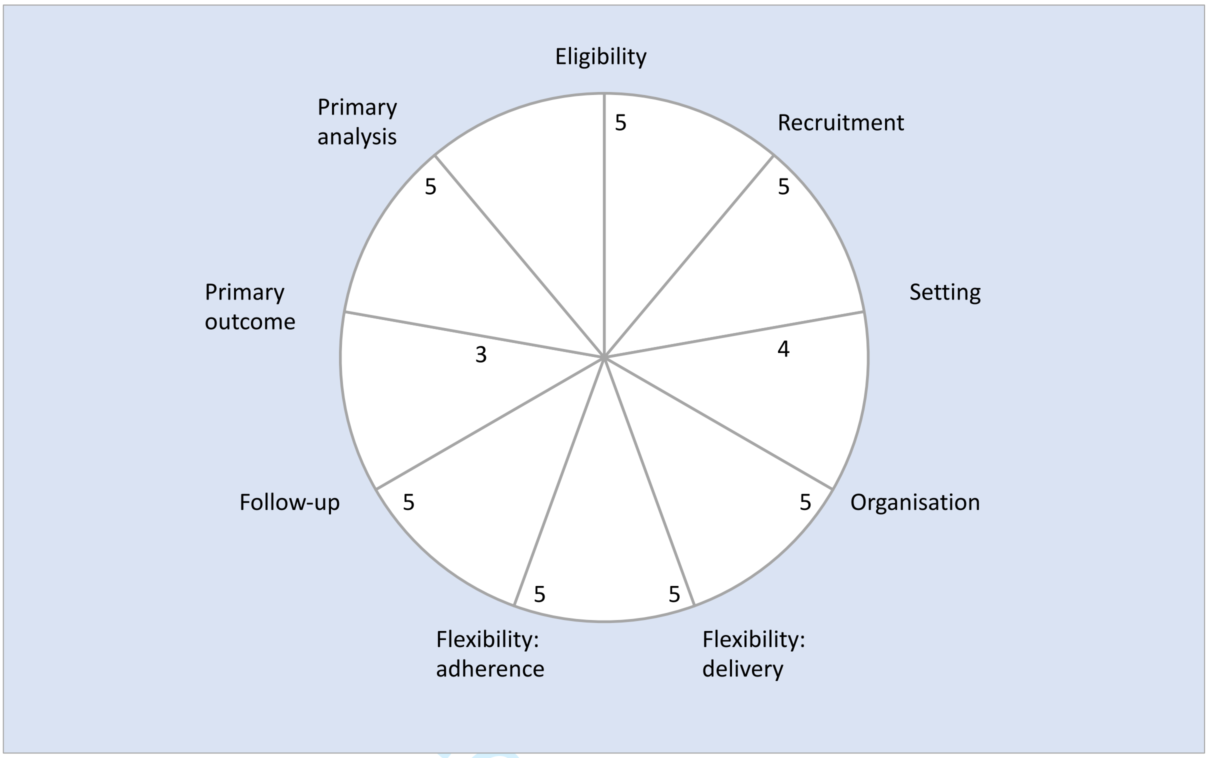


**Figure 2.** PRECIS-2 score for THIRST Alert trial. Each of the nine domains are scored on a 5-point Likert continuum (from 1=very explanatory “ideal conditions” to 5=very pragmatic “usual care conditions”)

For peer review only - http://bmjopen.bmj.com/site/about/guidelines.xhtml

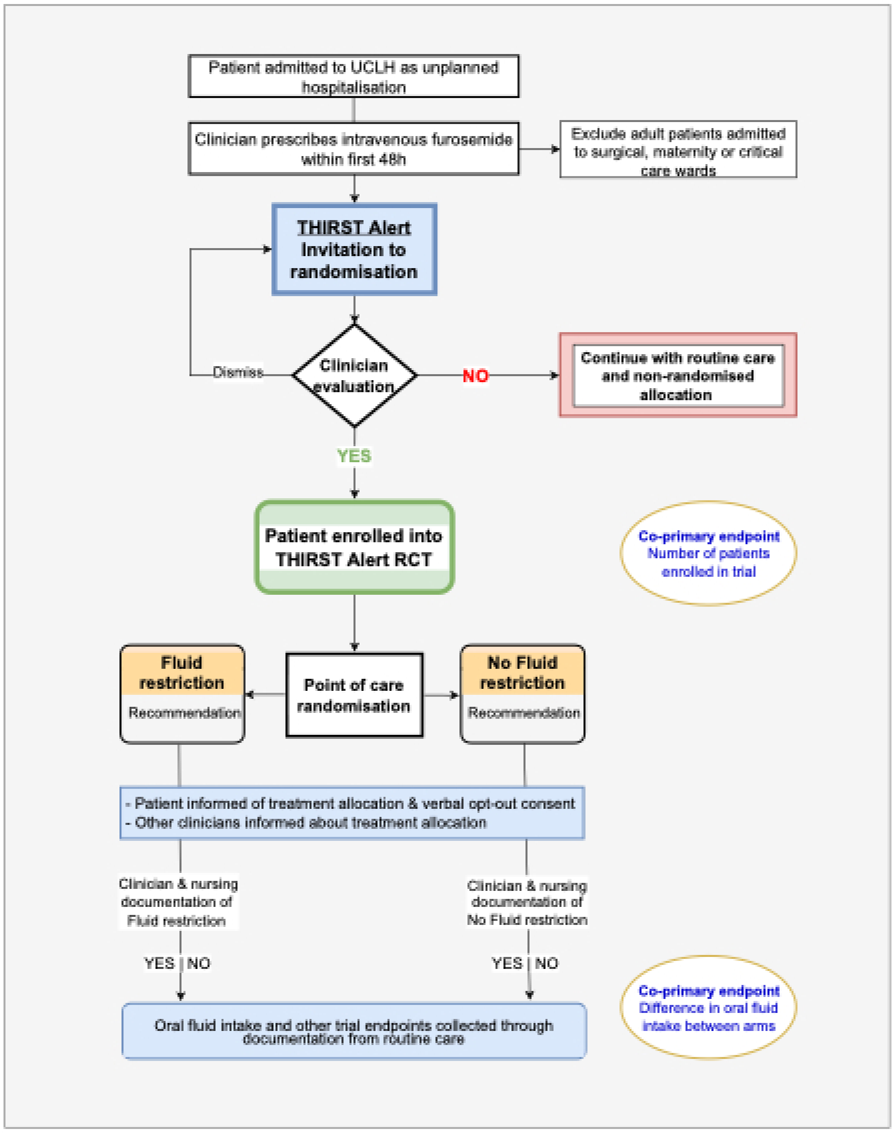


Figure 1. THIRST Alert trial patient flow

159x200mm (72 x 72 DPI)

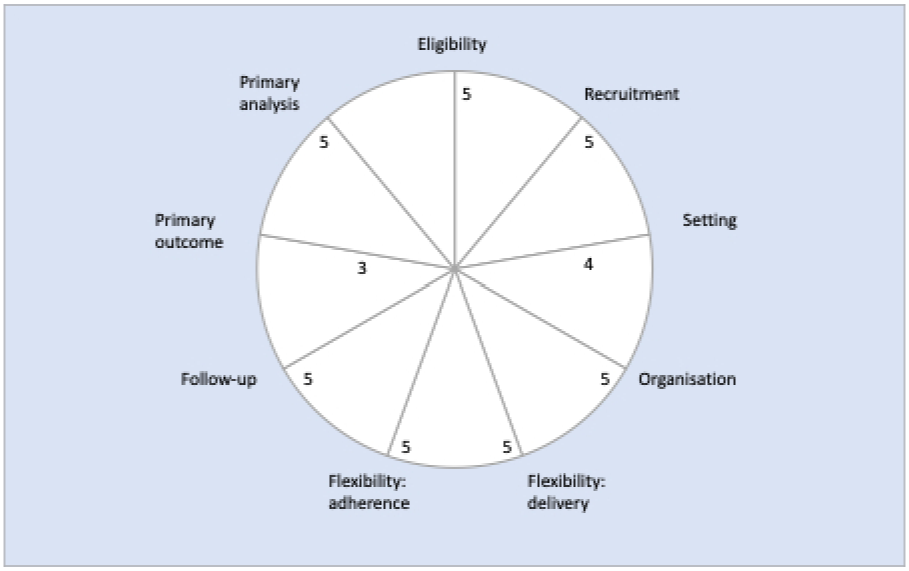


Figure 2. PRECIS-2 score for THIRST Alert trial. Each of the nine domains are scored on a 5-point Likert continuum (from 1=very explanatory “ideal conditions” to 5=very pragmatic “usual care conditions”)

159x99mm (72 x 72 DPI)

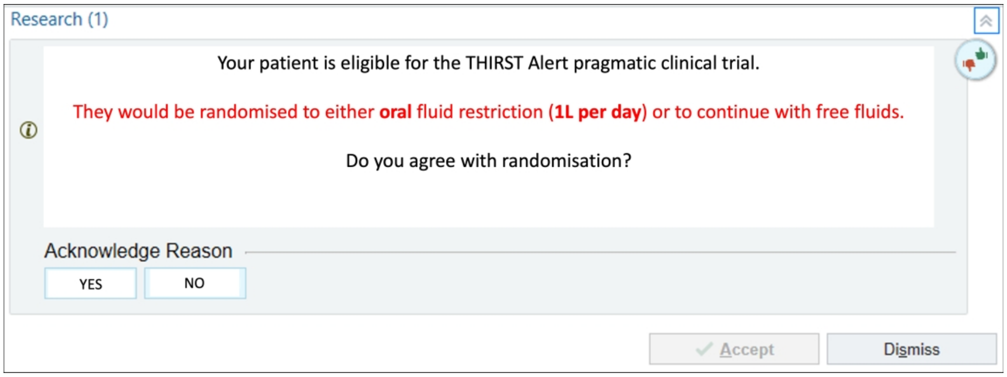


Supplemental Figure 1a. THIRST Alert invitation to enrol

311x114mm (300 x 300 DPI)

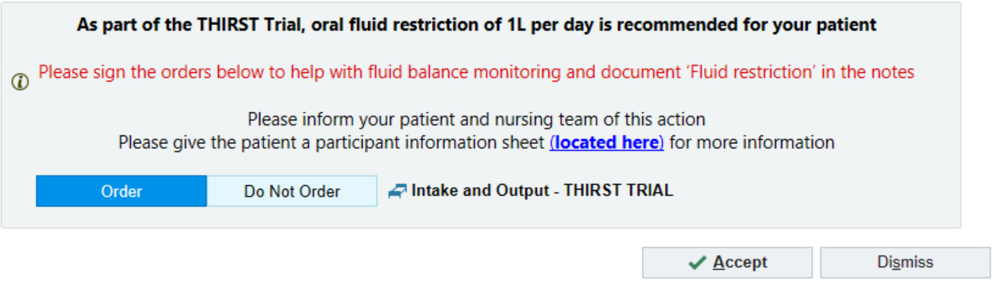


Supplemental Figure 1b. Oral fluid restriction recommendation

548x157mm (38 x 38 DPI)

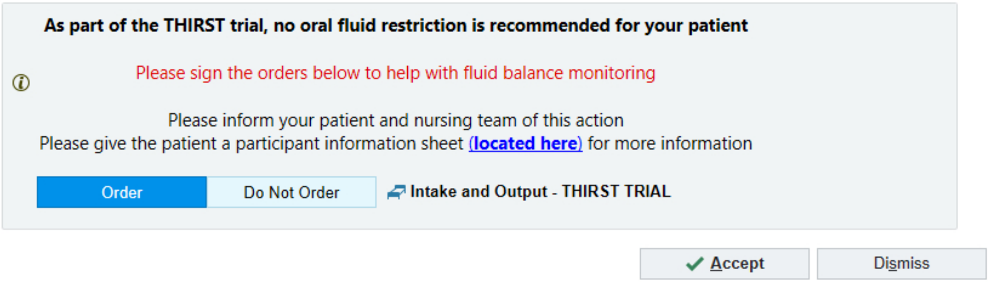


Supplemental Figure 1c. No oral fluid restriction recommendation

550x156mm (38 x 38 DPI)

## Appendix 1 - Trial management group

Dr. Yang Chen (Principal Investigator) – Clinical Research Fellow & Cardiology Registrar

Dr. Tom Lumbers (Chief Investigator) – Associate Professor & Consultant Cardiologist

Dr. Anoop Shah – Associate Professor & Consultant Clinical Pharmacologist

Prof. Matthew Sydes m.sydes@ucl.ac.uk – Professor of Clinical Trials & Methodology

Prof. Folkert W. Asselbergs, f.asselbergs@ucl.ac.uk - Professor of Precision Medicine

For peer review only

## Appendix 2 – Alert Design

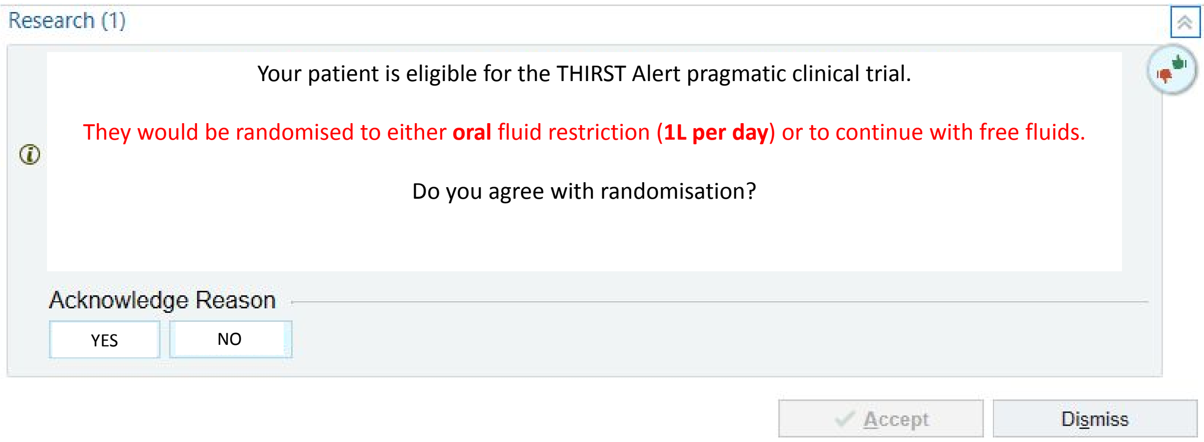


**Supplemental Figure 1a.** THIRST Alert invitation to enrol

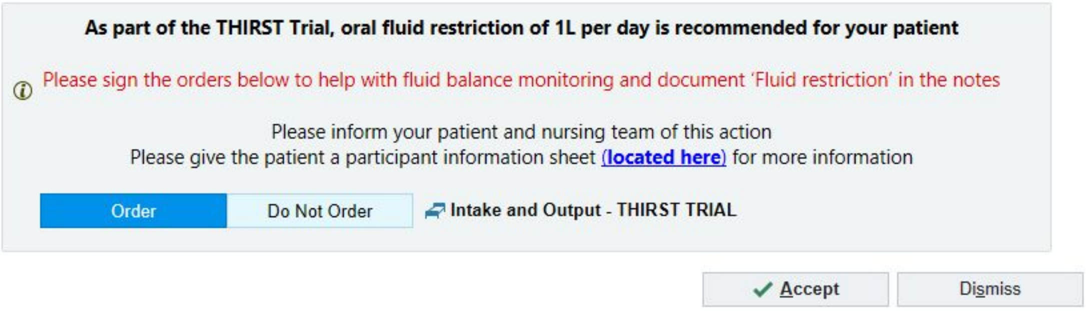


**Supplemental Figure 1b.** Oral fluid restriction recommendation

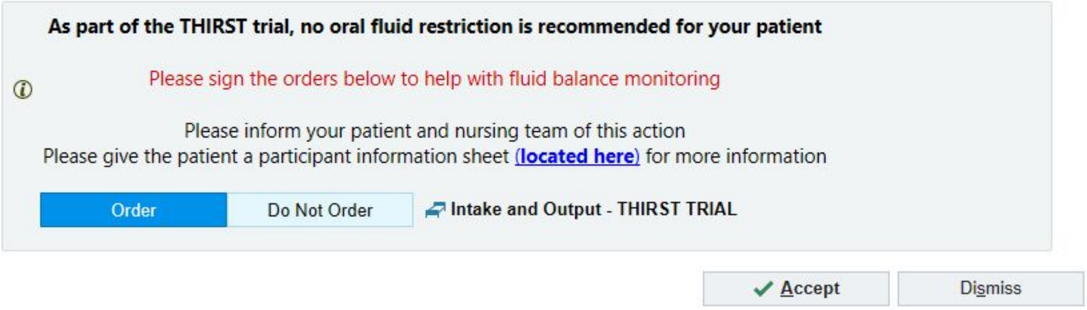


**Supplemental Figure 1c.** No oral fluid restriction recommendation

## Appendix 3 – Fluid restriction at study site

| Fluid restriction status | Total (%) | Primary diagnosis of Heart failure (%) | Heart failure as co-morbidity (%) | No diagnosis of Heart failure (%) |
|---|---|---|---|---|
| **Received fluid restriction at least once** | 826 (56) | 373 (71) | 294 (55) | 159 (38) |
| **Did not receive fluid restriction** | 661 (44) | 152 (29) | 244 (45) | 265 (62) |

**Table S1.** Fluid restriction treatment strategy at the study site prior to trial implementation. Data shown is from unplanned admissions at University College Hospital between April 2019 – October 2022 where patients were prescribed IV furosemide within 48 hours of admission.

For peer review only

## Appendix 4 – Programme Theory for THIRST Alert

For peer review only

| **Event** | **Trigger** | **Action** |
|---|---|---|
| **1.** Patient identification | Based on a *regular or continuous IV* furosemide prescription within 48 hours of unplanned admission | First alert is presented to prescribing clinician after triggering action is performed, most likely in emergency department or acute assessment unit |
| **2.** Alert Presentation | Based on trigger of 'Open Patient Chart' and 'Open Orders Sidebar' | Clinician accepts randomisation – patient enrolled and status on EHR updated to 'research participant' visible to all staff |
| | | Clinician rejects randomisation – patient receives a non-randomised treatment recommendation for fluid restriction or no restriction, in keeping with usual care. |
| | | Clinician defers – alert represented after another trigger action performed by any prescribing clinician within 48 hours |
| **3.** Alert recommendation | Based on clicking 'Yes' to first alert | Fluid restriction alert |
| | | No Fluid restriction alert |
| **4.** Documented evidence of treatment allocation and communication to nursing staff and patient | Alert prompts user to link to document in patient notes. | Documentation of treatment allocation occurs in same manner as routine care. Enrolled patients have an additional workflow within the EHR for nursing staff to document fluid balance |
| | Alert prompts staff to provide additional participant information sheet | A paper PIS is available on all anticipated ward locations and a link supplied to print off a digital copy |
| **5.** Documentation of fluid intake, and other secondary trial outcomes: e.g., fluid balance, weight and patient experience | Prompted by nursing workflow, medical workflow and usual care processes | No additional changes to existing routine care processes |
| **6.** Patient is no longer fluid overloaded | Patient's clinical status has changed and their medical therapy has been de-escalated | No change to usual care processes with regards to continuation or cessation of fluid restriction arm. Trial ends at the end of the admission episode |

For peer review only

**Table S2**. Programme theory for THIRST Alert trial. Steps 1-6 represent a summary of the logic model considered for the trial intervention and its downstream effects

For peer review only

## Appendix 5 – Patient material

For peer review only

For peer review only - http://bmjopen.bmj.com/site/about/guidelines.xhtml

University College London Hospitals NHS
NHS Foundation Trust

# THIRST Alert participant information sheet

**We are inviting you to take part in a research project called the THIRST trial**
We wish to find out if restricting the total daily oral fluid intake in patients treated for fluid overload has any additional benefit to prescribing medications for patients with your condition of fluid overload. The THIRST trial will help to answer this question by providing randomised recommendations on oral fluid intake or no oral fluid intake using the electronic health care record system that the doctors and nurses use to manage your care.

**Why am I being asked to take part in this research?**
Your medical team have initiated medication for the treatment of fluid overload in the body but they are uncertain as to whether or not oral fluid restriction would be of additional benefit. After agreeing to include you in the study, your team will have received a randomised recommendation to either: fluid restriction of no more than 1 litre per day or continuing with your usual amount of oral fluid intake.

Although fluid restriction is a commonly used treatment in your condition, its effectiveness in helping to relieve symptoms is unclear. The mainstay of treatment remains medications to help remove excess water and this is unaffected by whether you restrict fluid or not.

Although you are unlikely to receive any direct benefit from taking part, the results of the study may improve the management of patients with a similar condition in the future.

**Do I have to take part?**
You do not have to take part in the study; you have the right to withdraw at any time. Your decision will not affect the quality of care you receive.

**What will I need to do if I take part?**
You do not need to do anything as a result of taking part. There are no additional tests or appointments. As part of your usual care, you will be asked how you are feeling and how thirsty you are. There are no extra visits to your doctor over and above those needed for your normal care. The study will last only for the time you are in hospital. The normal processes for your discharge and follow up are unaffected by the study.

**How do I opt out?**
If you would not like to be included in this study, please let the doctors and nurses looking after you know or alternatively contact the study team directly.

**What are the disadvantages/risks?**
There are no extra risks involved in taking part in this research. If at any moment you feel like you would like to drink more fluids, you can ask your usual care team for more.

**What will happen to information collected about me during the study?**
Your medical information will be kept strictly confidential by your doctor. The researchers will only be given as much information from your medical records as is needed for this research and that information will be anonymised. They will not be given your name, where you live or anything that could identify you. The results of this research will be made available to all those taking part who would like to receive it.

**Who is organising and funding the research?**


For peer review only - http://bmjopen.bmj.com/site/about/guidelines.xhtml

This study is being carried out by Dr Yang Chen, Dr Anoop Shah, Professor Folkert Asselbergs and Dr Tom Lumbers. It is sponsored by University College London and has support from the Clinical Research Informatics Unit of University College London Hospital (UCLH) and the Biomedical Research Centre at UCLH.

**Further Information**

You can ask your medical team any questions you may have about the study. You may also obtain more detailed information about this research, including how your medical information will be used, your privacy protected, and the compensation arrangements in the unlikely event that anything goes wrong from the following contacts:

**UCLH Patient Advice & Liaison Service (PALS)**

**Address**: PALS
Ground Floor Atrium
University College Hospital
235 Euston Road
London NW1 2BU

**Telephone** (main hospital): 02034473042

**Email:** Uclh.pals@nhs.net

**Study team contact details**:

Dr Yang Chen [Principal investigator: yang.chen@nhs.net]

Dr Tom Lumbers [Chief Investigator: tom.lumbers@nhs.net]

Cardiology Department at UCLH: 020 3447 8066

A description of the study is also available on the following UCLH website, and we plan to publish the study results there too: https://www.uclhospitals.brc.nihr.ac.uk/current-work

Thank you for reading this information.

For peer review only



For peer review only - http://bmjopen.bmj.com/site/about/guidelines.xhtml

University College London Hospitals NHS
NHS Foundation Trust

# Pragmatic research during routine care at UCLH - THIRST Alert trial

## A study of patients with fluid overload who may be suitable for fluid restriction

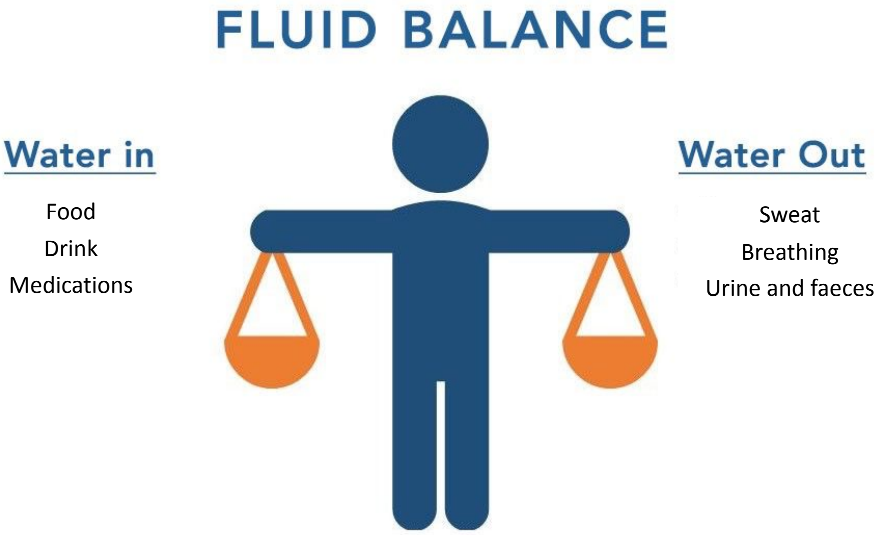


- Your medical team will inform you whether you are suitable for the study and if you will receive either (1) oral fluid restriction of up to 1 litre per day or (2) no restriction of oral fluids
- No other aspect of your care will be affected
- If you do not wish to take part, please let your clinical team know or contact the study team. Please see here for more information:

Study team and contact details:
Yang.chen@nhs.net - Clinical research fellow
Tom.lumbers@nhs.net - Consultant cardiologist
Cardiology Department at UCLH: 020 3447 8066

THIRST Alert

Study Poster Version No. 1.0 ………… Date…03 May 2023…………
IRAS 313551

For peer review only - http://bmjopen.bmj.com/site/about/guidelines.xhtml

STANDARD PROTOCOL ITEMS: RECOMMENDATIONS FOR INTERVENTIONAL TRIALS

SPIRIT 2013 Checklist: Recommended items to address in a clinical trial protocol and related documents*

| Section/item | Item No | Description | Addressed on page number |
|---|---|---|---|
| **Administrative information** | | | |
| Title | 1 | Descriptive title identifying the study design, population, interventions, and, if applicable, trial acronym | _____1________ |
| Trial registration | 2a | Trial identifier and registry name. If not yet registered, name of intended registry | _____3________ |
| | 2b | All items from the World Health Organization Trial Registration Data Set | ____ 3______ |
| Protocol version | 3 | Date and version identifier | _____N/A____ |
| Funding | 4 | Sources and types of financial, material, and other support | _____14________ |
| Roles and responsibilities | 5a | Names, affiliations, and roles of protocol contributors | _____1_______ |
| | 5b | Name and contact information for the trial sponsor | _____14________ |
| | 5c | Role of study sponsor and funders, if any, in study design; collection, management, analysis, and interpretation of data; writing of the report; and the decision to submit the report for publication, including whether they will have ultimate authority over any of these activities | _____14_______ |
| | 5d | Composition, roles, and responsibilities of the coordinating centre, steering committee, endpoint adjudication committee, data management team, and other individuals or groups overseeing the trial, if applicable (see Item 21a for data monitoring committee) | _____21_______ |

For peer review only

**Introduction**

| | | | |
|---|---|---|---|
| Background and rationale | 6a | Description of research question and justification for undertaking the trial, including summary of relevant studies (published and unpublished) examining benefits and harms for each intervention | ____4-6______ |
| | 6b | Explanation for choice of comparators | ____5-6_______ |
| Objectives | 7 | Specific objectives or hypotheses | _____6_______ |
| Trial design | 8 | Description of trial design including type of trial (eg, parallel group, crossover, factorial, single group), allocation ratio, and framework (eg, superiority, equivalence, noninferiority, exploratory) | ____ 7______ |

**Methods: Participants, interventions, and outcomes**

| | | | |
|---|---|---|---|
| Study setting | 9 | Description of study settings (eg, community clinic, academic hospital) and list of countries where data will be collected. Reference to where list of study sites can be obtained | _____7________ |
| Eligibility criteria | 10 | Inclusion and exclusion criteria for participants. If applicable, eligibility criteria for study centres and individuals who will perform the interventions (eg, surgeons, psychotherapists) | __8 and Table 1 |
| Interventions | 11a | Interventions for each group with sufficient detail to allow replication, including how and when they will be administered | __8_and Figure S1 |
| | 11b | Criteria for discontinuing or modifying allocated interventions for a given trial participant (eg, drug dose change in response to harms, participant request, or improving/worsening disease) | ___8-9_______ |
| | 11c | Strategies to improve adherence to intervention protocols, and any procedures for monitoring adherence (eg, drug tablet return, laboratory tests) | ___NA_________ |
| | 11d | Relevant concomitant care and interventions that are permitted or prohibited during the trial | ___7__________ |
| Outcomes | 12 | Primary, secondary, and other outcomes, including the specific measurement variable (eg, systolic blood pressure), analysis metric (eg, change from baseline, final value, time to event), method of aggregation (eg, median, proportion), and time point for each outcome. Explanation of the clinical relevance of chosen efficacy and harm outcomes is strongly recommended | ___9________ |
| Participant timeline | 13 | Time schedule of enrolment, interventions (including any run-ins and washouts), assessments, and visits for participants. A schematic diagram is highly recommended (see Figure) | __Figure 1 and 9_ |



For peer review only - http://bmjopen.bmj.com/site/about/guidelines.xhtml

| | | | |
|---|---|---|---|
| Sample size | 14 | Estimated number of participants needed to achieve study objectives and how it was determined, including clinical and statistical assumptions supporting any sample size calculations | ___10__________ |
| Recruitment | 15 | Strategies for achieving adequate participant enrolment to reach target sample size | ___8_______ |

**Methods: Assignment of interventions (for controlled trials)**

Allocation:

| | | | |
|---|---|---|---|
| Sequence generation | 16a | Method of generating the allocation sequence (eg, computer-generated random numbers), and list of any factors for stratification. To reduce predictability of a random sequence, details of any planned restriction (eg, blocking) should be provided in a separate document that is unavailable to those who enrol participants or assign interventions | ___12________ |
| Allocation concealment mechanism | 16b | Mechanism of implementing the allocation sequence (eg, central telephone; sequentially numbered, opaque, sealed envelopes), describing any steps to conceal the sequence until interventions are assigned | ___9__________ |
| Implementation | 16c | Who will generate the allocation sequence, who will enrol participants, and who will assign participants to interventions | ____9_______ |
| Blinding (masking) | 17a | Who will be blinded after assignment to interventions (eg, trial participants, care providers, outcome assessors, data analysts), and how | ____9_________ |
| | 17b | If blinded, circumstances under which unblinding is permissible, and procedure for revealing a participant's allocated intervention during the trial | ___NA______ |

**Methods: Data collection, management, and analysis**

| | | | |
|---|---|---|---|
| Data collection methods | 18a | Plans for assessment and collection of outcome, baseline, and other trial data, including any related processes to promote data quality (eg, duplicate measurements, training of assessors) and a description of study instruments (eg, questionnaires, laboratory tests) along with their reliability and validity, if known. Reference to where data collection forms can be found, if not in the protocol | ____10_________ |
| | 18b | Plans to promote participant retention and complete follow-up, including list of any outcome data to be collected for participants who discontinue or deviate from intervention protocols | ____10_________ |

| | | | |
|---|---|---|---|
| Data management | 19 | Plans for data entry, coding, security, and storage, including any related processes to promote data quality (eg, double data entry; range checks for data values). Reference to where details of data management procedures can be found, if not in the protocol | ___10-11_______ |
| Statistical methods | 20a | Statistical methods for analysing primary and secondary outcomes. Reference to where other details of the statistical analysis plan can be found, if not in the protocol | ___10___________ |
| | 20b | Methods for any additional analyses (eg, subgroup and adjusted analyses) | ____NA______ |
| | 20c | Definition of analysis population relating to protocol non-adherence (eg, as randomised analysis), and any statistical methods to handle missing data (eg, multiple imputation) | ____NA_______ |
| **Methods: Monitoring** | | | |
| Data monitoring | 21a | Composition of data monitoring committee (DMC); summary of its role and reporting structure; statement of whether it is independent from the sponsor and competing interests; and reference to where further details about its charter can be found, if not in the protocol. Alternatively, an explanation of why a DMC is not needed | ____NA________ |
| | 21b | Description of any interim analyses and stopping guidelines, including who will have access to these interim results and make the final decision to terminate the trial | ____10-11_____ |
| Harms | 22 | Plans for collecting, assessing, reporting, and managing solicited and spontaneously reported adverse events and other unintended effects of trial interventions or trial conduct | ____11_________ |
| Auditing | 23 | Frequency and procedures for auditing trial conduct, if any, and whether the process will be independent from investigators and the sponsor | ____N/A_______ |
| **Ethics and dissemination** | | | |
| Research ethics approval | 24 | Plans for seeking research ethics committee/institutional review board (REC/IRB) approval | ____11_______ |
| Protocol amendments | 25 | Plans for communicating important protocol modifications (eg, changes to eligibility criteria, outcomes, analyses) to relevant parties (eg, investigators, REC/IRBs, trial participants, trial registries, journals, regulators) | ____11_________ |

| | | | |
|---|---|---|---|
| Consent or assent | 26a | Who will obtain informed consent or assent from potential trial participants or authorised surrogates, and how (see Item 32) | ______11_________ |
| | 26b | Additional consent provisions for collection and use of participant data and biological specimens in ancillary studies, if applicable | ______N/A_______ |
| Confidentiality | 27 | How personal information about potential and enrolled participants will be collected, shared, and maintained in order to protect confidentiality before, during, and after the trial | ______10________ |
| Declaration of interests | 28 | Financial and other competing interests for principal investigators for the overall trial and each study site | ______14_________ |
| Access to data | 29 | Statement of who will have access to the final trial dataset, and disclosure of contractual agreements that limit such access for investigators | ______11_________ |
| Ancillary and post-trial care | 30 | Provisions, if any, for ancillary and post-trial care, and for compensation to those who suffer harm from trial participation | ______N/A______ |
| Dissemination policy | 31a | Plans for investigators and sponsor to communicate trial results to participants, healthcare professionals, the public, and other relevant groups (eg, via publication, reporting in results databases, or other data sharing arrangements), including any publication restrictions | ______12_________ |
| | 31b | Authorship eligibility guidelines and any intended use of professional writers | ______14_________ |
| | 31c | Plans, if any, for granting public access to the full protocol, participant-level dataset, and statistical code | ______N/A________ |
| **Appendices** | | | |
| Informed consent materials | 32 | Model consent form and other related documentation given to participants and authorised surrogates | _____Appendices |
| Biological specimens | 33 | Plans for collection, laboratory evaluation, and storage of biological specimens for genetic or molecular analysis in the current trial and for future use in ancillary studies, if applicable | ___N/A_________ |

*It is strongly recommended that this checklist be read in conjunction with the SPIRIT 2013 Explanation & Elaboration for important clarification on the items. Amendments to the protocol should be tracked and dated. The SPIRIT checklist is copyrighted by the SPIRIT Group under the Creative Commons "Attribution-NonCommercial-NoDerivs 3.0 Unported" license.



For peer review only - http://bmjopen.bmj.com/site/about/guidelines.xhtml

BMJ Open

# Rationale and design of the THIRST Alert feasibility study: a pragmatic, parallel group randomised controlled trial of an interruptive alert for oral fluid restriction in patients admitted to hospital and treated with intravenous furosemide

| | |
|---|---|
| Journal: | *BMJ Open* |
| Manuscript ID | bmjopen-2023-080410.R1 |
| Article Type: | Protocol |
| Date Submitted by the Author: | 05-Dec-2023 |
| Complete List of Authors: | Chen, Yang ; University College London, Institute of Health Informatics; University College London Hospitals NHS Foundation Trust, Clinical and Research Informatics Unit, NIHR UCLH Biomedical Research Centre<br>Shah, Anoop; University College London, Institute of Health Informatics; University College London Hospitals NHS Foundation Trust, Clinical and Research Informatics Unit, NIHR UCLH Biomedical Research Centre<br>Jani, Yogini; University College London Hospitals NHS Foundation Trust, Centre for Medicines Optimisation Research & Education - CMORE<br>Higgins, Daniel; University College London Hospitals NHS Foundation Trust, Clinical and Research Informatics Unit, NIHR UCLH Biomedical Research Centre<br>Saleem, Nausheen; University College London Hospitals NHS Foundation Trust, Clinical and Research Informatics Unit, NIHR UCLH Biomedical Research Centre<br>Chafer, Kris; University College London Hospitals NHS Foundation Trust, Clinical and Research Informatics Unit, NIHR UCLH Biomedical Research Centre<br>Sydes, Matthew; Medical Research Council Clinical Trials Unit at University College London, MRC Clinical Trials Unit, Institute of Clinical Trials and Methodology; Health Data Research UK,<br>Asselbergs, Folkert; University College London, Institute of Health Informatics; University of Amsterdam, Department of Cardiology, Amsterdam Cardiovascular Sciences, Amsterdam University Medical Centre<br>Lumbers, R Thomas; University College London, Institute of Health Informatics |
| <b>Primary Subject Heading</b>: | Cardiovascular medicine |
| Secondary Subject Heading: | Health informatics, Research methods |
| Keywords: | Heart failure < CARDIOLOGY, Randomized Controlled Trial, Feasibility Studies, Electronic Health Records |
| | |

# Rationale and design of the THIRST Alert feasibility study: a pragmatic, parallel group randomised controlled trial of an interruptive alert for oral fluid restriction in patients admitted to hospital and treated with intravenous furosemide

Yang Chen[1,2]
Anoop D. Shah[1,2,]
Yogini Jani[3]
Daniel Higgins[2]
Nausheen Saleem[2]
Kris Chafer[2]
Matthew R Sydes[5,]
Folkert W Asselbergs[1,2,4]
R Thomas Lumbers[1]

1. Institute of Health Informatics, Faculty of Population Health Sciences, University College London, UK
2. Clinical and Research Informatics Unit, The National Institute for Health and Care Research University College London Hospitals Biomedical Research Centre, UCL, London, UK
3. Centre for Medicines Optimisation Research and Education, University College London Hospitals NHS Foundation Trust, London, UK
4. Department of Cardiology, Amsterdam Cardiovascular Sciences, Amsterdam University Medical Centre, University of Amsterdam, Amsterdam, Netherlands
5. MRC Clinical Trials Unit at UCL, Institute of Clinical Trials and Methodology, University College London, London, UK

**Corresponding author:** R. Thomas Lumbers

**E-mail:** t.lumbers@ucl.ac.uk

**Word count (**exc table/ figures/ legends/ acknowledgements**): 2222**

For peer review only

# Abstract

**Introduction**

Acute heart failure (HF) is a major cause of unplanned hospitalisation characterised by excess body water. A restriction in oral fluid intake is commonly imposed on patients as an adjunct to pharmacological therapy with loop diuretics, but there is a lack of evidence from traditional randomised controlled trials (RCTs) to support the safety and effectiveness of this intervention in the acute setting.

This study aims to explore the feasibility of using computer alerts within the electronic health record (EHR) system to invite clinical care teams to enrol patients into a pragmatic RCT at the time of clinical decision-making. It will additionally assess the effectiveness of using an alert to help address the clinical research question of whether oral fluid restriction is a safe and effective adjunct to pharmacological therapy for patients admitted with fluid overload.

**Methods and analysis**

THIRST Alert is a single centre, parallel group, open-label pragmatic RCT embedded in the EHR system that will be conducted as a feasibility study. The clinical care team will be invited to enrol suitable patients in the study using a point-of-care alert with a target sample size of 50 patients. Enrolled patients will then be randomised to either restricted or unrestricted oral fluid intake. Two primary outcomes will be explored: (1) the proportion of eligible patients enrolled in the study and (2) the mean difference in oral fluid intake between randomised groups. A series of secondary outcomes are specified to evaluate the


For peer review only

effectiveness of the alert, adherence to the randomised treatment allocation, and the quality of data generated from routine care, relevant to the outcomes of interest.

**Ethics and dissemination**

This study was approved by Riverside Research Ethics Committee (Ref: 22/LO/0889) and will be published on completion. Trial registration number NCT05869656.

**Keywords**

*Embedded trials; point-of-care randomisation; fluid overload; fluid restriction; heart failure*

**Strengths and Limitations of this study**

- THIRST Alert is a pragmatic randomised controlled trial where all elements of trial conduct are embedded within the routine care clinical pathway
- The intervention and programme theory have been co-designed with a multidisciplinary team including physicians, nurses, clinical informatics officers and patient representatives
- A proportionate consent model has been approved for use to help with the practicality of trial conduct
- Uncertainty remains regarding data quality in relation to the ascertainment of study outcomes from routine hospital care records.

For peer review only

# Introduction

Acute heart failure (HF) is an important cause of unplanned hospitalisation and is characterised by excess body water, also known as congestion. (1, 2) Despite being a common clinical problem, there is a lack of high-quality evidence to guide drug treatment and non-pharmacological measures. (3) Restriction of oral fluid intake is commonly used as an adjunct to pharmacological therapy but its effectiveness is uncertain and may exacerbate symptoms of thirst, as suggested by the limited randomised controlled trials which have been conducted to date (4–6). These previous trials achieved only small differences in oral fluid intake, which may reflect the Hawthorne effect whereby participation in a clinical trial influences fluid intake in both groups. (7)

Patients and clinicians identify the need for better treatment of fluid overload as a key priority, and the use of oral fluid restriction is also highlighted a gap in evidence in clinical practice guidelines (2, 8) (9). To address this evidence gap and some of the challenges encountered in previous studies, we designed a pragmatic RCT embedded in the electronic health record (EHR) system to evaluate the safety and effectiveness of oral fluid restriction in acute HF.

## Patient population

Fluid overload may be caused by liver or kidney dysfunction (10, 11), as well as by heart failure, with similar evidence gaps for congestion management in such circumstances. (12) (13) (14). However, a reliance on evaluating interventions for the immediate management of fluid overload only where the underlying cause is established may impact the

For peer review only

generalisability of any findings. Given that standardised clinical scores for congestion are not well validated or adopted in practice(15, 16), we used the prescription of two consecutive doses of intravenous furosemide within the first 48 hours of admission as evidence of a physician-assessed ‘treatable trait’ of fluid congestion, to define the study population. (17) At our proposed study site, more than one consecutive dose of intravenous furosemide was administered during the first 48 hours of admission in 1537 unplanned admissions between April 2019 and October 2022. In 56% of these admissions with available supporting data, oral fluid restriction was recommended (**Appendix 1**). The use of prescribing intention to identify the study population enabled digital enrolment into the study based on a discrete triggering event. To our knowledge, there has been no previous RCT conducted in all patients treated for congestion with intravenous furosemide in the acute care setting.

## Pragmatic research

There are many evidence gaps in routine clinical practice that have not been addressed by conventional randomised controlled trials which may be prohibitively expensive. Pragmatic clinical trials (PCT) offer a potential solution, particularly relevant to the evaluation of low-risk interventions where clinical equipoise may exist. By integrating a randomisation procedure into routine care pathways, automating case identification through the EHR, engaging the clinical care team for patient recruitment, and using routinely collected data for trial outcomes, there is the potential to perform large-scale trials at low-cost and low burden to patients and their caregivers. Many questions remain however, about how best to deliver PCTs embedded in EHR systems, including the design of physician-facing alerts and different models of consent. For our study, we employ an interruptive alert to provide the routine care team with an invitation to enrol eligible patients, and use a proportionate

For peer review only - http://bmjopen.bmj.com/site/about/guidelines.xhtml

process for consent that can be delivered by the same team.

PCTs have the potential to enable evidence generation during routine care, helping to conduct more efficient and representative trials.(18) Through examining the comparative effectiveness of existing interventions that have demonstrable variation in practice, many evidence gaps may be addressed. The use of oral fluid restriction in patients who are treated pharmacologically for fluid overload in the setting of acute unplanned care represents one such gap.

The THIRST Alert trial therefore aims to examine (1) whether interruptive alerts are an effective means to engage the usual care team in enrolling patients in an interventional pragmatic RCT; and (2), for enrolled patients, whether the randomised assignment to either oral fluid restriction to 1-litre per day or no oral fluid restriction leads to a difference in documented oral fluid intake. The evidence generated will be used to inform the design of subsequent multi-centre outcomes driven pragmatic clinical trials embedded in EHR systems.

For peer review only

For peer review only

# Methods and analysis

## Study design

THIRST Alert is a single centre, parallel group, open-label pragmatic RCT embedded in the EHR system that will be conducted as a feasibility study.(19) The study will be delivered entirely at University College Hospital, UCLH, a digitally mature hospital. (20) Enrolment started in May 2023 and the estimated trial completion date is December 2023. Given the low risk nature of the intervention, a verbal, opt-out consent model is used. The study protocol has been prepared with reference to the SPIRIT statement.(21) The trial is registered at ClinicalTrials.gov [NCT05869656]

## Study procedures

An overview of the study procedures is given in **Figure 1**. The trial was designed to be integrated into the electronic health record system EPIC (Epic, Epic Systems, Verona, WI). The screening and identification of eligible patients, display of interruptive alerts to physicians, randomisation and outcome ascertainment are all provisioned within EPIC. (22) Education events, internal communication and email correspondence were delivered to members of the routine care team to raise awareness of the study but no formal co-interventions were undertaken. The conduct and analysis of the trial is overseen by a trial management group who conceived a programme theory for the alert with relevant multidisciplinary input (**Appendix 2-3**). The enrolment invitation and randomised treatment recommendations are classified as complex interventions according to the Medical Research Council framework. (23)

For peer review only

The trial is designed to maximise the generalisability and applicability of the evidence generated and is highly pragmatic according to the PRECIS-2 framework (**Figure 2**). (24)

## Study participants

Adults of 18 years or older who were administered more than one dose of intravenous (IV) furosemide within the first 48 hours of an unplanned admission were eligible for the study. Patients admitted to a surgical, maternity or critical care ward were excluded **(Table 1)**.

## The interruptive alert inviting usual care team to enrol eligible patients

Any member of the clinical team may receive an interruptive alert (**Appendix 4, supplemental figure 1a**) during their interaction with EPIC for an eligible patient, informing them of the study and inviting them to enrol the patient. The clinician can click the appropriate button on the alert to enrol, decline to enrol, or defer the decision regarding enrolment. The alert is non-coercive, and the decision about patient enrolment is at the discretion of the treating clinician. We anticipate that physicians will enrol patients in cases where they judge that there is clinical equipoise as to whether oral fluid restriction is likely to benefit the patient. The alert was designed and tested with EPIC application specialists in an iterative manner, integrating feedback from end-users on the wording of the design.

## The randomised treatment intervention

After enrolment of patients in the study, the routine care team receives a recommendation regarding oral fluid intake and assignment is determined by point of care randomisation. The randomised treatment allocation is only presented to the clinical team after they click 'yes' to the THIRST alert, rather than displayed immediately, to minimise the possibility of

For peer review only

differential recruitment to the treatment arms of the trial. For patients allocated to the intervention group, the routine care team receive a recommendation for a target oral fluid restriction of 1 litre per day; for patients allocated to the control group, the care team receive a recommendation for unrestricted oral fluid intake (**Appendix 4, supplemental figure 1b and 1c**). The oral fluid restriction target was chosen based on previous RCTs and acceptability in clinical practice (4–6). The randomised treatment recommendation for both groups additionally advises the clinician to: (1) complete an EPIC order for fluid balance monitoring; (2) document the treatment allocation in the clinical notes (3) inform the patient and the nursing team and (4) provide the patient with a participant information sheet.

## Randomisation and blinding

Subjects will be randomised 1:1 using an internal random number rule implemented using a standard EPIC randomisation tool used in other PCTs.(25) No block randomisation or additional covariate balancing will be undertaken. The study is open label with patients and their care teams being made aware of the treatment allocation.

## Study Outcomes

### Primary outcomes

1. Number of eligible patients randomised
2. Difference in oral fluid intake between intervention and control arms

### Secondary outcomes

1. Adherence to randomised treatment recommendation
2. Proportion of alerts resulting in clinical orders for nursing care
3. Oral fluid intake
4. Net fluid balance
5. Weight change after randomisation
6. Length of stay

For peer review only - http://bmjopen.bmj.com/site/about/guidelines.xhtml

7. Frequency of blood test measurements of renal function
8. Prescription of diuretic medications
9. Daily change in Creatinine
10. Patient Reported Outcome Measures

These outcomes were chosen based on clinical relevance and the ability to detect the possibility of attributable harm during an inpatient admission.

## Sample size and power calculation

No formal sample size calculation was performed given the primary outcomes pertain to the feasibility of using an EHR alert aimed at the routine care team to (i) enrol eligible patients into an RCT of oral fluid restriction and (ii) cause and sustain the allocated clinical treatment effect, as measured by documented oral fluid intake. This is in keeping with the role of feasibility studies used by other groups. (26) Our target sample size was 50 patients, based on the predicted number of eligible patients at the study site during the recruitment period.

## Data collection, curation and storage

Baseline characteristics and study outcomes will be extracted from the routine care record. Information on the frequency of the interruptive alert and staff responses to alerts will be recorded in EPIC. We will also extract primary and secondary ICD-10 diagnosis codes assigned to the clinical episode by the clinical coding team. Study data will be transferred to a secure research environment within UCLH NHS Foundation Trust as pseudo-anonymised electronic Case Report Forms (eCRFs). No data will leave the UCLH NHS system. Patients who opt out or withdraw from the trial will have their decision documented on the eCRF and their data will not be included in the main analysis.

For peer review only

## Statistical analysis plan

Baseline characteristics of study participants will be summarised using percentages or means and standard deviations as appropriate and compared using t tests for continuous variables (expressed as mean ± standard deviation) and the $\chi^2$ tests or the Fisher's exact tests for categorical variables (expressed as count and percentage). Secondary endpoints for intervention and control groups will be compared using $\chi^2$ test for binary outcomes, and T-test for continuous outcomes. For subgroup analyses, linear or logistic regression will be used to determine the effects of treatment allocation according to heart failure status. No adjustment or imputation will be used for missing data. For this feasibility study, we considered a sample of 20 participants enrolled as a minimum number acceptable for feasibility and a daily mean difference of 250ml of fluid intake between treatment arms as clinically relevant. Progression to a multi-centre clinical outcomes trial will be contingent on a series of stop/ go criteria based on achieving the primary outcome targets and on secondary outcomes relating to data quality.

## Monitoring and Evaluation

The trial will report serious adverse events (SAEs) that are attributable to the study through the EHR, in accordance with the safety reporting processes and sponsor policy. (27) The Trial Management Group will review recruitment rates, SAEs and any substantial amendments to the protocol.

## Patient and public involvement (PPI)

Patients were integrally involved in the design of the study as previously reported. (28) In brief this involved a patient participation event where options for consent and outcome

For peer review only

measures were explored which subsequently informed the study design. In accordance with the National Institute for Health Research guidance on co-production(29), one participant (KC) served as a patient advisor to the trial and reviewed all trial materials.

# Ethics and Dissemination

This study protocol was approved by the London Riverside Research Ethics Committee (Ref: 22/LO/0889) and sponsored by University College London (Ref: 151938). A verbal opt-out model of consent was adopted based on the negligible risk associated with the intervention. Patients are informed of their study participation and of their treatment allocation and can opt-out of the study or decline to follow the recommendation for oral fluid restriction at any time. A copy of the sample participant information sheet (PIS) is provided to patients (**Appendix 5**). The data management for the trial conforms to NHS Information standards DCB0129 and DCB0160 and was approved by University College London Hospitals NHS Foundation Trust (UCLH) Digital Services Safety board. (30) The alert is not classified as a separate piece of software but is incorporated within the Epic electronic health record, which is registered as a Class I medical device by the UK Medicines and Healthcare products Regulatory Agency. (31, 32) The study findings will be disseminated through publication in open-access peer-reviewed journals and through the provision of reports for patients and clinicians involved in the study. As a feasibility study, the findings shared will include trial evaluation, and whether alerts which ‘nudge’ clinicians to recruit patients into pragmatic, low-risk comparative effectiveness trials (33), are a scalable way to conduct efficient

For peer review only

RCTs.(18) The THIRST Alert feasibility study will share insights that inform the design and execution of future integrated approaches to evidence generation from routine care.

## Author contributions

YC, AS, MS, FA, and TL conceived the study idea. YC and TL wrote the first draft of the protocol. YJ, DH and NS helped to design, test and review the study alert. KC reviewed and edited the patient-facing study materials. All authors reviewed the manuscript and contributed to revisions.

## Acknowledgements

We are grateful to clinical colleagues, Inaki Elizondo, Pamela Stephenson, Sian Roberts-Walsh, Alexander Tranter and Ali Hosin for assistance with the study design. We thank Steve Harris and Pier Lambiase for comments on the study protocol and to members of the digital safety committee with the safe implementation of this study.

## Funding statement

This work was supported by the National Institute for Health and Care Research (NIHR) UCLH Biomedical Research Centre (BRC), award number NIHR-INF-1873. The study funder did not play any role in the design of the study.

## Conflict of interests

R.T.L has received funding from Pfizer for unrelated research, under the Innovative Target Exploration Network programme.

For peer review only

For peer review only

# References

1. Savarese G, Becher PM, Lund LH, Seferovic P, Rosano GMC, Coats A. Global burden of heart failure: A comprehensive and updated review of epidemiology. Cardiovasc Res. 2022; cvac013.

2. McDonagh TA, Metra M, Adamo M, Gardner RS, Baumbach A, Böhm M, et al. 2021 ESC Guidelines for the diagnosis and treatment of acute and chronic heart failure. European Heart Journal. 2021; 42(36):3599–726.

3. Koratala A, Ronco C, Kazory A. Diagnosis of Fluid Overload: From Conventional to Contemporary Concepts. Cardiorenal Med. 2022; 12(4):141–54.

4. Aliti GB, Rabelo ER, Clausell N, Rohde LE, Biolo A, Beck-da-Silva L. Aggressive fluid and sodium restriction in acute decompensated heart failure: a randomized clinical trial. JAMA Intern Med. 2013; 173(12):1058–64.

5. Machado d'Almeida KS, Rabelo-Silva ER, Souza GC, Trojahn MM, Santin Barilli SL, Aliti G, et al. Aggressive fluid and sodium restriction in decompensated heart failure with preserved ejection fraction: Results from a randomized clinical trial. Nutrition. 2018; 54:111–7.

6. Travers B, O'Loughlin C, Murphy NF, Ryder M, Conlon C, Ledwidge M, et al. Fluid restriction in the management of decompensated heart failure: no impact on time to clinical stability. J Card Fail. 2007; 13(2):128–32.

7. Sedgwick P, Greenwood N. Understanding the Hawthorne effect. BMJ. 2015; h4672.

8. Heidenreich PA, Bozkurt B, Aguilar D, Allen LA, Byun JJ, Colvin MM, et al. 2022 ACC/AHA/HFSA Guideline for the Management of Heart Failure. Journal of Cardiac Failure. 2022; 28(5):e1–167.

9. Taylor CJ, Huntley AL, Burden J, Gadoud A, Gronlund T, Jones NR, et al. Research priorities in advanced heart failure: James Lind alliance priority setting partnership. Open Heart. 2020; 7(1):e001258.

10. Damman K, Voors AA, Hillege HL, Navis G, Lechat P, Van Veldhuisen DJ, et al. Congestion in chronic systolic heart failure is related to renal dysfunction and increased mortality. European Journal of Heart Failure. 2010; 12(9):974–82.

11. Naschitz JE, Slobodin G, Lewis RJ, Zuckerman E, Yeshurun D. Heart diseases affecting the liver and liver diseases affecting the heart. American Heart Journal. 2000; 140(1):111–20.

12. Aithal GP, Palaniyappan N, China L, Härmälä S, Macken L, Ryan JM, et al. Guidelines on the management of ascites in cirrhosis. Gut. 2021; 70(1):9–29.

13. NICE guideline NG107. RRT and conservative management Evidence review for dietary management and fluid restriction [Internet]. [date unknown]. Available from:

For peer review only

https://www.nice.org.uk/guidance/ng107/evidence/i-dietary-management-and-fluid-restriction-pdf-235524385620.

14. Nice guideline NG203. Chronic kidney disease: assessment and management [Internet]. [date unknown]. Available from: https://www.nice.org.uk/guidance/ng203/resources/chronic-kidney-disease-assessment-and-management-pdf-66143713055173.

15. Boorsma EM, Maaten JM ter, Damman K, Dinh W, Gustafsson F, Goldsmith S, et al. Congestion in heart failure: a contemporary look at physiology, diagnosis and treatment. Nat Rev Cardiol. 2020; 17(10):641–55.

16. Gheorghiade M, Follath F, Ponikowski P, Barsuk JH, Blair JEA, Cleland JG, et al. Assessing and grading congestion in acute heart failure: a scientific statement from the acute heart failure committee of the heart failure association of the European Society of Cardiology and endorsed by the European Society of Intensive Care Medicine. Eur J Heart Fail. 2010; 12(5):423–33.

17. Agusti A, Bel E, Thomas M, Vogelmeier C, Brusselle G, Holgate S, et al. Treatable traits: toward precision medicine of chronic airway diseases. Eur Respir J. 2016; 47(2):410–9.

18. Usman MS, Van Spall HGC, Greene SJ, Pandey A, McGuire DK, Ali ZA, et al. The need for increased pragmatism in cardiovascular clinical trials. Nat Rev Cardiol [Internet]. 2022 [cited 2022 Sep 1]. Available from: https://www.nature.com/articles/s41569-022-00705-w.

19. Eldridge SM, Lancaster GA, Campbell MJ, Thabane L, Hopewell S, Coleman CL, et al. Defining Feasibility and Pilot Studies in Preparation for Randomised Controlled Trials: Development of a Conceptual Framework. PLoS ONE. 2016; 11(3):e0150205.

20. NHS England. Acute global digital exemplars. (online) [Internet]. 2022 [cited 2022 May 17]. Available from: https://www.england.nhs.uk/digitaltechnology/connecteddigitalsystems/exemplars/acute-global-digital-exemplars/.

21. Chan A-W, Tetzlaff JM, Altman DG, Laupacis A, Gøtzsche PC, Krleža-Jerić K, et al. SPIRIT 2013 statement: defining standard protocol items for clinical trials. Ann Intern Med. 2013; 158(3):200–7.

22. Latha M, Bellary S, Krishnankutty B. Basics of case report form designing in clinical research. Perspect Clin Res. 2014; 5(4):159.

23. Skivington K, Matthews L, Simpson SA, Craig P, Baird J, Blazeby JM, et al. A new framework for developing and evaluating complex interventions: update of Medical Research Council guidance. BMJ. 2021; n2061.

24. Loudon K, Treweek S, Sullivan F, Donnan P, Thorpe KE, Zwarenstein M. The PRECIS-2 tool: designing trials that are fit for purpose. BMJ. 2015; 350:h2147.

For peer review only - http://bmjopen.bmj.com/site/about/guidelines.xhtml

25. Wilson FP, Martin M, Yamamoto Y, Partridge C, Moreira E, Arora T, et al. Electronic health record alerts for acute kidney injury: multicenter, randomized clinical trial. BMJ. England; 2021; 372(8900488, bmj, 101090866):m4786.

26. Blatch-Jones AJ, Pek W, Kirkpatrick E, Ashton-Key M. Role of feasibility and pilot studies in randomised controlled trials: a cross-sectional study. BMJ Open. 2018; 8(9):e022233.

27. NHS Health Research Authority. Safety and progress reports (other research) procedural table. (Online). [Internet]. [cited 2023 May 10]. Available from: https://www.hra.nhs.uk/approvals-amendments/managing-your-approval/safety-reporting/safety-and-progress-reports-other-research-procedural-table/.

28. Chen Y, Hosin AA, George MJ, Asselbergs FW, Shah AD. Digital technology and patient and public involvement (PPI) in routine care and clinical research—A pilot study. PLoS ONE. 2023; 18(2):e0278260.

39. Staniszewska S, Denegri S, Matthews R, Minogue V. Reviewing progress in public involvement in NIHR research: developing and implementing a new vision for the future. BMJ Open. 2018; 8(7):e017124.

30. NHS Digital. DCB0160: Clinical Risk Management: its Application in the Deployment and Use of Health IT Systems [Internet]. 2023 [cited 2023 Feb 3]. Available from: https://digital.nhs.uk/data-and-information/information-standards/information-standards-and-data-collections-including-extractions/publications-and-notifications/standards-and-collections/dcb0160-clinical-risk-management-its-application-in-the-deployment-and-use-of-health-it-systems.

31. Medicines and Healthcare products Regulatory Agency. Public Access Registration Database [Internet]. 2023 [cited 2023 Sep 27]. Available from: (https://pard.mhra.gov.uk/manufacturer-details/33914.

32. Medicines and Healthcare products Regulatory Agency. Guidance:Medical devicee stand-alone including apps (including IVDMDs) [Internet]. 2023 [cited 2023 Feb 3]. Available from: https://assets.publishing.service.gov.uk/government/uploads/system/uploads/attachment_data/file/1105233/Medical_device_stand-alone_software_including_apps.pdf.

33. Chen Y, Harris S, Rogers Y, Ahmad T, Asselbergs FW. Nudging within learning health systems: next generation decision support to improve cardiovascular care. Eur Heart J. 2022; 43(13):1296–306.

For peer review only

## Table and Figure Legends

| Study participants | Eligibility Criteria |
|---|---|
| Patients | **Inclusion**<br>- Aged 18 years and over<br>- Prescribed IV furosemide during the first 48h of their admission as a regular prescription rather than a one-off dose.<br>- Assessed as being suitable for inclusion in the trial by the responsible clinical team i.e., fluid restriction deemed to be in equipoise.<br>**Exclusion**<br>- Surgical or obstetric wards<br>- Critical care wards |
| Prescribing clinicians: Physicians (Consultants and Junior Doctors) | Any clinician with prescribing rights and who prescribes more than one dose of IV furosemide within 48 hours of admission, during the trial recruitment period. |
| Nursing staff | A separate order is triggered for enrolled patients and only presented to nursing staff who access their patient record to help facilitate fluid balance documentation |

**Table 1.** Eligibility criteria for THIRST Alert study participants

**Figure 1.** THIRST Alert trial patient flow

**Figure 2.** PRECIS-2 score for THIRST Alert trial. Each of the nine domains are scored on a 5-point Likert continuum (from 1=very explanatory “ideal conditions” to 5=very pragmatic “usual care conditions”)

For peer review only

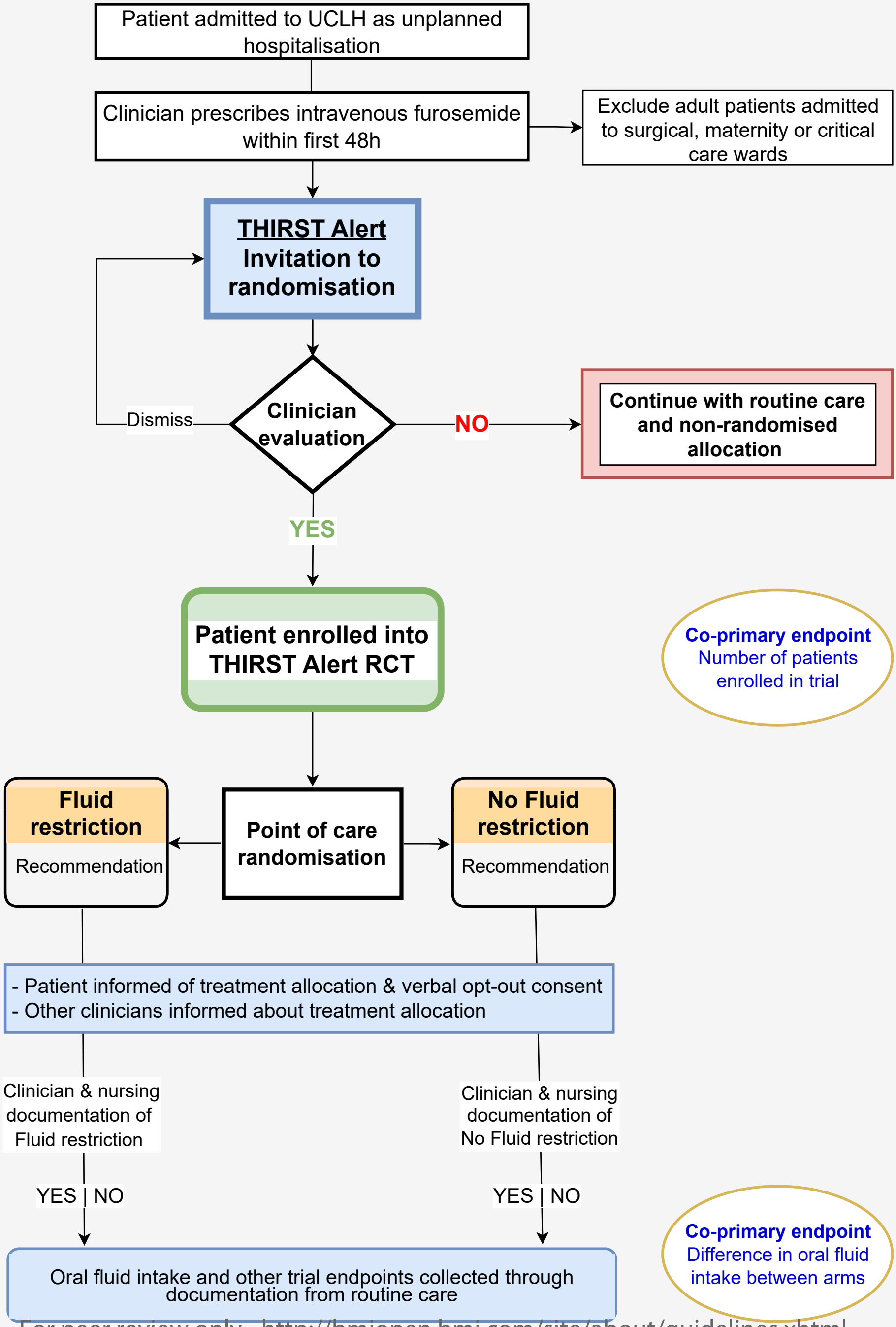
Patient admitted to UCLH as unplanned hospitalisation
Clinician prescribes intravenous furosemide within first 48h
Exclude adult patients admitted to surgical, maternity or critical care wards
THIRST Alert Invitation to randomisation
Dismiss
Clinician evaluation
NO
Continue with routine care and non-randomised allocation
YES
Patient enrolled into THIRST Alert RCT
Co-primary endpoint Number of patients enrolled in trial
Fluid restriction
Recommendation
Point of care randomisation
No Fluid restriction
Recommendation
- Patient informed of treatment allocation & verbal opt-out consent
- Other clinicians informed about treatment allocation
Clinician & nursing documentation of Fluid restriction
Clinician & nursing documentation of No Fluid restriction
YES | NO
YES | NO
Oral fluid intake and other trial endpoints collected through documentation from routine care
Co-primary endpoint Difference in oral fluid intake between arms

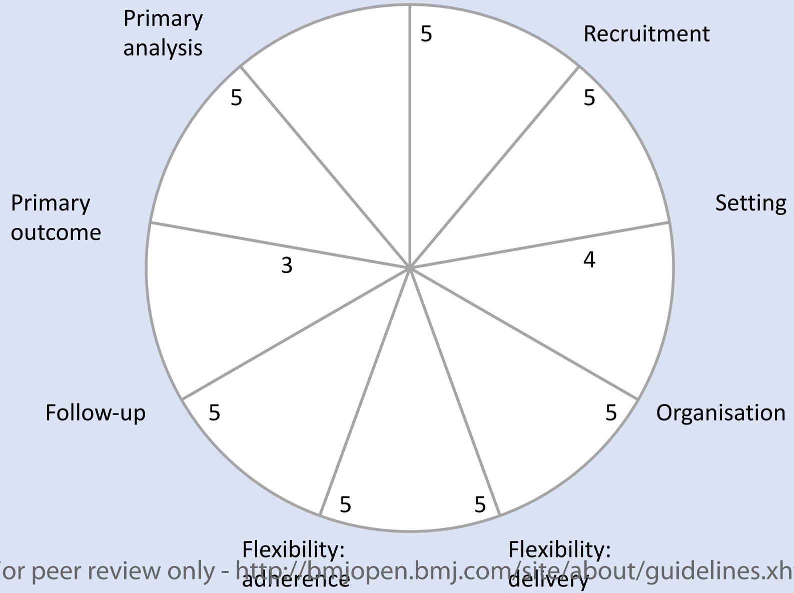


For peer review only - http://bmjopen.bmj.com/site/about/guidelines.xhtml

## Appendix 1 – Fluid restriction at study site

| Fluid restriction status | Total (%) | Primary diagnosis of Heart failure (%) | Heart failure as co-morbidity (%) | No diagnosis of Heart failure (%) |
|---|---|---|---|---|
| **Received fluid restriction at least once** | 826 (56) | 373 (71) | 294 (55) | 159 (38) |
| **Did not receive fluid restriction** | 661 (44) | 152 (29) | 244 (45) | 265 (62) |

**Table S1.** Fluid restriction treatment strategy at the study site prior to trial implementation. Data shown is from unplanned admissions at University College Hospital between April 2019 – October 2022 where patients were prescribed IV furosemide within 48 hours of admission. The total number of patients was 1537 of data was available on heart failure and fluid restriction status in 1487.

## Appendix 2 - Trial management group

Dr. Yang Chen (Principal Investigator) – Clinical Research Fellow & Cardiology Registrar

Dr. Tom Lumbers (Chief Investigator) – Associate Professor & Consultant Cardiologist

Dr. Anoop Shah – Associate Professor & Consultant Clinical Pharmacologist

Prof. Matthew Sydes m.sydes@ucl.ac.uk – Professor of Clinical Trials & Methodology

Prof. Folkert W. Asselbergs, f.asselbergs@ucl.ac.uk - Professor of Precision Medicine

For peer review only

## Appendix 3 – Programme Theory for THIRST Alert

For peer review only

| **Event** | **Trigger** | **Action** |
|---|---|---|
| **1.** Patient identification | Based on a *regular or continuous IV* furosemide prescription within 48 hours of unplanned admission | First alert is presented to prescribing clinician after triggering action is performed, most likely in emergency department or acute assessment unit |
| **2.** Alert Presentation | Based on trigger of 'Open Patient Chart' and 'Open Orders Sidebar' | Clinician accepts randomisation – patient enrolled and status on EHR updated to 'research participant' visible to all staff |
| | | Clinician rejects randomisation – patient receives a non-randomised treatment recommendation for fluid restriction or no restriction, in keeping with usual care. |
| | | Clinician defers – alert represented after another trigger action performed by any prescribing clinician within 48 hours |
| **3.** Alert recommendation | Based on clicking 'Yes' to first alert | Fluid restriction alert |
| | | No Fluid restriction alert |
| **4.** Documented evidence of treatment allocation and communication to nursing staff and patient | Alert prompts user to link to document in patient notes. | Documentation of treatment allocation occurs in same manner as routine care. Enrolled patients have an additional workflow within the EHR for nursing staff to document fluid balance |
| | Alert prompts staff to provide additional participant information sheet | A paper PIS is available on all anticipated ward locations and a link supplied to print off a digital copy |
| **5.** Documentation of fluid intake, and other secondary trial outcomes: e.g., fluid balance, weight and patient experience | Prompted by nursing workflow, medical workflow and usual care processes | No additional changes to existing routine care processes |
| **6.** Patient is no longer fluid overloaded | Patient's clinical status has changed and their medical therapy has been de-escalated | No change to usual care processes with regards to continuation or cessation of fluid restriction arm. Trial ends at the end of the admission episode |

**Table S2**. Programme theory for THIRST Alert trial. Steps 1-6 represent a summary of the logic model considered for the trial intervention and its downstream effects

For peer review only

## Appendix 4 – Alert Design

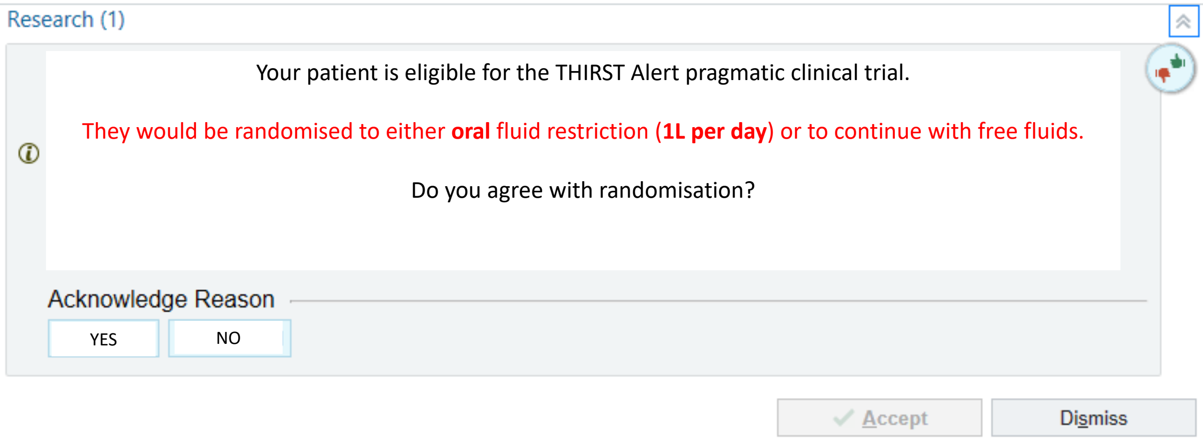


**Supplemental Figure 1a.** THIRST Alert invitation to enrol

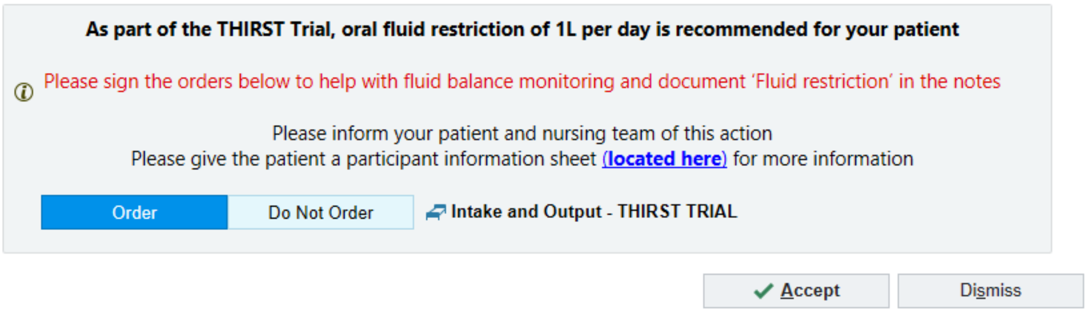


**Supplemental Figure 1b.** Oral fluid restriction recommendation

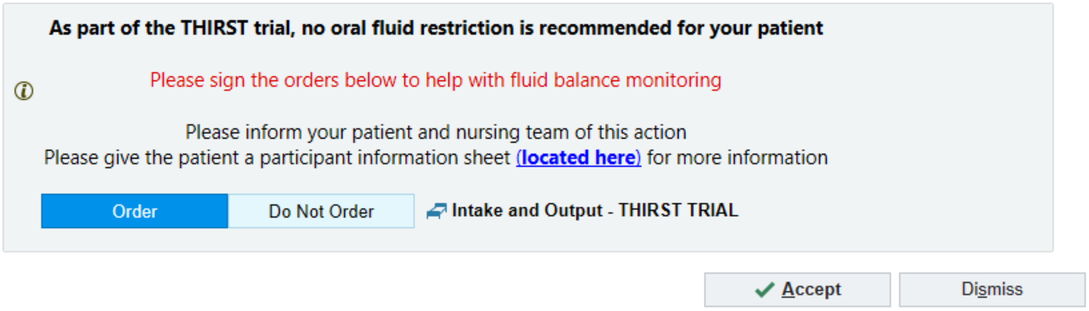


**Supplemental Figure 1c.** No oral fluid restriction recommendation

## Appendix 5 – Patient material

For peer review only

University College London Hospitals NHS
NHS Foundation Trust

# THIRST Alert participant information sheet

THIRST Alert

**We are inviting you to take part in a research project called the THIRST trial**
We wish to find out if restricting the total daily oral fluid intake in patients treated for fluid overload has any additional benefit to prescribing medications for patients with your condition of fluid overload. The THIRST trial will help to answer this question by providing randomised recommendations on oral fluid intake or no oral fluid intake using the electronic health care record system that the doctors and nurses use to manage your care.

**Why am I being asked to take part in this research?**
Your medical team have initiated medication for the treatment of fluid overload in the body but they are uncertain as to whether or not oral fluid restriction would be of additional benefit. After agreeing to include you in the study, your team will have received a randomised recommendation to either: fluid restriction of no more than 1 litre per day or continuing with your usual amount of oral fluid intake.

Although fluid restriction is a commonly used treatment in your condition, its effectiveness in helping to relieve symptoms is unclear. The mainstay of treatment remains medications to help remove excess water and this is unaffected by whether you restrict fluid or not.

Although you are unlikely to receive any direct benefit from taking part, the results of the study may improve the management of patients with a similar condition in the future.

**Do I have to take part?**
You do not have to take part in the study; you have the right to withdraw at any time. Your decision will not affect the quality of care you receive.

**What will I need to do if I take part?**
You do not need to do anything as a result of taking part. There are no additional tests or appointments. As part of your usual care, you will be asked how you are feeling and how thirsty you are. There are no extra visits to your doctor over and above those needed for your normal care. The study will last only for the time you are in hospital. The normal processes for your discharge and follow up are unaffected by the study.

**How do I opt out?**
If you would not like to be included in this study, please let the doctors and nurses looking after you know or alternatively contact the study team directly.

**What are the disadvantages/risks?**
There are no extra risks involved in taking part in this research. If at any moment you feel like you would like to drink more fluids, you can ask your usual care team for more.

**What will happen to information collected about me during the study?**
Your medical information will be kept strictly confidential by your doctor. The researchers will only be given as much information from your medical records as is needed for this research and that information will be anonymised. They will not be given your name, where you live or anything that could identify you. The results of this research will be made available to all those taking part who would like to receive it.


For peer review only - http://bmjopen.bmj.com/site/about/guidelines.xhtml

**Who is organising and funding the research?**
This study is being carried out by Dr Yang Chen, Dr Anoop Shah, Professor Folkert Asselbergs and Dr Tom Lumbers. It is sponsored by University College London and has support from the Clinical Research Informatics Unit of University College London Hospital (UCLH) and the Biomedical Research Centre at UCLH.

**Further Information**
You can ask your medical team any questions you may have about the study. You may also obtain more detailed information about this research, including how your medical information will be used, your privacy protected, and the compensation arrangements in the unlikely event that anything goes wrong from the following contacts:

**UCLH Patient Advice & Liaison Service (PALS)**
**Address**: PALS
Ground Floor Atrium
University College Hospital
235 Euston Road
London NW1 2BU
**Telephone** (main hospital): 02034473042
**Email:** Uclh.pals@nhs.net

**Study team contact details**:
Dr Yang Chen [Principal investigator: yang.chen@nhs.net]
Dr Tom Lumbers [Chief Investigator: tom.lumbers@nhs.net]
Cardiology Department at UCLH: 020 3447 8066

A description of the study is also available on the following UCLH website, and we plan to publish the study results there too: https://www.uclhospitals.brc.nihr.ac.uk/current-work

Thank you for reading this information.


For peer review only - http://bmjopen.bmj.com/site/about/guidelines.xhtml

For peer review only

University College London Hospitals NHS
NHS Foundation Trust

# Pragmatic research during routine care at UCLH - THIRST Alert trial

## A study of patients with fluid overload who may be suitable for fluid restriction

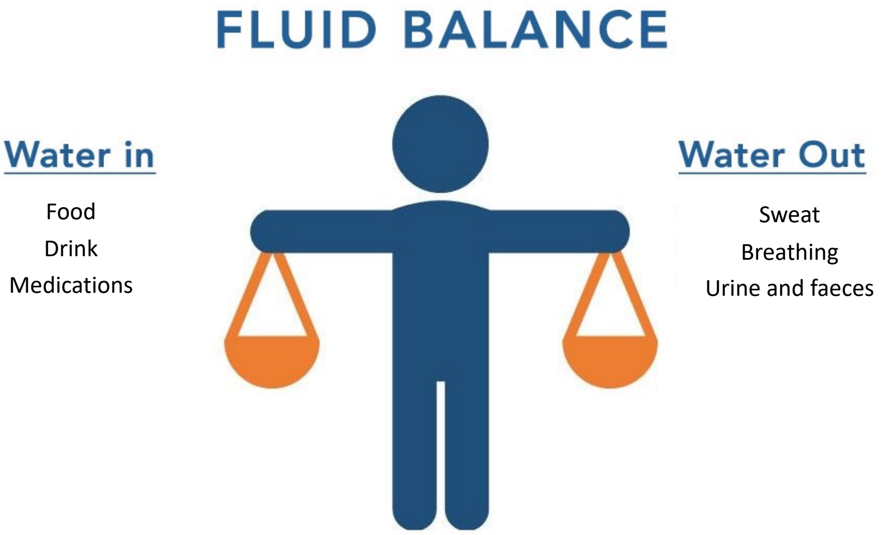


- Your medical team will inform you whether you are suitable for the study and if you will receive either (1) oral fluid restriction of up to 1 litre per day or (2) no restriction of oral fluids
- No other aspect of your care will be affected
- If you do not wish to take part, please let your clinical team know or contact the study team. Please see here for more information:

Study team and contact details:
Yang.chen@nhs.net - Clinical research fellow
Tom.lumbers@nhs.net - Consultant cardiologist
Cardiology Department at UCLH: 020 3447 8066

THIRST Alert

Study Poster Version No. 1.0 ………… Date…03 May 2023…………
IRAS 313551

For peer review only - http://bmjopen.bmj.com/site/about/guidelines.xhtml

STANDARD PROTOCOL ITEMS: RECOMMENDATIONS FOR INTERVENTIONAL TRIALS

SPIRIT 2013 Checklist: Recommended items to address in a clinical trial protocol and related documents*

| Section/item | Item No | Description | Addressed on page number |
|---|---|---|---|
| **Administrative information** | | | |
| Title | 1 | Descriptive title identifying the study design, population, interventions, and, if applicable, trial acronym | ______1_________ |
| Trial registration | 2a | Trial identifier and registry name. If not yet registered, name of intended registry | ______3_________ |
| | 2b | All items from the World Health Organization Trial Registration Data Set | _____ 3_______ |
| Protocol version | 3 | Date and version identifier | ______N/A_____ |
| Funding | 4 | Sources and types of financial, material, and other support | ______14_________ |
| Roles and responsibilities | 5a | Names, affiliations, and roles of protocol contributors | ______1________ |
| | 5b | Name and contact information for the trial sponsor | ______14_________ |
| | 5c | Role of study sponsor and funders, if any, in study design; collection, management, analysis, and interpretation of data; writing of the report; and the decision to submit the report for publication, including whether they will have ultimate authority over any of these activities | ______14________ |
| | 5d | Composition, roles, and responsibilities of the coordinating centre, steering committee, endpoint adjudication committee, data management team, and other individuals or groups overseeing the trial, if applicable (see Item 21a for data monitoring committee) | ______21________ |

For peer review only

**Introduction**

| | | | |
|---|---|---|---|
| Background and rationale | 6a | Description of research question and justification for undertaking the trial, including summary of relevant studies (published and unpublished) examining benefits and harms for each intervention | ____4-6______ |
| | 6b | Explanation for choice of comparators | ____5-6_______ |
| Objectives | 7 | Specific objectives or hypotheses | _____6_______ |
| Trial design | 8 | Description of trial design including type of trial (eg, parallel group, crossover, factorial, single group), allocation ratio, and framework (eg, superiority, equivalence, noninferiority, exploratory) | ____ 7______ |

**Methods: Participants, interventions, and outcomes**

| | | | |
|---|---|---|---|
| Study setting | 9 | Description of study settings (eg, community clinic, academic hospital) and list of countries where data will be collected. Reference to where list of study sites can be obtained | _____7________ |
| Eligibility criteria | 10 | Inclusion and exclusion criteria for participants. If applicable, eligibility criteria for study centres and individuals who will perform the interventions (eg, surgeons, psychotherapists) | __8 and Table 1 |
| Interventions | 11a | Interventions for each group with sufficient detail to allow replication, including how and when they will be administered | __8_and Figure S1 |
| | 11b | Criteria for discontinuing or modifying allocated interventions for a given trial participant (eg, drug dose change in response to harms, participant request, or improving/worsening disease) | ___8-9_______ |
| | 11c | Strategies to improve adherence to intervention protocols, and any procedures for monitoring adherence (eg, drug tablet return, laboratory tests) | ___NA_________ |
| | 11d | Relevant concomitant care and interventions that are permitted or prohibited during the trial | ___7__________ |
| Outcomes | 12 | Primary, secondary, and other outcomes, including the specific measurement variable (eg, systolic blood pressure), analysis metric (eg, change from baseline, final value, time to event), method of aggregation (eg, median, proportion), and time point for each outcome. Explanation of the clinical relevance of chosen efficacy and harm outcomes is strongly recommended | ___9________ |
| Participant timeline | 13 | Time schedule of enrolment, interventions (including any run-ins and washouts), assessments, and visits for participants. A schematic diagram is highly recommended (see Figure) | __Figure 1 and 9_ |



For peer review only - http://bmjopen.bmj.com/site/about/guidelines.xhtml

| | | | |
|---|---|---|---|
| Sample size | 14 | Estimated number of participants needed to achieve study objectives and how it was determined, including clinical and statistical assumptions supporting any sample size calculations | ___10__________ |
| Recruitment | 15 | Strategies for achieving adequate participant enrolment to reach target sample size | ___8_______ |

**Methods: Assignment of interventions (for controlled trials)**

Allocation:

| | | | |
|---|---|---|---|
| Sequence generation | 16a | Method of generating the allocation sequence (eg, computer-generated random numbers), and list of any factors for stratification. To reduce predictability of a random sequence, details of any planned restriction (eg, blocking) should be provided in a separate document that is unavailable to those who enrol participants or assign interventions | ___12________ |
| Allocation concealment mechanism | 16b | Mechanism of implementing the allocation sequence (eg, central telephone; sequentially numbered, opaque, sealed envelopes), describing any steps to conceal the sequence until interventions are assigned | ___9__________ |
| Implementation | 16c | Who will generate the allocation sequence, who will enrol participants, and who will assign participants to interventions | ____9_______ |
| Blinding (masking) | 17a | Who will be blinded after assignment to interventions (eg, trial participants, care providers, outcome assessors, data analysts), and how | ____9_________ |
| | 17b | If blinded, circumstances under which unblinding is permissible, and procedure for revealing a participant's allocated intervention during the trial | ___NA______ |

**Methods: Data collection, management, and analysis**

| | | | |
|---|---|---|---|
| Data collection methods | 18a | Plans for assessment and collection of outcome, baseline, and other trial data, including any related processes to promote data quality (eg, duplicate measurements, training of assessors) and a description of study instruments (eg, questionnaires, laboratory tests) along with their reliability and validity, if known. Reference to where data collection forms can be found, if not in the protocol | ____10_________ |
| | 18b | Plans to promote participant retention and complete follow-up, including list of any outcome data to be collected for participants who discontinue or deviate from intervention protocols | ____10_________ |



For peer review only - http://bmjopen.bmj.com/site/about/guidelines.xhtml

| | | | |
|---|---|---|---|
| Data management | 19 | Plans for data entry, coding, security, and storage, including any related processes to promote data quality (eg, double data entry; range checks for data values). Reference to where details of data management procedures can be found, if not in the protocol | ___10-11_______ |
| Statistical methods | 20a | Statistical methods for analysing primary and secondary outcomes. Reference to where other details of the statistical analysis plan can be found, if not in the protocol | ___10___________ |
| | 20b | Methods for any additional analyses (eg, subgroup and adjusted analyses) | ____NA______ |
| | 20c | Definition of analysis population relating to protocol non-adherence (eg, as randomised analysis), and any statistical methods to handle missing data (eg, multiple imputation) | ____NA_______ |
| **Methods: Monitoring** | | | |
| Data monitoring | 21a | Composition of data monitoring committee (DMC); summary of its role and reporting structure; statement of whether it is independent from the sponsor and competing interests; and reference to where further details about its charter can be found, if not in the protocol. Alternatively, an explanation of why a DMC is not needed | ____NA________ |
| | 21b | Description of any interim analyses and stopping guidelines, including who will have access to these interim results and make the final decision to terminate the trial | ____10-11_____ |
| Harms | 22 | Plans for collecting, assessing, reporting, and managing solicited and spontaneously reported adverse events and other unintended effects of trial interventions or trial conduct | ____11_________ |
| Auditing | 23 | Frequency and procedures for auditing trial conduct, if any, and whether the process will be independent from investigators and the sponsor | ____N/A_______ |
| **Ethics and dissemination** | | | |
| Research ethics approval | 24 | Plans for seeking research ethics committee/institutional review board (REC/IRB) approval | ____11_______ |
| Protocol amendments | 25 | Plans for communicating important protocol modifications (eg, changes to eligibility criteria, outcomes, analyses) to relevant parties (eg, investigators, REC/IRBs, trial participants, trial registries, journals, regulators) | ____11_________ |

| | | | |
|---|---|---|---|
| Consent or assent | 26a | Who will obtain informed consent or assent from potential trial participants or authorised surrogates, and how (see Item 32) | ______11_________ |
| | 26b | Additional consent provisions for collection and use of participant data and biological specimens in ancillary studies, if applicable | ______N/A_______ |
| Confidentiality | 27 | How personal information about potential and enrolled participants will be collected, shared, and maintained in order to protect confidentiality before, during, and after the trial | ______10________ |
| Declaration of interests | 28 | Financial and other competing interests for principal investigators for the overall trial and each study site | ______14_________ |
| Access to data | 29 | Statement of who will have access to the final trial dataset, and disclosure of contractual agreements that limit such access for investigators | ______11_________ |
| Ancillary and post-trial care | 30 | Provisions, if any, for ancillary and post-trial care, and for compensation to those who suffer harm from trial participation | ______N/A______ |
| Dissemination policy | 31a | Plans for investigators and sponsor to communicate trial results to participants, healthcare professionals, the public, and other relevant groups (eg, via publication, reporting in results databases, or other data sharing arrangements), including any publication restrictions | ______12_________ |
| | 31b | Authorship eligibility guidelines and any intended use of professional writers | ______14_________ |
| | 31c | Plans, if any, for granting public access to the full protocol, participant-level dataset, and statistical code | ______N/A_______ |
| **Appendices** | | | |
| Informed consent materials | 32 | Model consent form and other related documentation given to participants and authorised surrogates | _____Appendices |
| Biological specimens | 33 | Plans for collection, laboratory evaluation, and storage of biological specimens for genetic or molecular analysis in the current trial and for future use in ancillary studies, if applicable | ___N/A_________ |

*It is strongly recommended that this checklist be read in conjunction with the SPIRIT 2013 Explanation & Elaboration for important clarification on the items. Amendments to the protocol should be tracked and dated. The SPIRIT checklist is copyrighted by the SPIRIT Group under the Creative Commons "Attribution-NonCommercial-NoDerivs 3.0 Unported" license.

BMJ Open

# Rationale and design of the THIRST Alert feasibility study: a pragmatic, single centre, parallel group randomised controlled trial of an interruptive alert for oral fluid restriction in patients treated with intravenous furosemide

| | |
|---|---|
| Journal: | *BMJ Open* |
| Manuscript ID | bmjopen-2023-080410.R2 |
| Article Type: | Protocol |
| Date Submitted by the Author: | 08-Dec-2023 |
| Complete List of Authors: | Chen, Yang ; University College London, Institute of Health Informatics; University College London Hospitals NHS Foundation Trust, Clinical and Research Informatics Unit, NIHR UCLH Biomedical Research Centre<br>Shah, Anoop; University College London, Institute of Health Informatics; University College London Hospitals NHS Foundation Trust, Clinical and Research Informatics Unit, NIHR UCLH Biomedical Research Centre<br>Jani, Yogini; University College London Hospitals NHS Foundation Trust, Centre for Medicines Optimisation Research & Education - CMORE<br>Higgins, Daniel; University College London Hospitals NHS Foundation Trust, Clinical and Research Informatics Unit, NIHR UCLH Biomedical Research Centre<br>Saleem, Nausheen; University College London Hospitals NHS Foundation Trust, Clinical and Research Informatics Unit, NIHR UCLH Biomedical Research Centre<br>Chafer, Kris; University College London Hospitals NHS Foundation Trust, Clinical and Research Informatics Unit, NIHR UCLH Biomedical Research Centre<br>Sydes, Matthew; Medical Research Council Clinical Trials Unit at University College London, MRC Clinical Trials Unit, Institute of Clinical Trials and Methodology; Health Data Research UK,<br>Asselbergs, Folkert; University College London, Institute of Health Informatics; University of Amsterdam, Department of Cardiology, Amsterdam Cardiovascular Sciences, Amsterdam University Medical Centre<br>Lumbers, R Thomas; University College London, Institute of Health Informatics |
| <b>Primary Subject Heading</b>: | Cardiovascular medicine |
| Secondary Subject Heading: | Health informatics, Research methods |
| Keywords: | Heart failure < CARDIOLOGY, Randomized Controlled Trial, Feasibility Studies, Electronic Health Records |
| | |

SCHOLARONE™
Manuscripts

# Rationale and design of the THIRST Alert feasibility study: a pragmatic, single centre, parallel group randomised controlled trial of an interruptive alert for oral fluid restriction in patients treated with intravenous furosemide

Yang Chen[1,2]
Anoop D. Shah[1,2,]
Yogini Jani[3]
Daniel Higgins[2]
Nausheen Saleem[2]
Kris Chafer[2]
Matthew R Sydes[5,]
Folkert W Asselbergs[1,2,4]
R Thomas Lumbers[1]

1. Institute of Health Informatics, Faculty of Population Health Sciences, University College London, UK
2. Clinical and Research Informatics Unit, The National Institute for Health and Care Research University College London Hospitals Biomedical Research Centre, UCL, London, UK
3. Centre for Medicines Optimisation Research and Education, University College London Hospitals NHS Foundation Trust, London, UK
4. Department of Cardiology, Amsterdam Cardiovascular Sciences, Amsterdam University Medical Centre, University of Amsterdam, Amsterdam, Netherlands
5. MRC Clinical Trials Unit at UCL, Institute of Clinical Trials and Methodology, University College London, London, UK

**Corresponding author:** R. Thomas Lumbers

**E-mail:** t.lumbers@ucl.ac.uk

**Word count (**exc table/ figures/ legends/ acknowledgements**): 2222**

For peer review only

# Abstract

**Introduction**

Acute heart failure (HF) is a major cause of unplanned hospitalisation characterised by excess body water. A restriction in oral fluid intake is commonly imposed on patients as an adjunct to pharmacological therapy with loop diuretics, but there is a lack of evidence from traditional randomised controlled trials (RCTs) to support the safety and effectiveness of this intervention in the acute setting.

This study aims to explore the feasibility of using computer alerts within the electronic health record (EHR) system to invite clinical care teams to enrol patients into a pragmatic RCT at the time of clinical decision-making. It will additionally assess the effectiveness of using an alert to help address the clinical research question of whether oral fluid restriction is a safe and effective adjunct to pharmacological therapy for patients admitted with fluid overload.

**Methods and analysis**

THIRST Alert is a single centre, parallel group, open-label pragmatic RCT embedded in the EHR system that will be conducted as a feasibility study at an NHS hospital in London. The clinical care team will be invited to enrol suitable patients in the study using a point-of-care alert with a target sample size of 50 patients. Enrolled patients will then be randomised to either restricted or unrestricted oral fluid intake. Two primary outcomes will be explored: (1) the proportion of eligible patients enrolled in the study and (2) the mean difference in oral fluid intake between randomised groups. A series of secondary outcomes are specified

For peer review only

to evaluate the effectiveness of the alert, adherence to the randomised treatment allocation, and the quality of data generated from routine care, relevant to the outcomes of interest.

**Ethics and dissemination**

This study was approved by Riverside Research Ethics Committee (Ref: 22/LO/0889) and will be published on completion. Trial registration number NCT05869656.

**Keywords**

*Embedded trials; point-of-care randomisation; fluid overload; fluid restriction; heart failure*

**Strengths and Limitations of this study**

- THIRST Alert is a pragmatic randomised controlled trial where all elements of trial conduct are embedded within the routine care clinical pathway
- The intervention and programme theory have been co-designed with a multidisciplinary team including physicians, nurses, clinical informatics officers and patient representatives
- A proportionate consent model has been approved for use to help with the practicality of trial conduct
- Uncertainty remains regarding data quality in relation to the ascertainment of study outcomes from routine hospital care records.

For peer review only

# Introduction

Acute heart failure (HF) is an important cause of unplanned hospitalisation and is characterised by excess body water, also known as congestion. (1, 2) Despite being a common clinical problem, there is a lack of high-quality evidence to guide drug treatment and non-pharmacological measures. (3) Restriction of oral fluid intake is commonly used as an adjunct to pharmacological therapy but its effectiveness is uncertain and may exacerbate symptoms of thirst, as suggested by the limited randomised controlled trials which have been conducted to date (4–6). These previous trials achieved only small differences in oral fluid intake, which may reflect the Hawthorne effect whereby participation in a clinical trial influences fluid intake in both groups. (7)

Patients and clinicians identify the need for better treatment of fluid overload as a key priority, and the use of oral fluid restriction is also highlighted a gap in evidence in clinical practice guidelines (2, 8) (9). To address this evidence gap and some of the challenges encountered in previous studies, we designed a pragmatic RCT embedded in the electronic health record (EHR) system to evaluate the safety and effectiveness of oral fluid restriction in acute HF.

### Patient population

Fluid overload may be caused by liver or kidney dysfunction (10, 11), as well as by heart failure, with similar evidence gaps for congestion management in such circumstances. (12) (13) (14). However, a reliance on evaluating interventions for the immediate management of fluid overload only where the underlying cause is established may impact the

For peer review only - http://bmjopen.bmj.com/site/about/guidelines.xhtml

generalisability of any findings. Given that standardised clinical scores for congestion are not well validated or adopted in practice(15, 16), we used the prescription of two consecutive doses of intravenous furosemide within the first 48 hours of admission as evidence of a physician-assessed 'treatable trait' of fluid congestion, to define the study population. (17) At our proposed study site, more than one consecutive dose of intravenous furosemide was administered during the first 48 hours of admission in 1537 unplanned admissions between April 2019 and October 2022. In 56% of these admissions with available supporting data, oral fluid restriction was recommended (**Appendix 1**). The use of prescribing intention to identify the study population enabled digital enrolment into the study based on a discrete triggering event. To our knowledge, there has been no previous RCT conducted in all patients treated for congestion with intravenous furosemide in the acute care setting.

## Pragmatic research

There are many evidence gaps in routine clinical practice that have not been addressed by conventional randomised controlled trials which may be prohibitively expensive. Pragmatic clinical trials (PCT) offer a potential solution, particularly relevant to the evaluation of low-risk interventions where clinical equipoise may exist. By integrating a randomisation procedure into routine care pathways, automating case identification through the EHR, engaging the clinical care team for patient recruitment, and using routinely collected data for trial outcomes, there is the potential to perform large-scale trials at low-cost and low burden to patients and their caregivers. Many questions remain however, about how best to deliver PCTs embedded in EHR systems, including the design of physician-facing alerts and different models of consent. For our study, we employ an interruptive alert to provide the routine care team with an invitation to enrol eligible patients, and use a proportionate

For peer review only - http://bmjopen.bmj.com/site/about/guidelines.xhtml

process for consent that can be delivered by the same team.

PCTs have the potential to enable evidence generation during routine care, helping to conduct more efficient and representative trials.(18) Through examining the comparative effectiveness of existing interventions that have demonstrable variation in practice, many evidence gaps may be addressed. The use of oral fluid restriction in patients who are treated pharmacologically for fluid overload in the setting of acute unplanned care represents one such gap.

The THIRST Alert trial therefore aims to examine (1) whether interruptive alerts are an effective means to engage the usual care team in enrolling patients in an interventional pragmatic RCT; and (2), for enrolled patients, whether the randomised assignment to either oral fluid restriction to 1-litre per day or no oral fluid restriction leads to a difference in documented oral fluid intake. The evidence generated will be used to inform the design of subsequent multi-centre outcomes driven pragmatic clinical trials embedded in EHR systems.

For peer review only

For peer review only

For peer review only - http://bmjopen.bmj.com/site/about/guidelines.xhtml

# Methods and analysis

## Study design

THIRST Alert is a single centre, parallel group, open-label pragmatic RCT embedded in the EHR system that will be conducted as a feasibility study.(19) The study will be delivered entirely at University College Hospital, UCLH, a digitally mature hospital. (20) Enrolment started in May 2023 and the estimated trial completion date is December 2023. Given the low risk nature of the intervention, a verbal, opt-out consent model is used. The study protocol has been prepared with reference to the SPIRIT statement.(21) The trial is registered at ClinicalTrials.gov [NCT05869656]

## Study procedures

An overview of the study procedures is given in **Figure 1**. The trial was designed to be integrated into the electronic health record system EPIC (Epic, Epic Systems, Verona, WI). The screening and identification of eligible patients, display of interruptive alerts to physicians, randomisation and outcome ascertainment are all provisioned within EPIC. (22) Education events, internal communication and email correspondence were delivered to members of the routine care team to raise awareness of the study but no formal co-interventions were undertaken. The conduct and analysis of the trial is overseen by a trial management group who conceived a programme theory for the alert with relevant multidisciplinary input (**Appendix 2-3**). The enrolment invitation and randomised treatment recommendations are classified as complex interventions according to the Medical Research Council framework. (23)

For peer review only - http://bmjopen.bmj.com/site/about/guidelines.xhtml

The trial is designed to maximise the generalisability and applicability of the evidence generated and is highly pragmatic according to the PRECIS-2 framework (**Figure 2**). (24)

## Study participants

Adults of 18 years or older who were administered more than one dose of intravenous (IV) furosemide within the first 48 hours of an unplanned admission were eligible for the study. Patients admitted to a surgical, maternity or critical care ward were excluded **(Table 1)**.

## The interruptive alert inviting usual care team to enrol eligible patients

Any member of the clinical team may receive an interruptive alert (**Appendix 4, supplemental figure 1a**) during their interaction with EPIC for an eligible patient, informing them of the study and inviting them to enrol the patient. The clinician can click the appropriate button on the alert to enrol, decline to enrol, or defer the decision regarding enrolment. The alert is non-coercive, and the decision about patient enrolment is at the discretion of the treating clinician. We anticipate that physicians will enrol patients in cases where they judge that there is clinical equipoise as to whether oral fluid restriction is likely to benefit the patient. The alert was designed and tested with EPIC application specialists in an iterative manner, integrating feedback from end-users on the wording of the design.

## The randomised treatment intervention

After enrolment of patients in the study, the routine care team receives a recommendation regarding oral fluid intake and assignment is determined by point of care randomisation. The randomised treatment allocation is only presented to the clinical team after they click 'yes' to the THIRST alert, rather than displayed immediately, to minimise the possibility of

For peer review only

differential recruitment to the treatment arms of the trial. For patients allocated to the intervention group, the routine care team receive a recommendation for a target oral fluid restriction of 1 litre per day; for patients allocated to the control group, the care team receive a recommendation for unrestricted oral fluid intake (**Appendix 4, supplemental figure 1b and 1c**). The oral fluid restriction target was chosen based on previous RCTs and acceptability in clinical practice (4–6). The randomised treatment recommendation for both groups additionally advises the clinician to: (1) complete an EPIC order for fluid balance monitoring; (2) document the treatment allocation in the clinical notes (3) inform the patient and the nursing team and (4) provide the patient with a participant information sheet.

## Randomisation and blinding

Subjects will be randomised 1:1 using an internal random number rule implemented using a standard EPIC randomisation tool used in other PCTs.(25) No block randomisation or additional covariate balancing will be undertaken. The study is open label with patients and their care teams being made aware of the treatment allocation.

## Study Outcomes

### Primary outcomes

1. Number of eligible patients randomised
2. Difference in oral fluid intake between intervention and control arms

### Secondary outcomes

1. Adherence to randomised treatment recommendation
2. Proportion of alerts resulting in clinical orders for nursing care
3. Oral fluid intake
4. Net fluid balance
5. Weight change after randomisation
6. Length of stay

For peer review only - http://bmjopen.bmj.com/site/about/guidelines.xhtml

7. Frequency of blood test measurements of renal function
8. Prescription of diuretic medications
9. Daily change in Creatinine
10. Patient Reported Outcome Measures

These outcomes were chosen based on clinical relevance and the ability to detect the possibility of attributable harm during an inpatient admission.

## Sample size and power calculation

No formal sample size calculation was performed given the primary outcomes pertain to the feasibility of using an EHR alert aimed at the routine care team to (i) enrol eligible patients into an RCT of oral fluid restriction and (ii) cause and sustain the allocated clinical treatment effect, as measured by documented oral fluid intake. This is in keeping with the role of feasibility studies used by other groups. (26) Our target sample size was 50 patients, based on the predicted number of eligible patients at the study site during the recruitment period.

## Data collection, curation and storage

Baseline characteristics and study outcomes will be extracted from the routine care record. Information on the frequency of the interruptive alert and staff responses to alerts will be recorded in EPIC. We will also extract primary and secondary ICD-10 diagnosis codes assigned to the clinical episode by the clinical coding team. Study data will be transferred to a secure research environment within UCLH NHS Foundation Trust as pseudo-anonymised electronic Case Report Forms (eCRFs)**.** No data will leave the UCLH NHS system. Patients who opt out or withdraw from the trial will have their decision documented on the eCRF and their data will not be included in the main analysis.

For peer review only

## Statistical analysis plan

Baseline characteristics of study participants will be summarised using percentages or means and standard deviations as appropriate and compared using t tests for continuous variables (expressed as mean ± standard deviation) and the $\chi^2$ tests or the Fisher's exact tests for categorical variables (expressed as count and percentage). Secondary endpoints for intervention and control groups will be compared using $\chi^2$ test for binary outcomes, and T-test for continuous outcomes. For subgroup analyses, linear or logistic regression will be used to determine the effects of treatment allocation according to heart failure status. No adjustment or imputation will be used for missing data. For this feasibility study, we considered a sample of 20 participants enrolled as a minimum number acceptable for feasibility and a daily mean difference of 250ml of fluid intake between treatment arms as clinically relevant. Progression to a multi-centre clinical outcomes trial will be contingent on a series of stop/ go criteria based on achieving the primary outcome targets and on secondary outcomes relating to data quality.

## Monitoring and Evaluation

The trial will report serious adverse events (SAEs) that are attributable to the study through the EHR, in accordance with the safety reporting processes and sponsor policy. (27) The Trial Management Group will review recruitment rates, SAEs and any substantial amendments to the protocol.

## Patient and public involvement (PPI)

Patients were integrally involved in the design of the study as previously reported. (28) In brief this involved a patient participation event where options for consent and outcome

For peer review only

measures were explored which subsequently informed the study design. In accordance with the National Institute for Health Research guidance on co-production(29), one participant (KC) served as a patient advisor to the trial and reviewed all trial materials.

## Ethics and Dissemination

This study protocol was approved by the London Riverside Research Ethics Committee (Ref: 22/LO/0889) and sponsored by University College London (Ref: 151938). A verbal opt-out model of consent was adopted based on the negligible risk associated with the intervention. Patients are informed of their study participation and of their treatment allocation and can opt-out of the study or decline to follow the recommendation for oral fluid restriction at any time.  A copy of the sample participant information sheet (PIS) is provided to patients (**Appendix 5**). The data management for the trial conforms to NHS Information standards DCB0129 and DCB0160 and was approved by University College London Hospitals NHS Foundation Trust (UCLH) Digital Services Safety board. (30) The alert is not classified as a separate piece of software but is incorporated within the Epic electronic health record, which is registered as a Class I medical device by the UK Medicines and Healthcare products Regulatory Agency. (31, 32) The study findings will be disseminated through publication in open-access peer-reviewed journals and through the provision of reports for patients and clinicians involved in the study. As a feasibility study, the findings shared will include trial evaluation, and whether alerts which 'nudge' clinicians to recruit patients into pragmatic, low-risk comparative effectiveness trials (33), are a scalable way to conduct efficient

For peer review only

RCTs.(18) The THIRST Alert feasibility study will share insights that inform the design and execution of future integrated approaches to evidence generation from routine care.

## Author contributions

YC, AS, MS, FA, and TL conceived the study idea. YC and TL wrote the first draft of the protocol. YJ, DH and NS helped to design, test and review the study alert. KC reviewed and edited the patient-facing study materials. All authors reviewed the manuscript and contributed to revisions.

## Acknowledgements

We are grateful to clinical colleagues, Inaki Elizondo, Pamela Stephenson, Sian Roberts-Walsh, Alexander Tranter and Ali Hosin for assistance with the study design. We thank Steve Harris and Pier Lambiase for comments on the study protocol and to members of the digital safety committee with the safe implementation of this study.

## Funding statement

This work was supported by the National Institute for Health and Care Research (NIHR) UCLH Biomedical Research Centre (BRC), award number NIHR-INF-1873. The study funder did not play any role in the design of the study.

## Conflict of interests

R.T.L has received funding from Pfizer for unrelated research, under the Innovative Target Exploration Network programme.

For peer review only

For peer review only

# References


1. Savarese G, Becher PM, Lund LH, Seferovic P, Rosano GMC, Coats A. Global burden of heart failure: A comprehensive and updated review of epidemiology. Cardiovasc Res. 2022; cvac013.

2. McDonagh TA, Metra M, Adamo M, Gardner RS, Baumbach A, Böhm M, et al. 2021 ESC Guidelines for the diagnosis and treatment of acute and chronic heart failure. European Heart Journal. 2021; 42(36):3599–726.

3. Koratala A, Ronco C, Kazory A. Diagnosis of Fluid Overload: From Conventional to Contemporary Concepts. Cardiorenal Med. 2022; 12(4):141–54.

4. Aliti GB, Rabelo ER, Clausell N, Rohde LE, Biolo A, Beck-da-Silva L. Aggressive fluid and sodium restriction in acute decompensated heart failure: a randomized clinical trial. JAMA Intern Med. 2013; 173(12):1058–64.

5. Machado d'Almeida KS, Rabelo-Silva ER, Souza GC, Trojahn MM, Santin Barilli SL, Aliti G, et al. Aggressive fluid and sodium restriction in decompensated heart failure with preserved ejection fraction: Results from a randomized clinical trial. Nutrition. 2018; 54:111–7.

6. Travers B, O'Loughlin C, Murphy NF, Ryder M, Conlon C, Ledwidge M, et al. Fluid restriction in the management of decompensated heart failure: no impact on time to clinical stability. J Card Fail. 2007; 13(2):128–32.

7. Sedgwick P, Greenwood N. Understanding the Hawthorne effect. BMJ. 2015; h4672.

8. Heidenreich PA, Bozkurt B, Aguilar D, Allen LA, Byun JJ, Colvin MM, et al. 2022 ACC/AHA/HFSA Guideline for the Management of Heart Failure. Journal of Cardiac Failure. 2022; 28(5):e1–167.

9. Taylor CJ, Huntley AL, Burden J, Gadoud A, Gronlund T, Jones NR, et al. Research priorities in advanced heart failure: James Lind alliance priority setting partnership. Open Heart. 2020; 7(1):e001258.

10. Damman K, Voors AA, Hillege HL, Navis G, Lechat P, Van Veldhuisen DJ, et al. Congestion in chronic systolic heart failure is related to renal dysfunction and increased mortality. European Journal of Heart Failure. 2010; 12(9):974–82.

11. Naschitz JE, Slobodin G, Lewis RJ, Zuckerman E, Yeshurun D. Heart diseases affecting the liver and liver diseases affecting the heart. American Heart Journal. 2000; 140(1):111–20.

12. Aithal GP, Palaniyappan N, China L, Härmälä S, Macken L, Ryan JM, et al. Guidelines on the management of ascites in cirrhosis. Gut. 2021; 70(1):9–29.

13. NICE guideline NG107. RRT and conservative management Evidence review for dietary management and fluid restriction [Internet]. [date unknown]. Available from:


For peer review only - http://bmjopen.bmj.com/site/about/guidelines.xhtml

https://www.nice.org.uk/guidance/ng107/evidence/i-dietary-management-and-fluid-restriction-pdf-235524385620.

14. Nice guideline NG203. Chronic kidney disease: assessment and management [Internet]. [date unknown]. Available from: https://www.nice.org.uk/guidance/ng203/resources/chronic-kidney-disease-assessment-and-management-pdf-66143713055173.

15. Boorsma EM, Maaten JM ter, Damman K, Dinh W, Gustafsson F, Goldsmith S, et al. Congestion in heart failure: a contemporary look at physiology, diagnosis and treatment. Nat Rev Cardiol. 2020; 17(10):641–55.

16. Gheorghiade M, Follath F, Ponikowski P, Barsuk JH, Blair JEA, Cleland JG, et al. Assessing and grading congestion in acute heart failure: a scientific statement from the acute heart failure committee of the heart failure association of the European Society of Cardiology and endorsed by the European Society of Intensive Care Medicine. Eur J Heart Fail. 2010; 12(5):423–33.

17. Agusti A, Bel E, Thomas M, Vogelmeier C, Brusselle G, Holgate S, et al. Treatable traits: toward precision medicine of chronic airway diseases. Eur Respir J. 2016; 47(2):410–9.

18. Usman MS, Van Spall HGC, Greene SJ, Pandey A, McGuire DK, Ali ZA, et al. The need for increased pragmatism in cardiovascular clinical trials. Nat Rev Cardiol [Internet]. 2022 [cited 2022 Sep 1]. Available from: https://www.nature.com/articles/s41569-022-00705-w.

19. Eldridge SM, Lancaster GA, Campbell MJ, Thabane L, Hopewell S, Coleman CL, et al. Defining Feasibility and Pilot Studies in Preparation for Randomised Controlled Trials: Development of a Conceptual Framework. PLoS ONE. 2016; 11(3):e0150205.

20. NHS England. Acute global digital exemplars. (online) [Internet]. 2022 [cited 2022 May 17]. Available from: https://www.england.nhs.uk/digitaltechnology/connecteddigitalsystems/exemplars/acute-global-digital-exemplars/.

21. Chan A-W, Tetzlaff JM, Altman DG, Laupacis A, Gøtzsche PC, Krleža-Jerić K, et al. SPIRIT 2013 statement: defining standard protocol items for clinical trials. Ann Intern Med. 2013; 158(3):200–7.

22. Latha M, Bellary S, Krishnankutty B. Basics of case report form designing in clinical research. Perspect Clin Res. 2014; 5(4):159.

23. Skivington K, Matthews L, Simpson SA, Craig P, Baird J, Blazeby JM, et al. A new framework for developing and evaluating complex interventions: update of Medical Research Council guidance. BMJ. 2021; n2061.

24. Loudon K, Treweek S, Sullivan F, Donnan P, Thorpe KE, Zwarenstein M. The PRECIS-2 tool: designing trials that are fit for purpose. BMJ. 2015; 350:h2147.

For peer review only

25. Wilson FP, Martin M, Yamamoto Y, Partridge C, Moreira E, Arora T, et al. Electronic health record alerts for acute kidney injury: multicenter, randomized clinical trial. BMJ. England; 2021; 372(8900488, bmj, 101090866):m4786.

26. Blatch-Jones AJ, Pek W, Kirkpatrick E, Ashton-Key M. Role of feasibility and pilot studies in randomised controlled trials: a cross-sectional study. BMJ Open. 2018; 8(9):e022233.

27. NHS Health Research Authority. Safety and progress reports (other research) procedural table. (Online). [Internet]. [cited 2023 May 10]. Available from: https://www.hra.nhs.uk/approvals-amendments/managing-your-approval/safety-reporting/safety-and-progress-reports-other-research-procedural-table/.

28. Chen Y, Hosin AA, George MJ, Asselbergs FW, Shah AD. Digital technology and patient and public involvement (PPI) in routine care and clinical research—A pilot study. PLoS ONE. 2023; 18(2):e0278260.

39. Staniszewska S, Denegri S, Matthews R, Minogue V. Reviewing progress in public involvement in NIHR research: developing and implementing a new vision for the future. BMJ Open. 2018; 8(7):e017124.

30. NHS Digital. DCB0160: Clinical Risk Management: its Application in the Deployment and Use of Health IT Systems [Internet]. 2023 [cited 2023 Feb 3]. Available from: https://digital.nhs.uk/data-and-information/information-standards/information-standards-and-data-collections-including-extractions/publications-and-notifications/standards-and-collections/dcb0160-clinical-risk-management-its-application-in-the-deployment-and-use-of-health-it-systems.

31. Medicines and Healthcare products Regulatory Agency. Public Access Registration Database [Internet]. 2023 [cited 2023 Sep 27]. Available from: (https://pard.mhra.gov.uk/manufacturer-details/33914.

32. Medicines and Healthcare products Regulatory Agency. Guidance:Medical devicee stand-alone including apps (including IVDMDs) [Internet]. 2023 [cited 2023 Feb 3]. Available from: https://assets.publishing.service.gov.uk/government/uploads/system/uploads/attachment_data/file/1105233/Medical_device_stand-alone_software_including_apps.pdf.

33. Chen Y, Harris S, Rogers Y, Ahmad T, Asselbergs FW. Nudging within learning health systems: next generation decision support to improve cardiovascular care. Eur Heart J. 2022; 43(13):1296–306.

For peer review only - http://bmjopen.bmj.com/site/about/guidelines.xhtml

## Table and Figure Legends

| Study participants | Eligibility Criteria |
|---|---|
| Patients | **Inclusion**<br>- Aged 18 years and over<br>- Prescribed IV furosemide during the first 48h of their admission as a regular prescription rather than a one-off dose.<br>- Assessed as being suitable for inclusion in the trial by the responsible clinical team i.e., fluid restriction deemed to be in equipoise.<br>**Exclusion**<br>- Surgical or obstetric wards<br>- Critical care wards |
| Prescribing clinicians: Physicians (Consultants and Junior Doctors) | Any clinician with prescribing rights and who prescribes more than one dose of IV furosemide within 48 hours of admission, during the trial recruitment period. |
| Nursing staff | A separate order is triggered for enrolled patients and only presented to nursing staff who access their patient record to help facilitate fluid balance documentation |

**Table 1.** Eligibility criteria for THIRST Alert study participants

**Figure 1.** THIRST Alert trial patient flow

**Figure 2.** PRECIS-2 score for THIRST Alert trial. Each of the nine domains are scored on a 5-point Likert continuum (from 1=very explanatory “ideal conditions” to 5=very pragmatic “usual care conditions”)

For peer review only

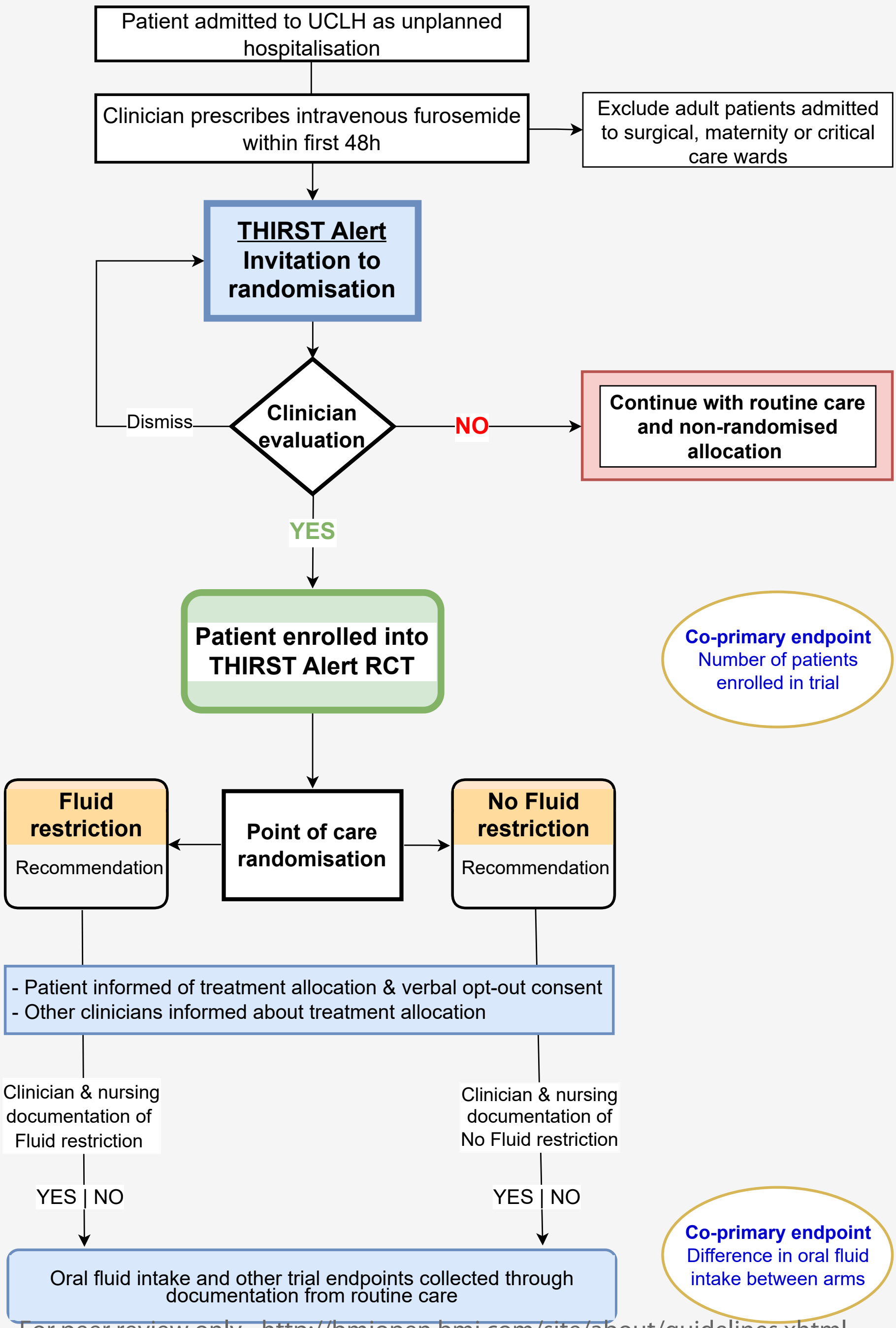


For peer review only - http://bmjopen.bmj.com/site/about/guidelines.xhtml

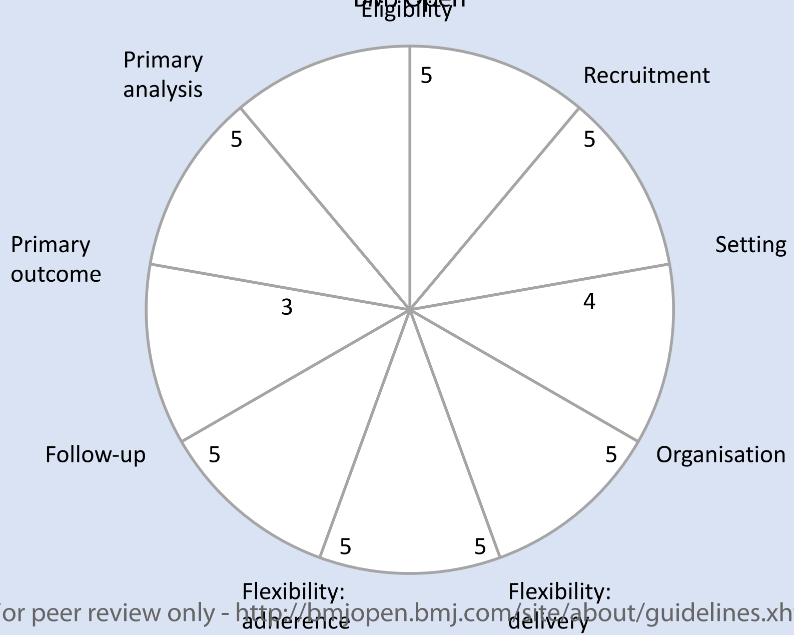


For peer review only - http://bmjopen.bmj.com/site/about/guidelines.xhtml

## Appendix 1 – Fluid restriction at study site

| Fluid restriction status | Total (%) | Primary diagnosis of Heart failure (%) | Heart failure as co-morbidity (%) | No diagnosis of Heart failure (%) |
|---|---|---|---|---|
| **Received fluid restriction at least once** | 826 (56) | 373 (71) | 294 (55) | 159 (38) |
| **Did not receive fluid restriction** | 661 (44) | 152 (29) | 244 (45) | 265 (62) |

**Table S1.** Fluid restriction treatment strategy at the study site prior to trial implementation. Data shown is from unplanned admissions at University College Hospital between April 2019 – October 2022 where patients were prescribed IV furosemide within 48 hours of admission. The total number of patients was 1537 of data was available on heart failure and fluid restriction status in 1487.

## Appendix 2 - Trial management group

Dr. Yang Chen (Principal Investigator) – Clinical Research Fellow & Cardiology Registrar

Dr. Tom Lumbers (Chief Investigator) – Associate Professor & Consultant Cardiologist

Dr. Anoop Shah – Associate Professor & Consultant Clinical Pharmacologist

Prof. Matthew Sydes m.sydes@ucl.ac.uk – Professor of Clinical Trials & Methodology

Prof. Folkert W. Asselbergs, f.asselbergs@ucl.ac.uk - Professor of Precision Medicine

For peer review only

## Appendix 3 – Programme Theory for THIRST Alert

For peer review only

| Event | Trigger | Action |
|---|---|---|
| **1.** Patient identification | Based on a *regular or continuous IV* furosemide prescription within 48 hours of unplanned admission | First alert is presented to prescribing clinician after triggering action is performed, most likely in emergency department or acute assessment unit |
| **2.** Alert Presentation | Based on trigger of 'Open Patient Chart' and 'Open Orders Sidebar' | Clinician accepts randomisation – patient enrolled and status on EHR updated to 'research participant' visible to all staff |
| | | Clinician rejects randomisation – patient receives a non-randomised treatment recommendation for fluid restriction or no restriction, in keeping with usual care. |
| | | Clinician defers – alert represented after another trigger action performed by any prescribing clinician within 48 hours |
| **3.** Alert recommendation | Based on clicking 'Yes' to first alert | Fluid restriction alert |
| | | No Fluid restriction alert |
| **4.** Documented evidence of treatment allocation and communication to nursing staff and patient | Alert prompts user to link to document in patient notes. | Documentation of treatment allocation occurs in same manner as routine care. Enrolled patients have an additional workflow within the EHR for nursing staff to document fluid balance |
| | Alert prompts staff to provide additional participant information sheet | A paper PIS is available on all anticipated ward locations and a link supplied to print off a digital copy |
| **5.** Documentation of fluid intake, and other secondary trial outcomes: e.g., fluid balance, weight and patient experience | Prompted by nursing workflow, medical workflow and usual care processes | No additional changes to existing routine care processes |
| **6.** Patient is no longer fluid overloaded | Patient's clinical status has changed and their medical therapy has been de-escalated | No change to usual care processes with regards to continuation or cessation of fluid restriction arm. Trial ends at the end of the admission episode |

**Table S2**. Programme theory for THIRST Alert trial. Steps 1-6 represent a summary of the logic model considered for the trial intervention and its downstream effects

For peer review only

## Appendix 4 – Alert Design

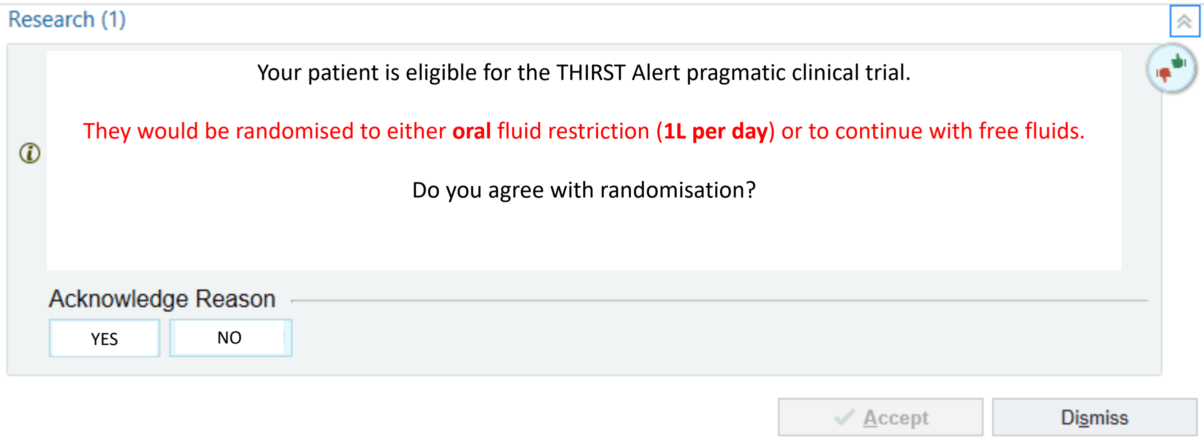


**Supplemental Figure 1a.** THIRST Alert invitation to enrol

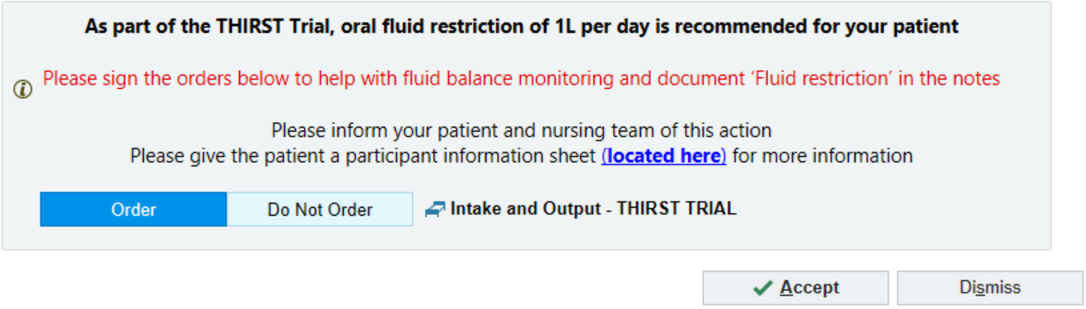


**Supplemental Figure 1b.** Oral fluid restriction recommendation

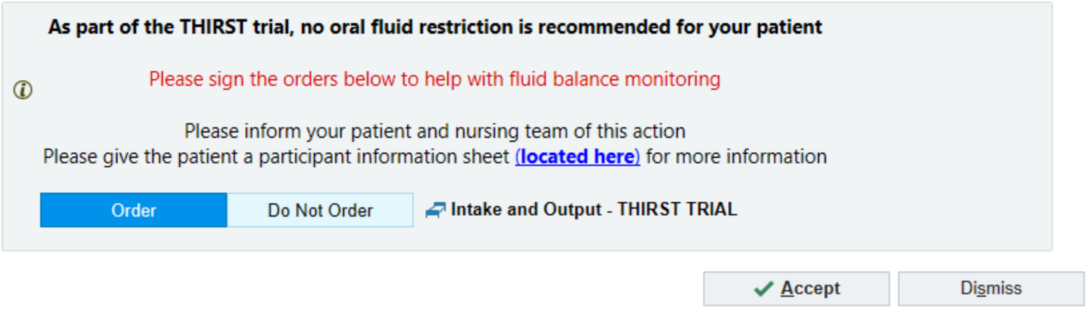


**Supplemental Figure 1c.** No oral fluid restriction recommendation

## Appendix 5 – Patient material

For peer review only

University College London Hospitals NHS
NHS Foundation Trust

# THIRST Alert participant information sheet

THIRST Alert

**We are inviting you to take part in a research project called the THIRST trial**
We wish to find out if restricting the total daily oral fluid intake in patients treated for fluid overload has any additional benefit to prescribing medications for patients with your condition of fluid overload. The THIRST trial will help to answer this question by providing randomised recommendations on oral fluid intake or no oral fluid intake using the electronic health care record system that the doctors and nurses use to manage your care.

**Why am I being asked to take part in this research?**
Your medical team have initiated medication for the treatment of fluid overload in the body but they are uncertain as to whether or not oral fluid restriction would be of additional benefit. After agreeing to include you in the study, your team will have received a randomised recommendation to either: fluid restriction of no more than 1 litre per day or continuing with your usual amount of oral fluid intake.

Although fluid restriction is a commonly used treatment in your condition, its effectiveness in helping to relieve symptoms is unclear. The mainstay of treatment remains medications to help remove excess water and this is unaffected by whether you restrict fluid or not.

Although you are unlikely to receive any direct benefit from taking part, the results of the study may improve the management of patients with a similar condition in the future.

**Do I have to take part?**
You do not have to take part in the study; you have the right to withdraw at any time. Your decision will not affect the quality of care you receive.

**What will I need to do if I take part?**
You do not need to do anything as a result of taking part. There are no additional tests or appointments. As part of your usual care, you will be asked how you are feeling and how thirsty you are. There are no extra visits to your doctor over and above those needed for your normal care. The study will last only for the time you are in hospital. The normal processes for your discharge and follow up are unaffected by the study.

**How do I opt out?**
If you would not like to be included in this study, please let the doctors and nurses looking after you know or alternatively contact the study team directly.

**What are the disadvantages/risks?**
There are no extra risks involved in taking part in this research. If at any moment you feel like you would like to drink more fluids, you can ask your usual care team for more.

**What will happen to information collected about me during the study?**
Your medical information will be kept strictly confidential by your doctor. The researchers will only be given as much information from your medical records as is needed for this research and that information will be anonymised. They will not be given your name, where you live or anything that could identify you. The results of this research will be made available to all those taking part who would like to receive it.


For peer review only - http://bmjopen.bmj.com/site/about/guidelines.xhtml

**Who is organising and funding the research?**
This study is being carried out by Dr Yang Chen, Dr Anoop Shah, Professor Folkert Asselbergs and Dr Tom Lumbers. It is sponsored by University College London and has support from the Clinical Research Informatics Unit of University College London Hospital (UCLH) and the Biomedical Research Centre at UCLH.

**Further Information**
You can ask your medical team any questions you may have about the study. You may also obtain more detailed information about this research, including how your medical information will be used, your privacy protected, and the compensation arrangements in the unlikely event that anything goes wrong from the following contacts:

**UCLH Patient Advice & Liaison Service (PALS)**
**Address**: PALS
Ground Floor Atrium
University College Hospital
235 Euston Road
London NW1 2BU
**Telephone** (main hospital): 02034473042
**Email:** Uclh.pals@nhs.net

**Study team contact details**:
Dr Yang Chen [Principal investigator: yang.chen@nhs.net]
Dr Tom Lumbers [Chief Investigator: tom.lumbers@nhs.net]
Cardiology Department at UCLH: 020 3447 8066

A description of the study is also available on the following UCLH website, and we plan to publish the study results there too: https://www.uclhospitals.brc.nihr.ac.uk/current-work

THIRST Alert

Thank you for reading this information.

For peer review only


For peer review only - http://bmjopen.bmj.com/site/about/guidelines.xhtml

University College London Hospitals NHS
NHS Foundation Trust

# Pragmatic research during routine care at UCLH - THIRST Alert trial

## A study of patients with fluid overload who may be suitable for fluid restriction

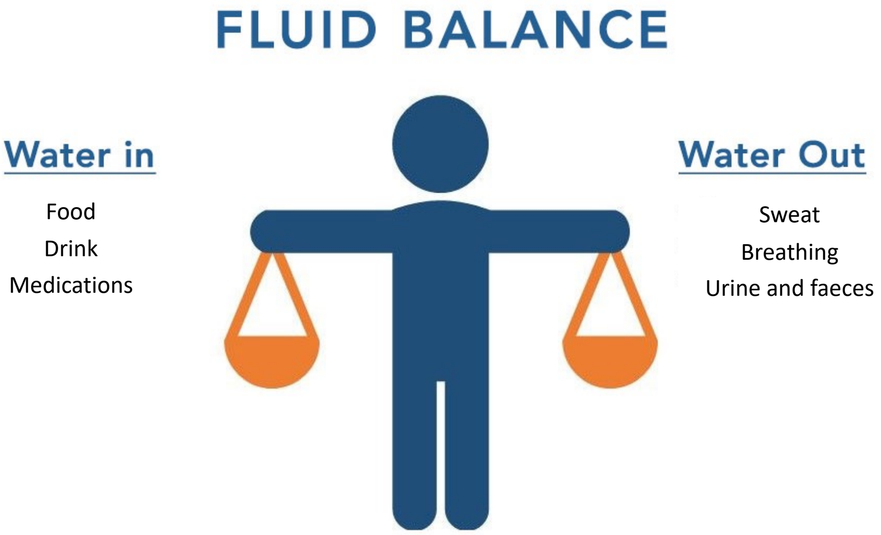


- Your medical team will inform you whether you are suitable for the study and if you will receive either (1) oral fluid restriction of up to 1 litre per day or (2) no restriction of oral fluids
- No other aspect of your care will be affected
- If you do not wish to take part, please let your clinical team know or contact the study team. Please see here for more information:

Study team and contact details:
Yang.chen@nhs.net - Clinical research fellow
Tom.lumbers@nhs.net - Consultant cardiologist
Cardiology Department at UCLH: 020 3447 8066

THIRST Alert

Study Poster Version No. 1.0 ………… Date…03 May 2023…………
IRAS 313551

For peer review only - http://bmjopen.bmj.com/site/about/guidelines.xhtml

SPIRIT
STANDARD PROTOCOL ITEMS: RECOMMENDATIONS FOR INTERVENTIONAL TRIALS

SPIRIT 2013 Checklist: Recommended items to address in a clinical trial protocol and related documents*

| Section/item | Item No | Description | Addressed on page number |
|---|---|---|---|
| **Administrative information** | | | |
| Title | 1 | Descriptive title identifying the study design, population, interventions, and, if applicable, trial acronym | _____1________ |
| Trial registration | 2a | Trial identifier and registry name. If not yet registered, name of intended registry | _____3________ |
| | 2b | All items from the World Health Organization Trial Registration Data Set | ____ 3______ |
| Protocol version | 3 | Date and version identifier | _____N/A____ |
| Funding | 4 | Sources and types of financial, material, and other support | _____14________ |
| Roles and responsibilities | 5a | Names, affiliations, and roles of protocol contributors | _____1_______ |
| | 5b | Name and contact information for the trial sponsor | _____14________ |
| | 5c | Role of study sponsor and funders, if any, in study design; collection, management, analysis, and interpretation of data; writing of the report; and the decision to submit the report for publication, including whether they will have ultimate authority over any of these activities | _____14_______ |
| | 5d | Composition, roles, and responsibilities of the coordinating centre, steering committee, endpoint adjudication committee, data management team, and other individuals or groups overseeing the trial, if applicable (see Item 21a for data monitoring committee) | _____21_______ |

For peer review only

| | | | |
|---|---|---|---|
| **Introduction** | | | |
| Background and rationale | 6a | Description of research question and justification for undertaking the trial, including summary of relevant studies (published and unpublished) examining benefits and harms for each intervention | ____4-6______ |
| | 6b | Explanation for choice of comparators | ____5-6_______ |
| Objectives | 7 | Specific objectives or hypotheses | _____6_______ |
| Trial design | 8 | Description of trial design including type of trial (eg, parallel group, crossover, factorial, single group), allocation ratio, and framework (eg, superiority, equivalence, noninferiority, exploratory) | ____ 7______ |
| **Methods: Participants, interventions, and outcomes** | | | |
| Study setting | 9 | Description of study settings (eg, community clinic, academic hospital) and list of countries where data will be collected. Reference to where list of study sites can be obtained | _____7________ |
| Eligibility criteria | 10 | Inclusion and exclusion criteria for participants. If applicable, eligibility criteria for study centres and individuals who will perform the interventions (eg, surgeons, psychotherapists) | __8 and Table 1 |
| Interventions | 11a | Interventions for each group with sufficient detail to allow replication, including how and when they will be administered | __8_and Figure S1 |
| | 11b | Criteria for discontinuing or modifying allocated interventions for a given trial participant (eg, drug dose change in response to harms, participant request, or improving/worsening disease) | ___8-9_______ |
| | 11c | Strategies to improve adherence to intervention protocols, and any procedures for monitoring adherence (eg, drug tablet return, laboratory tests) | ___NA_________ |
| | 11d | Relevant concomitant care and interventions that are permitted or prohibited during the trial | ___7__________ |
| Outcomes | 12 | Primary, secondary, and other outcomes, including the specific measurement variable (eg, systolic blood pressure), analysis metric (eg, change from baseline, final value, time to event), method of aggregation (eg, median, proportion), and time point for each outcome. Explanation of the clinical relevance of chosen efficacy and harm outcomes is strongly recommended | ___9________ |
| Participant timeline | 13 | Time schedule of enrolment, interventions (including any run-ins and washouts), assessments, and visits for participants. A schematic diagram is highly recommended (see Figure) | __Figure 1 and 9_ |



For peer review only - http://bmjopen.bmj.com/site/about/guidelines.xhtml

| | | | |
|---|---|---|---|
| Sample size | 14 | Estimated number of participants needed to achieve study objectives and how it was determined, including clinical and statistical assumptions supporting any sample size calculations | ___10__________ |
| Recruitment | 15 | Strategies for achieving adequate participant enrolment to reach target sample size | ___8_______ |
| **Methods: Assignment of interventions (for controlled trials)** | | | |
| Allocation: | | | |
| Sequence generation | 16a | Method of generating the allocation sequence (eg, computer-generated random numbers), and list of any factors for stratification. To reduce predictability of a random sequence, details of any planned restriction (eg, blocking) should be provided in a separate document that is unavailable to those who enrol participants or assign interventions | ___12________ |
| Allocation concealment mechanism | 16b | Mechanism of implementing the allocation sequence (eg, central telephone; sequentially numbered, opaque, sealed envelopes), describing any steps to conceal the sequence until interventions are assigned | ___9__________ |
| Implementation | 16c | Who will generate the allocation sequence, who will enrol participants, and who will assign participants to interventions | ____9_______ |
| Blinding (masking) | 17a | Who will be blinded after assignment to interventions (eg, trial participants, care providers, outcome assessors, data analysts), and how | ____9_________ |
| | 17b | If blinded, circumstances under which unblinding is permissible, and procedure for revealing a participant's allocated intervention during the trial | ___NA______ |
| **Methods: Data collection, management, and analysis** | | | |
| Data collection methods | 18a | Plans for assessment and collection of outcome, baseline, and other trial data, including any related processes to promote data quality (eg, duplicate measurements, training of assessors) and a description of study instruments (eg, questionnaires, laboratory tests) along with their reliability and validity, if known. Reference to where data collection forms can be found, if not in the protocol | ____10_________ |
| | 18b | Plans to promote participant retention and complete follow-up, including list of any outcome data to be collected for participants who discontinue or deviate from intervention protocols | ____10_________ |



For peer review only - http://bmjopen.bmj.com/site/about/guidelines.xhtml

| | | | |
|---|---|---|---|
| Data management | 19 | Plans for data entry, coding, security, and storage, including any related processes to promote data quality (eg, double data entry; range checks for data values). Reference to where details of data management procedures can be found, if not in the protocol | ___10-11_______ |
| Statistical methods | 20a | Statistical methods for analysing primary and secondary outcomes. Reference to where other details of the statistical analysis plan can be found, if not in the protocol | ___10___________ |
| | 20b | Methods for any additional analyses (eg, subgroup and adjusted analyses) | _____NA______ |
| | 20c | Definition of analysis population relating to protocol non-adherence (eg, as randomised analysis), and any statistical methods to handle missing data (eg, multiple imputation) | _____NA_______ |
| **Methods: Monitoring** | | | |
| Data monitoring | 21a | Composition of data monitoring committee (DMC); summary of its role and reporting structure; statement of whether it is independent from the sponsor and competing interests; and reference to where further details about its charter can be found, if not in the protocol. Alternatively, an explanation of why a DMC is not needed | _____NA________ |
| | 21b | Description of any interim analyses and stopping guidelines, including who will have access to these interim results and make the final decision to terminate the trial | _____10-11_____ |
| Harms | 22 | Plans for collecting, assessing, reporting, and managing solicited and spontaneously reported adverse events and other unintended effects of trial interventions or trial conduct | _____11_________ |
| Auditing | 23 | Frequency and procedures for auditing trial conduct, if any, and whether the process will be independent from investigators and the sponsor | _____N/A_______ |
| **Ethics and dissemination** | | | |
| Research ethics approval | 24 | Plans for seeking research ethics committee/institutional review board (REC/IRB) approval | _____11_______ |
| Protocol amendments | 25 | Plans for communicating important protocol modifications (eg, changes to eligibility criteria, outcomes, analyses) to relevant parties (eg, investigators, REC/IRBs, trial participants, trial registries, journals, regulators) | _____11_________ |

| | | | |
|---|---|---|---|
| Consent or assent | 26a | Who will obtain informed consent or assent from potential trial participants or authorised surrogates, and how (see Item 32) | _____11________ |
| | 26b | Additional consent provisions for collection and use of participant data and biological specimens in ancillary studies, if applicable | _____N/A______ |
| Confidentiality | 27 | How personal information about potential and enrolled participants will be collected, shared, and maintained in order to protect confidentiality before, during, and after the trial | _____10_______ |
| Declaration of interests | 28 | Financial and other competing interests for principal investigators for the overall trial and each study site | _____14________ |
| Access to data | 29 | Statement of who will have access to the final trial dataset, and disclosure of contractual agreements that limit such access for investigators | _____11________ |
| Ancillary and post-trial care | 30 | Provisions, if any, for ancillary and post-trial care, and for compensation to those who suffer harm from trial participation | _____N/A_____ |
| Dissemination policy | 31a | Plans for investigators and sponsor to communicate trial results to participants, healthcare professionals, the public, and other relevant groups (eg, via publication, reporting in results databases, or other data sharing arrangements), including any publication restrictions | _____12________ |
| | 31b | Authorship eligibility guidelines and any intended use of professional writers | _____14________ |
| | 31c | Plans, if any, for granting public access to the full protocol, participant-level dataset, and statistical code | _____N/A_______ |
| **Appendices** | | | |
| Informed consent materials | 32 | Model consent form and other related documentation given to participants and authorised surrogates | ____Appendices |
| Biological specimens | 33 | Plans for collection, laboratory evaluation, and storage of biological specimens for genetic or molecular analysis in the current trial and for future use in ancillary studies, if applicable | ___N/A________ |

*It is strongly recommended that this checklist be read in conjunction with the SPIRIT 2013 Explanation & Elaboration for important clarification on the items. Amendments to the protocol should be tracked and dated. The SPIRIT checklist is copyrighted by the SPIRIT Group under the Creative Commons "Attribution-NonCommercial-NoDerivs 3.0 Unported" license.
